# Corrigendum: The OncoPPi network of cancer-focused protein–protein interactions to inform biological insights and therapeutic strategies

**DOI:** 10.1038/ncomms15350

**Published:** 2017-04-18

**Authors:** Zenggang Li, Andrei A. Ivanov, Rina Su, Valentina Gonzalez-Pecchi, Qi Qi, Songlin Liu, Philip Webber, Elizabeth McMillan, Lauren Rusnak, Cau Pham, Xiaoqian Chen, Xiulei Mo, Brian Revennaugh, Wei Zhou, Adam Marcus, Sahar Harati, Xiang Chen, Margaret A. Johns, Michael A. White, Carlos S. Moreno, Lee A. D. Cooper, Yuhong Du, Fadlo R. Khuri, Haian Fu

Nature Communications 8: Article number: 14356; DOI: 10.1038/ncomms14356 (2017); Published: 02 16 2017; Updated: 04 18 2017

The original version of this Article contained an error in the spelling of the author Carlos S. Moreno, which was incorrectly given as Carlos Moreno. This has now been corrected in both the PDF and HTML versions of the Article.

