# Supplementary material for: The OncoPPi network of cancer-focused protein–protein interactions to inform biological insights and therapeutic strategies
Source: Nat Commun. 2017 Feb 16;8:14356. doi: 10.1038/ncomms14356 (PMC5316855; doi:10.1038/ncomms14356)
Supplement: Supplementary Information — Supplementary figures, supplementary tables and supplementary note. [file ncomms14356-s1.pdf]

Supplementary figures

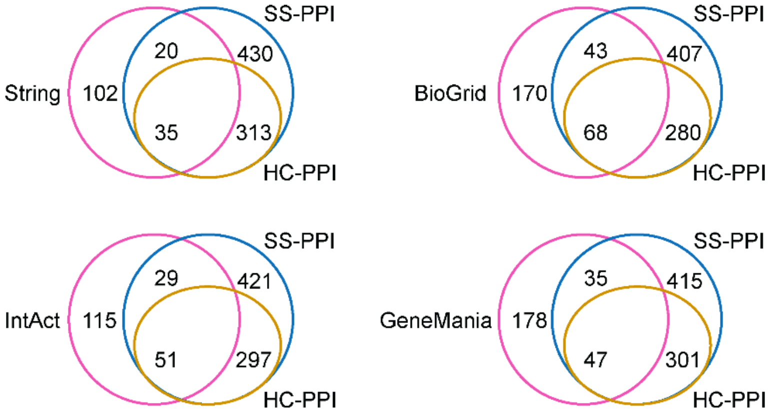

Supplementary Figure 1. The overlap between statistically-significant and high-confidence PPIs with previously reported PPIs in public databases.

Venn diagrams showing the overlap between SS-PPI and HC-PPI sets with PPIs previously reported in String, BioGrid, IntAct, and GeneMania reference PPI databases.

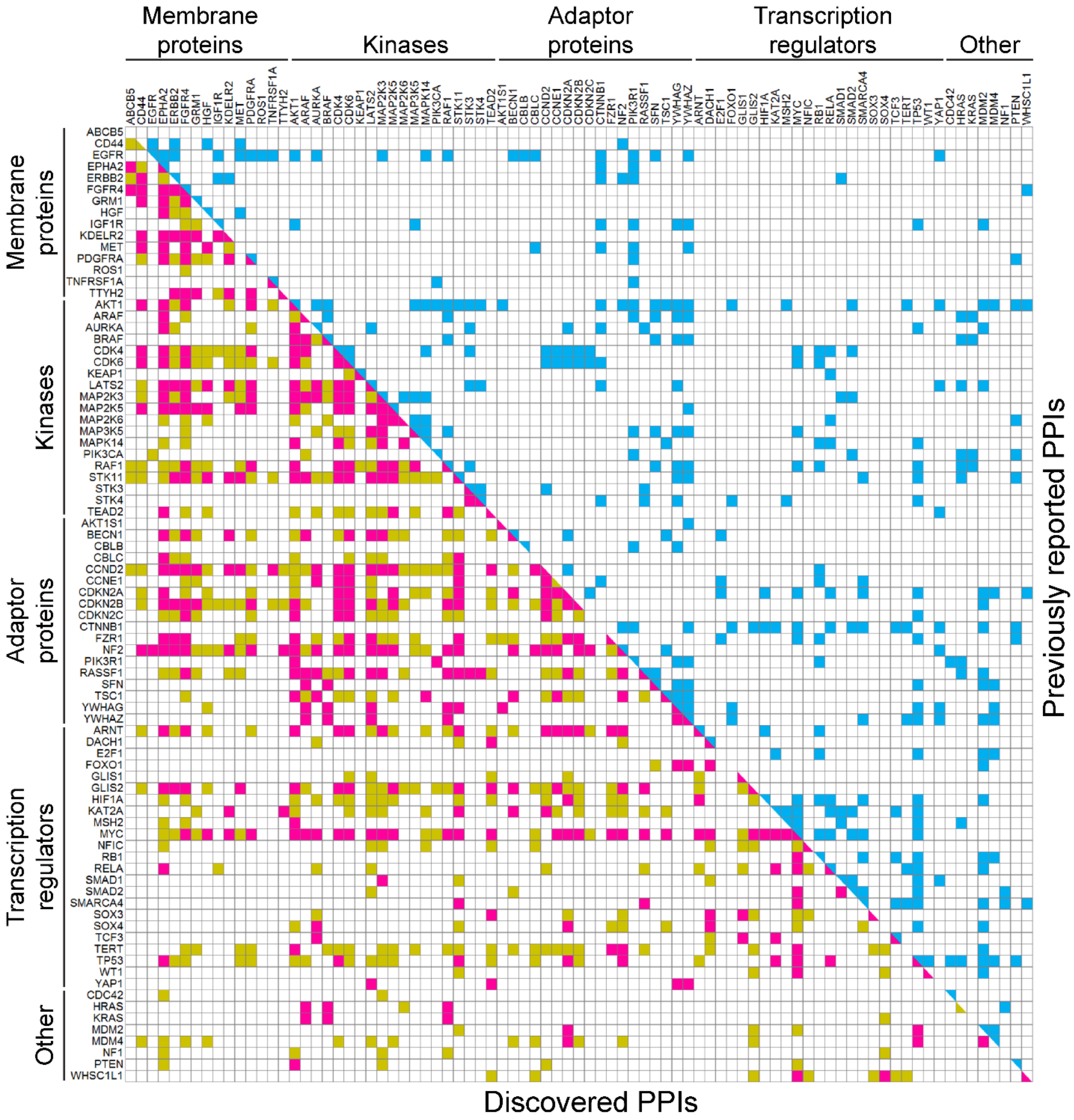

Supplementary Figure 2. Expansion of the PPI landscape for selected lung cancer genes. A heatmap representation of new PPI datasets (magenta OncoPPi PPIs, yellow SS-PPIs) showing expansion beyond the known cancer-associated PPI network (cyan are previously described PPIs).

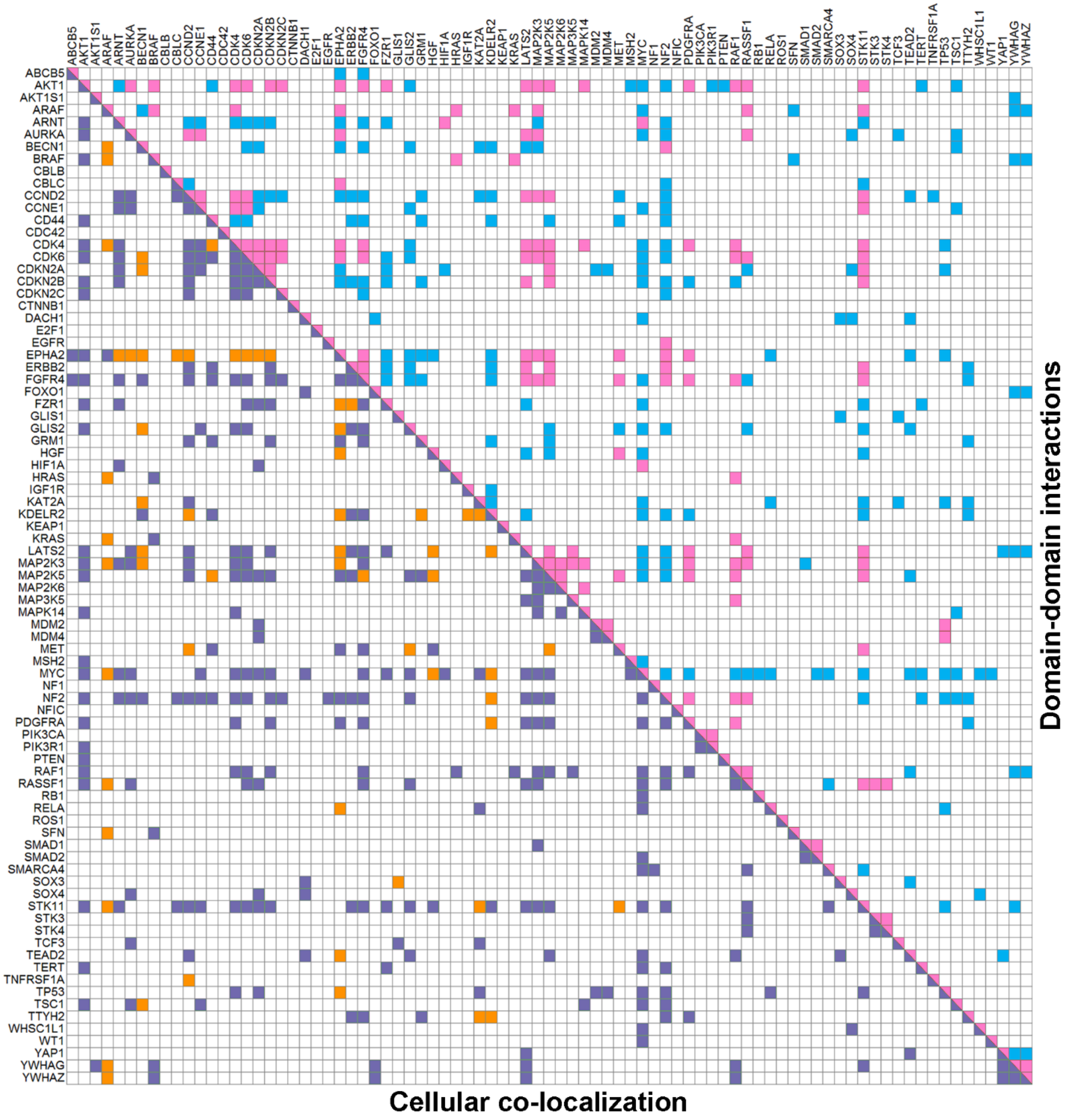

Supplementary Figure 3. Heatmap showing integration of structural and localization data into the OncoPPi network. Known domains involved in co-crystallized PPI complexes indicated in magenta; potential interacting Pfam domains are indicated in cyan; co-localized PPIs are in purple, predicted co-localized PPIs are in orange.

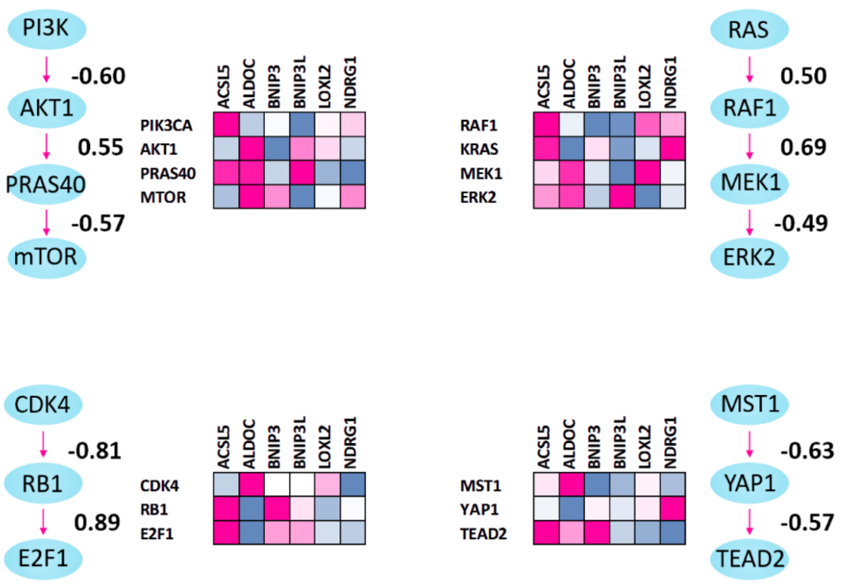

Supplementary Figure 4. FUSION analysis of known PPIs involved in PI3K, RAS/ERK2, CDK4/RB, and Hippo pathways. Heatmaps show the expression of FUSION endogenous reporter genes (*ACSL5*, *ALDOC*, *BNIP3*, *BNIP3L*, *LOXL2*, *NDRG1*) obtained for individual gene knockouts. Blue and magenta colors indicate higher and lower expression, respectively. Pearson correlation values between the expression profiles of the reporter genes obtained for corresponding protein-protein pairs are shown.

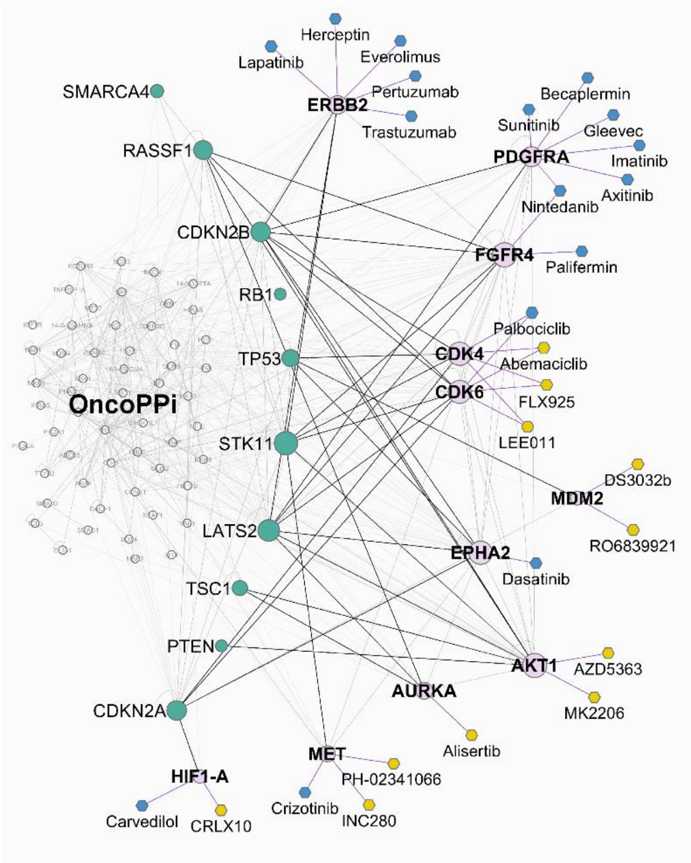

Supplementary Figure 5. The OncoPPi network links tumor suppressors to actionable targets and cancer drugs.

Connections between OncoPPi tumor suppressor genes (cyan), actionable cancer targets (pink circles) and further to FDA approved drugs (blue) or drugs in clinical trials (yellow) are indicated. Drug-target connections are indicated with red lines. Oncogene-tumor suppressor PPIs are indicated with black lines (Supplementary Table 5).

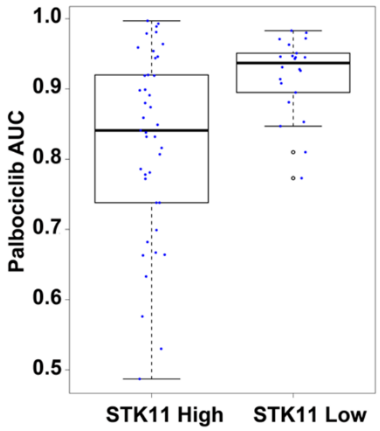

Supplementary Figure 6. STK11 expression correlates with response to CDK4 inhibitor palbociclib in the CCLE dataset. Twenty-two cell lines with reduced copy number of STK11 and normalized mRNA RNAseq levels below 6, (STK11 Low), and 41 cell lines with no loss of STK11 DNA and normalized mRNA RNAseq levels above 7 (STK11 High) were identified in the Cancer Cell Line Encyclopedia (CCLE) database. Analysis of the sensitivity to palbociclib for these two groups of cell lines indicated that those in the STK11 Low group had significantly greater palbociclib sensitivity ($p = 0.002$, two-sided T-test).

Figure 3b

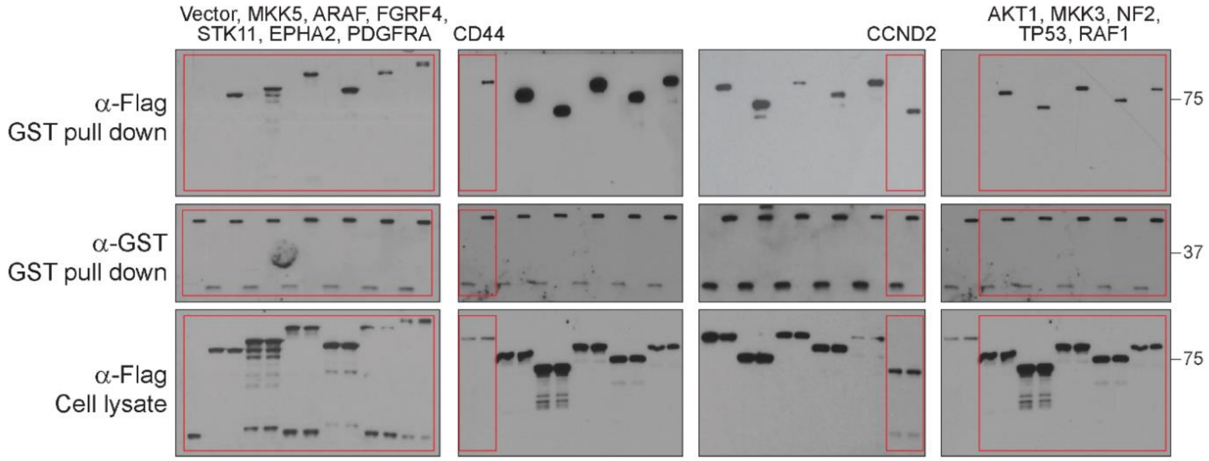

Figure 3c

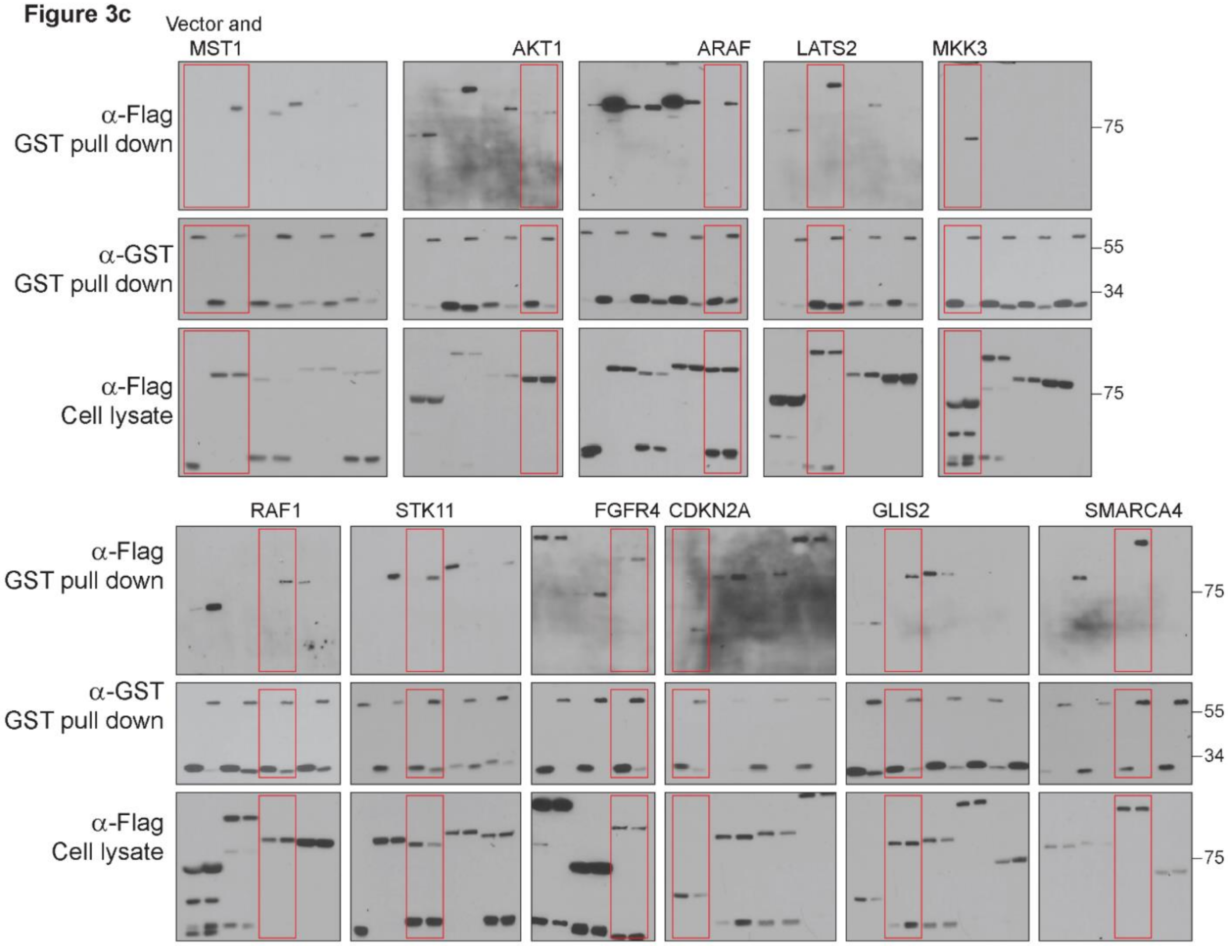

Supplementary Figure 7. Uncropped immunoblots for Figure 3b, c**.** Boxed areas correspond to images presented in the main text.

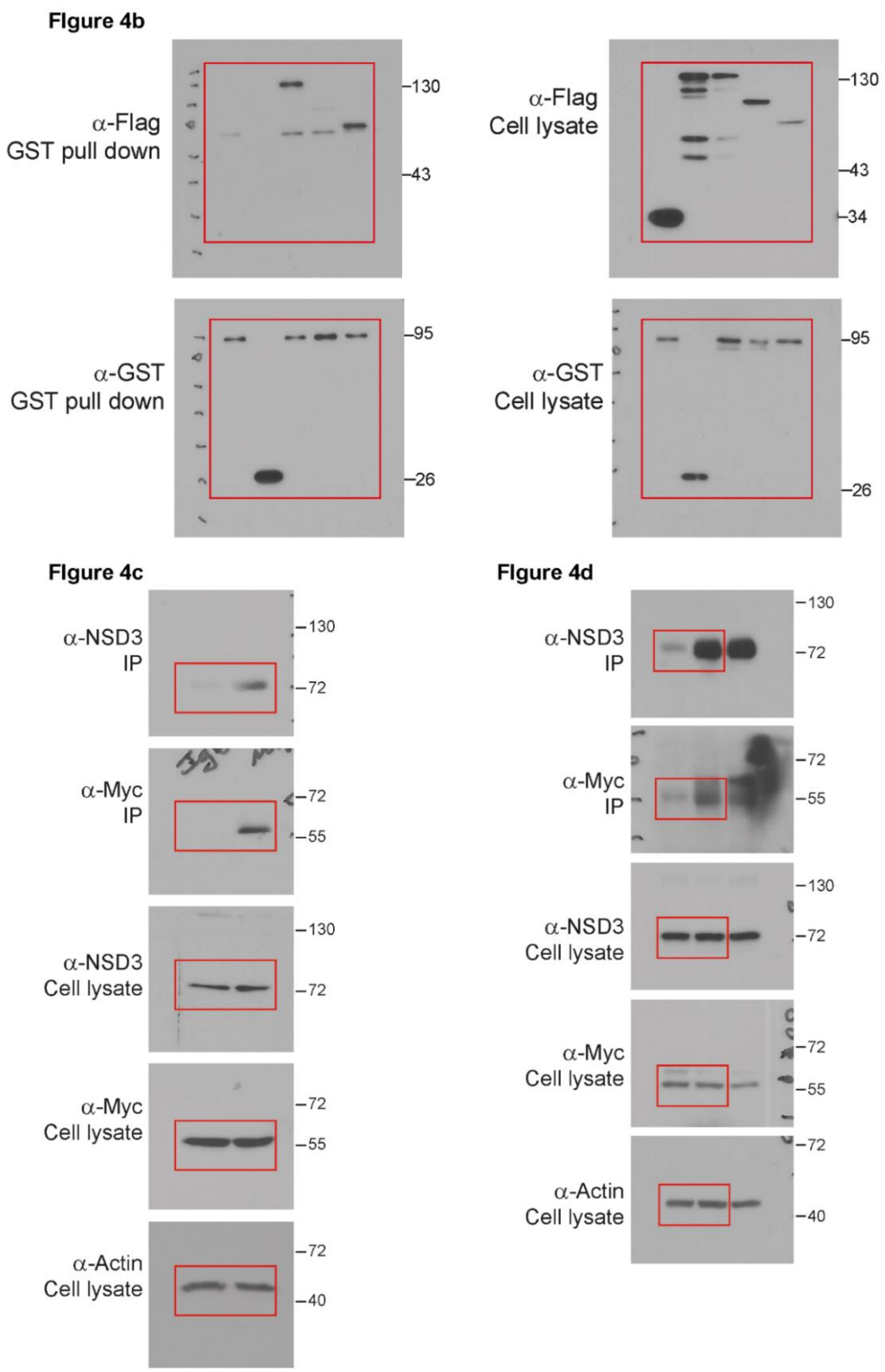

Supplementary Figure 8. Uncropped immunoblots for Figure 4b, c, d**.** Boxed areas correspond to images presented in the main text.

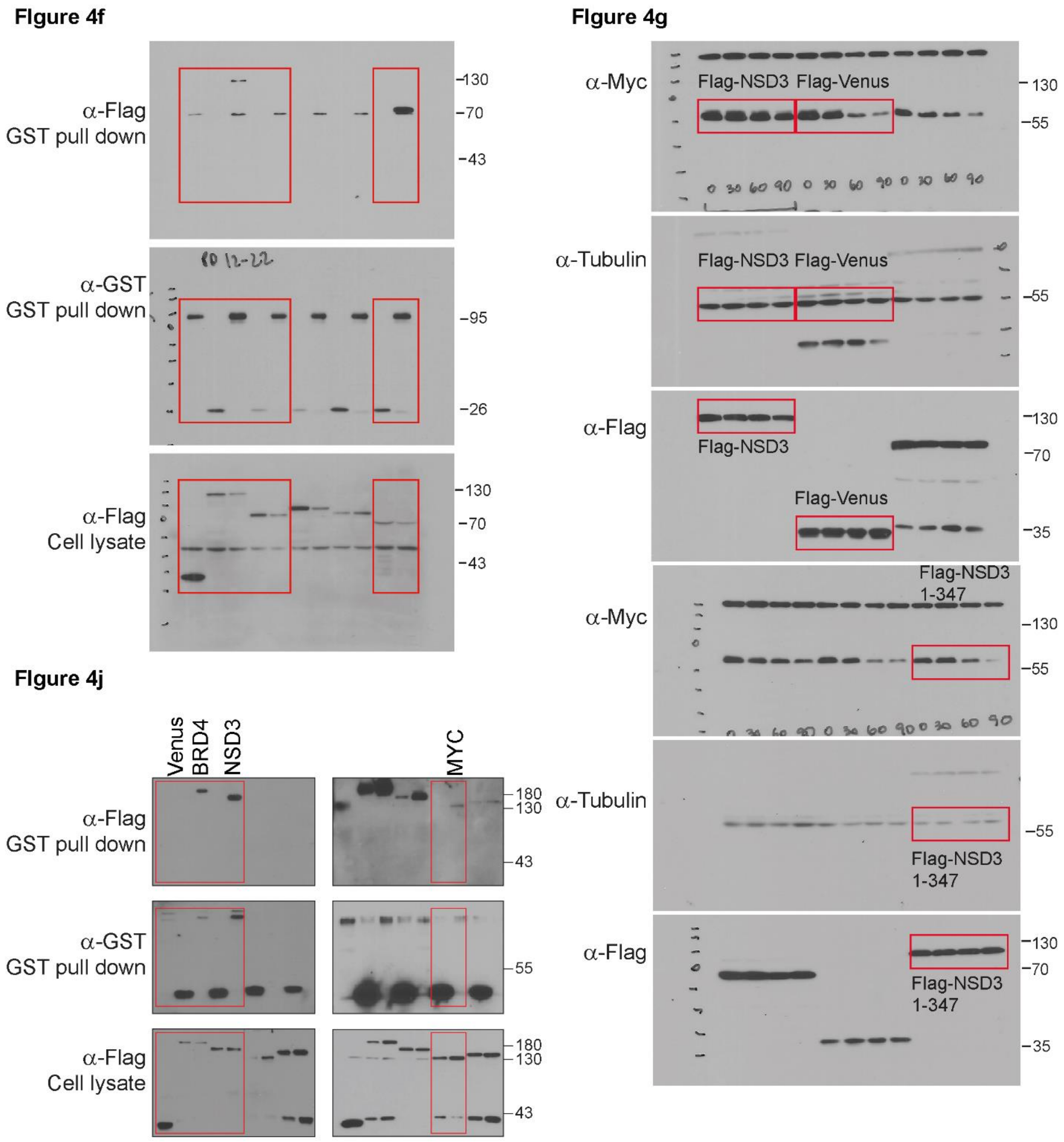

Supplementary Figure 9. Uncropped immunoblots for Figure 4f, g, j**.** Boxed areas correspond to images presented in the main text.

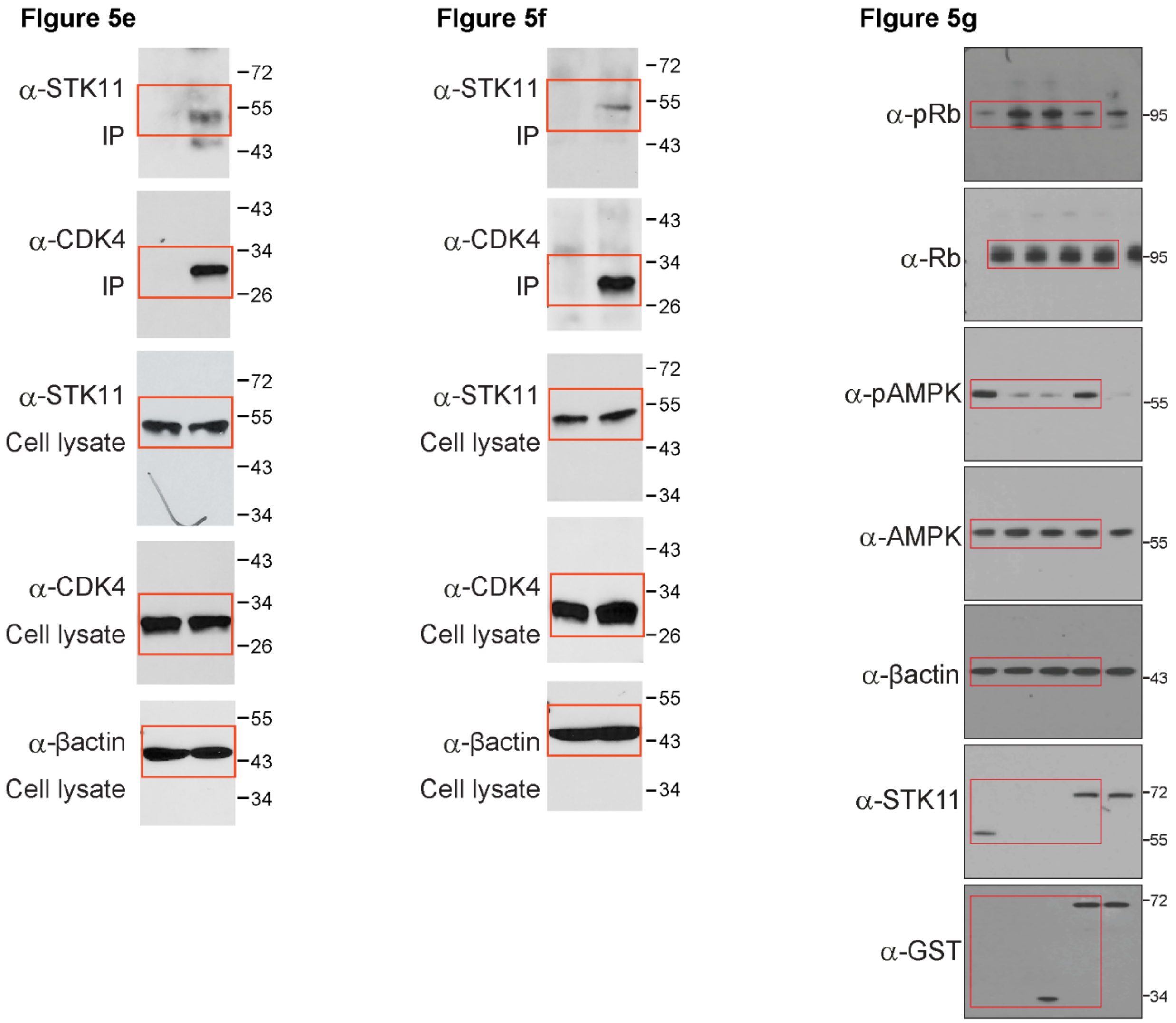

Supplementary Figure 10. Uncropped immunoblots for Figure 5e, f, g. Boxed areas correspond to images presented in the main text.

FIgure 5h

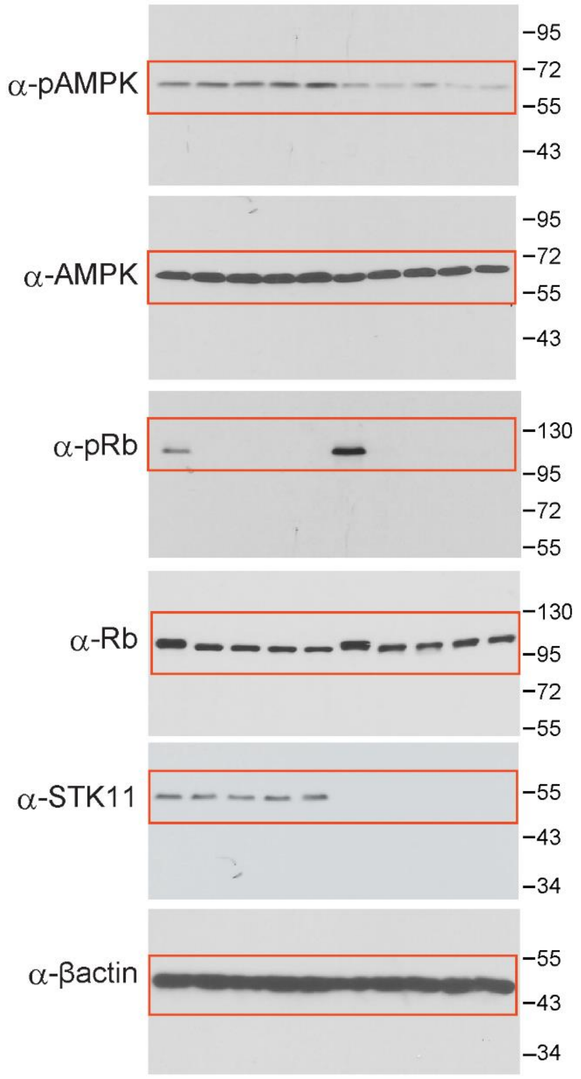

FIgure 5i

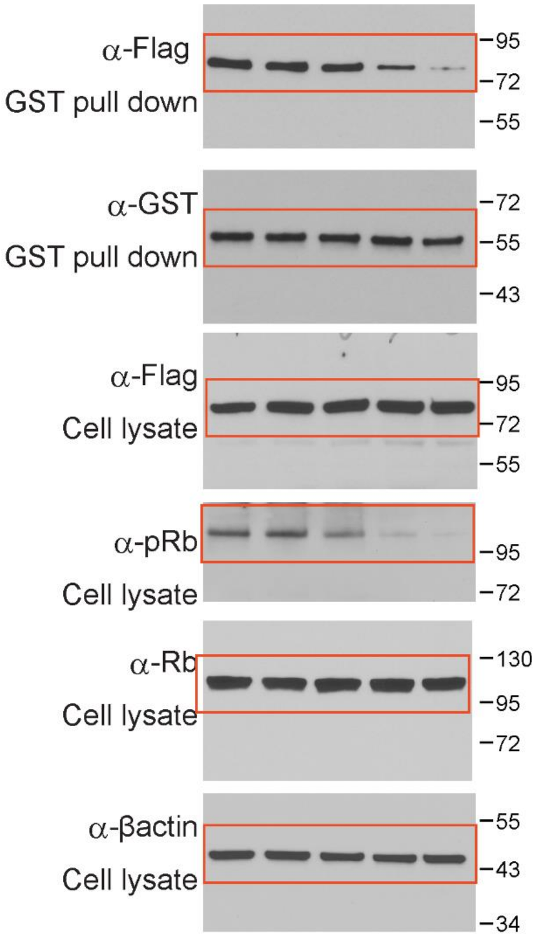

Supplementary Figure 11. Uncropped immunoblots for Figure 5h, i**.** Boxed areas correspond to images presented in the main text.

Supplementary Tables

Supplementary Table 1. Characterization of nodes in the OncoPPi network, related to Figure 2 and 3.

The table includes results from analysis of each node in the OncoPPI network, including the degree, number of novel PPI, number of known PPIs, total number of previously known PPIs, betweenness centrality, number of mutually exclusive PPI and number of PPI with functional connectivity through FUSION analysis.

COMMON NAME	HGNC	DEGREE (TOTAL PPI)	NOVEL PPI	KNOWN PPI	TOTAL NUMBER OF PREVIOUSLY KNOWN PPI	BETWEENNESS CENTRALITY	PPI SUPPORTED BY ME ANALYSIS	PPI SUPOPRTED BY FUSION ANALYSIS
MYC	MYC	37	23	14	16	0.2260	21	14
NF2	NF2	31	26	5	7	0.0732	21	11
AKT1	AKT1	28	16	12	29	0.1292	22	10
EPHA2	EPHA2	28	27	1	4	0.0588	23	8
FGFR4	FGFR4	28	27	1	2	0.0406	21	9
STK11	STK11	28	23	5	8	0.0674	17	8
CCND2	CCND2	24	21	3	5	0.0511	18	6
CDK4	CDK4	24	15	9	13	0.0256	9	9
LATS2	LATS2	24	20	4	9	0.0629	21	11
MKK5	MAP2K5	24	23	1	2	0.0327	21	9
CDK6	CDK6	23	15	8	10	0.0124	19	4
MKK3	MAP2K3	22	17	5	7	0.0569	14	4
CDKN2A	CDKN2A	19	10	9	15	0.0503	12	6
CDKN2B	CDKN2B	19	17	2	3	0.0075	8	4
RAF1	RAF1	18	10	8	15	0.0550	14	4
RASSF1	RASSF1	17	13	4	7	0.0623	0	6
GLIS2	GLIS2	16	16	0	1	0.0081	13	7
HIF1-B	ARNT	16	14	2	3	0.0069	11	1
KDELR2	KDELR2	16	15	1	1	0.0343	13	5
ARAF	ARAF	14	8	6	8	0.0550	9	8
PDGFRA	PDGFRA	14	13	1	4	0.0049	8	3
BECN1	BECN1	13	10	3	5	0.0079	11	5

COMMON NAME	HGNC	DEGREE (TOTAL PPI)	NOVEL PPI	KNOWN PPI	TOTAL NUMBER OF PREVIOUSLY KNOWN PPI	BETWEENNESS CENTRALITY	PPI SUPPORTED BY ME ANALYSIS	PPI SUPOPRTED BY FUSION ANALYSIS
FZR1	FZR1	13	12	1	5	0.0036	10	3
AURKA	AURKA	12	9	3	10	0.0416	11	5
ERBB2	ERBB2	12	9	3	9	0.0027	11	3
CCNE1	CCNE1	11	9	2	6	0.0022	10	1
TP53	TP53	11	3	8	30	0.0214	7	2
14-3-3GAMMA	YWHAG	10	1	9	19	0.0412	9	0
CD44	CD44	10	7	3	5	0.0028	7	2
TEAD2	TEAD2	10	9	1	1	0.0353	5	5
GCN5	KAT2A	9	5	4	10	0.0193	7	3
14-3-3ZETA	YWHAZ	8	1	7	24	0.0079	6	4
BRAF	BRAF	8	0	8	9	0.0113	6	2
GRM1	GRM1	8	8	0	1	0.0010	4	4
MET	MET	8	6	2	7	0.0009	7	3
TSC1	TSC1	8	3	5	7	0.0028	6	4
TTYH2	TTYH2	8	8	0	0	0.0012	4	0
CDKN2C	CDKN2C	6	4	2	4	0.0003	5	1
DACH1	DACH1	6	5	1	1	0.0189	3	1
HGF	HGF	6	5	1	3	0.0008	4	3
P38	MAPK14	5	0	5	11	0.0016	5	4
P65	RELA	5	1	4	13	0.0002	2	3
ASK1	MAP3K5	4	1	3	12	0.0001	2	0
CBLC	CBLC	4	4	0	2	0.0000	3	1
MKK6	MAP2K6	4	2	2	5	0.0002	2	2
SOX3	SOX3	4	4	0	0	0.0090	2	1
SOX4	SOX4	4	4	0	2	0.0031	3	2
TCF3	TCF3	4	2	2	5	0.0182	3	0
TERT	TERT	4	3	1	6	0.0001	2	1
YAP1	YAP1	4	0	4	9	0.0014	4	2
14-3-3SIGMA	SFN	3	0	3	13	0.0000	1	1
FOXO1	FOXO1	3	1	2	6	0.0024	3	1
GLIS1	GLIS1	3	3	0	0	0.0007	2	0
HIF1-A	HIF1A	3	0	3	10	0.0000	2	1
H-RAS	HRAS	3	0	3	11	0.0003	2	0

COMMON NAME	HGNC	DEGREE (TOTAL PPI)	NOVEL PPI	KNOWN PPI	TOTAL NUMBER OF PREVIOUSLY KNOWN PPI	BETWEENNESS CENTRALITY	PPI SUPPORTED BY ME ANALYSIS	PPI SUPOPRTED BY FUSION ANALYSIS
K-RAS	KRAS	3	0	3	4	0.0003	3	2
MDM2	MDM2	3	0	3	22	0.0000	3	2
MDM4	MDM4	3	0	3	10	0.0000	3	1
MST1	STK4	3	0	3	10	0.0000	1	1
MST2	STK3	3	0	3	9	0.0000	1	1
SMARCA4	SMARCA4	3	1	3	16	0.0000	2	3
WHSC1L1	WHSC1L1	3	3	0	4	0.0009	2	1
ABCB5	ABCB5	2	2	0	0	0.0000	1	0
MSH2	MSH2	2	0	2	8	0.0000	1	1
PIK3R1	PIK3R1	2	0	2	19	0.0270	2	0
PRAS40	AKT1S1	2	2	0	2	0.0000	1	0
SMAD1	SMAD1	2	0	2	8	0.0006	1	1
SMAD2	SMAD2	2	0	2	12	0.0023	1	1
TNFRSF1A	TNFRSF1A	2	1	1	4	0.0000	0	0
WT1	WT1	2	1	0	2	0.0000	1	0
EGFR	EGFR	1	1	0	22	0.0000	1	1
IGF1R	IGF1R	1	1	0	11	0.0000	1	0
KEAP1	KEAP1	1	0	1	2	0.0000	0	0
NFIC	NFIC	1	1	0	0	0.0000	0	0
PIK3CA	PIK3CA	1	0	1	8	0.0000	1	0
PTEN	PTEN	1	0	1	9	0.0000	1	0
RB1	RB1	1	0	1	18	0.0000	1	0
CBLB	CBLB	0	0	0	4	NA	0	0
CDC42	CDC42	0	0	0	3	NA	0	0
CTNNB1	CTNNB1	0	0	0	27	NA	0	0
E2F1	E2F1	0	0	0	9	NA	0	0
NF1	NF1	0	0	1	3	0.0000	1	0
ROS1	ROS1	0	0	0	1	NA	0	0

COMMON NAME - Common protein name; HGNC - Standard HUGO gene symbol; DEGREE (TOTAL PPI) - Total number of PPIs; NOVEL PPI - The number of novel PPIs; KNOWN PPI - The number of previously reported PPI within the 83 tested proteins; TOTAL NUMBER OF PREVIOUSLY KNOWN PPI - Total number of previously reported PPIs; BETWEENNESS CENTRALITY - The Betwenness centrality index; PPI SUPPORTED BY ME ANALYSIS - The number of PPIs supported by mutual exclusivity of genomic alterations; PPI SUPOPRTED BY FUSION ANALYSIS - The number of PPIs supported by Fusion analysis.

Supplementary Table 2. Structural Pfam domains in OncoPPi proteins, related to Figure 2.

The table includes a list of the structural domains extracted from Pfam for each node in the OncoPPi network.

HGNC	Protein structural domains extracted from Pfam
YWHAG	14-3-3; DUF722; DUF837; Orbi_VP5; SF3A2; Peptidase_C98; TPR_12
SFN	14-3-3; DUF837; Peptidase_C98; TPR_12
YWHAZ	14-3-3; FliM; Peptidase_C98; TPR_12
ABCB5	ABC_tran; ABC_membrane; DUF2207
AKT1	Pkinase_C; Pkinase; PH
ARAF	Pkinase_Tyr; C1_1; RBD; zf-RING-like
MAP3K5	Pkinase; DUF4071
AURKA	Pkinase; MTBP_C
BECN1	APG6; BH3; FTA4; Exonuc_VII_L; HAUS-augmin3; LMBR1; ERM; Suppressor_APC; MT
BRAF	RBD; C1_1; zf-RING-like; Pkinase_Tyr
CBLB	Cbl_N; Cbl_N2; Cbl_N3; zf-C3HC4_3; UBA
CBLC	Cbl_N; Cbl_N2; Cbl_N3; SH2; zf-C3HC4_3
CCND2	Cyclin_N; K-cyclin_vir_C; Cyclin_C
CCNE1	Cyclin_N; Cyclin_C
CD44	Xlink
CDC42	Ras; PET117
CDK4	Pkinase
CDK6	Pkinase; Uricase
CDKN2A	Ank_5
CDKN2B	Ank_5
CDKN2C	DUF1843; Ank_4; Ank_5
CTNNB1	HEAT_2; Queuosine_synth; Ric8; Arm; WAPL; Atx10homo_assoc
DACH1	Ski_Sno
E2F1	E2F_CC-MB; E2F_TDP
EGFR	Pkinase_Tyr; GF_recep_IV; Recep_L_domain; Furin-like; GAPT
EPHA2	SAM_1; Pkinase_Tyr; EphA2_TM; fn3; Ephrin_lbd; GCC2_GCC3; DUF2369; SKG6
ERBB2	Pkinase_Tyr; GF_recep_IV; Recep_L_domain; Furin-like
FGFR4	Pkinase_Tyr; I-set
FOXO1	FOXO-TAD; FOXO_KIX_bdg; Forkhead
FZR1	WD40
KAT2A	Bromodomain; Acetyltransf_7; PCAF_N; T2SSM
GLIS1	FOXP-CC; zf-H2C2_2; zf-C2H2
GLIS2	FOXP-CC; zf-H2C2_2; zf-C2H2
GRM1	GluR_Homer-bdg; 7tm_3; NCD3G; ANF_receptor; AAA_34; DUF2318

HGNC	Protein structural domains extracted from Pfam
HGF	Trypsin; Kringle; PAN_1
HIF1A	HIF-1a_CTAD; HIF-1; PAS_3; PAS; HLH
ARNT	PAS_11; PAS; HLH; PRAS
HRAS	Ras; Ldh_1_N
IGF1R	Pkinase_Tyr; Recep_L_domain; Furin-like; fn3; DUF4476
KDELR2	ER_lumen_recept
KEAP1	Kelch_1; BACK; BTB
KRAS	Ras
LATS2	Pkinase; UBA; Pkinase_C
MDM2	zf-C3HC4_3; zf-RanBP; SWIB; Pinin_SDK_N; zf-FPG_IleRS
MDM4	zf-C3HC4_3; zf-RanBP; SWIB; Cript; Cytochrom_c3_2
MET	Pkinase_Tyr; TIG; PSI; Sema
MAP2K3	Pkinase; DUF4344; DHR-2
MAP2K5	Pkinase; PB1
MAP2K6	Pkinase; DUF4344
MSH2	MutS_V; MutS_IV; MutS_III; MutS_II; MutS_I; ArdA
STK4	Mst1_SARAH; Pkinase; Abi_2; DUF4611
STK3	Mst1_SARAH; Pkinase; Abi_2
MYC	Myc-LZ; HLH; Myc_N; TRH
NF1	CRAL_TRIO_2; RasGAP; MIF4G_like_2; MOR2-PAG1_C
NF2	ERM; FERM_C; FERM_M; FERM_N
NFIC	CTF_NFI; MH1; NfI_DNAbd_pre-N
MAPK14	Pkinase; NAD_binding_8
RELA	RHD_dimer; RHD_DNA_bind
PDGFRA	Pkinase_Tyr; I-set; ig; Herpes_gE; LAT
PIK3CA	PF00454.23; PF00613.16; PF00792.20; PF00794.14; PF02192.12; PI3K_p85B; PI3K_rbd; ASC; PI3K_C2; PI3Ka; DUF2321; Ribosomal_S15; PI3_PI4_kinase; DUF4135
PIK3R1	SH2; PI3K_P85_iSH2; RhoGAP; SH3_2; Laminin_I; Lant_dehydr_C; Transcrip_act; Mod_r
AKT1S1	PRAS
PTEN	PTEN_C2; DSPc; DUF3256
RAF1	Pkinase_Tyr; C1_1; RBD; zf-RING-like
RASSF1	Nore1-SARAH; RA; C1_1; Mst1_SARAH
RB1	Rb_C; RB_B; RB_A; DUF3452
ROS1	fn3; Pkinase_Tyr
SMAD1	MH2; MH1; DUF4223
SMAD2	MH1; MH2
SMARCA4	QLQ; DUF2009; HSA; BRK; SNF2_N; Helicase_C; SnAC; Bromodomain
SOX3	SOXp; HMG_box
SOX4	HMG_box
STK11	Pkinase
TCF3	HLH; CDC45; DUF4349
TEAD2	TEA

HGNC	Protein structural domains extracted from Pfam
TERT	RVT_1; Telomerase_RBD
TNFRSF1A	Death; TNFR_c6
TP53	P53_tetramer; P53; P53_TAD
TSC1	Hamartin
TTYH2	Tweety; DUF4577; DUF4131
WHSC1L1	SET; PWWP; PHD; zf-HC5HC2H_2
WT1	zf-C2H2; Ofd1_CTDD; zf-H2C2_2; FOXP-CC
YAP1	WW; FAM181; MbtH

HGNC – Standard HUGO gene symbol

Supplementary Table 3. Expression of FUSION reporter genes obtained for the individual gene knock downs, related to Figure 3, 5 and Supplementary Figure 4

The table includes the Pearson correlation values for mRNA expression of each node in the OncoPPi network with the FUSION reporter genes (ACSL5, ALDOC, BNIP3, BNIP3L, LOXL2, NDRG1) based on large-scale profiling of individual gene knockdowns with siRNA in HCT116 cells as described in Potts, et al, 2013.

HGNC	ACSL5	ALDOC	BNIP3	BNIP3L	LOXL2	NDRG1
MYC	-0.4106	0.7198	0.6470	-0.0077	-1.4627	-1.1279
AKT1	0.2149	-1.0593	0.5113	-0.4917	-0.1245	0.1993
RAF1	-1.6416	-0.9802	0.0816	-0.0173	-1.4514	-1.2988
PDGFRA	-0.0297	-1.7982	-1.1210	-0.4821	-1.5715	-1.6835
ARAF	-0.1206	-1.1771	-0.1943	-0.4599	0.5696	0.1983
AURKA	0.0791	1.9412	0.5052	1.2013	0.1475	-0.0994
ERBB2	-0.0423	-0.2464	-0.6781	0.1826	0.3081	-0.2259
MET	-0.0335	1.7244	-0.0331	0.5920	-0.4668	0.7607
BRAF	0.1639	0.0438	-0.4151	-0.2205	-0.5330	-0.3729
CBLC	1.3836	0.3168	0.7943	0.0791	-0.0715	0.9855
MDM2	-0.1929	-0.8533	-0.0904	-0.1760	-0.5222	-0.3916
HRAS	-0.4833	0.0024	0.1546	-0.3100	0.2521	-0.8080
KRAS	-0.4091	1.1423	0.4254	1.0441	0.7019	-0.5313
WHSC1L1	-1.0540	1.1066	-0.0171	0.3833	0.5312	-0.5143
TCF3	-0.0321	-0.5966	-0.2156	-0.7141	-0.6492	-0.9417
FOXO1	0.3439	-0.1858	-0.1402	-0.8611	-0.5552	0.3511
YAP1	0.2249	1.2669	0.0127	0.3281	-0.0381	-1.9592
PIK3CA	-1.1563	0.0800	-0.2655	0.5974	-0.3364	-0.4607
EGFR	-0.5873	-0.4883	-0.2599	-0.1114	0.3112	-0.4416
CTNNB1	0.2803	0.3189	-0.0222	-0.6478	0.8598	0.5234
CBLB	0.2670	0.9792	0.0696	0.2352	-0.5074	-0.8933
ROS1	0.0496	0.1291	-0.0925	-1.3249	-0.4503	-0.4196
NF2	-0.5629	1.4314	-0.2365	-0.0179	-1.4120	-0.6191
STK11	-0.2199	-0.0177	0.0586	-0.7193	-0.2505	-0.7014
LATS2	0.0265	-0.8021	0.0367	-0.1223	-0.2400	-0.2729
CDKN2B	-0.0020	0.4795	0.1838	-1.0927	0.1033	-0.6169
RASSF1	0.6135	1.1784	0.0448	1.0334	0.1098	1.3387
CDKN2A	-1.4332	-1.3134	-0.7976	0.1450	0.0786	-0.3931
TSC1	-1.1586	0.1624	0.3377	-0.5317	-0.2363	-1.1541
TP53	-0.1531	-0.7476	0.2847	0.4781	0.3491	-1.1799
SMARCA4	-0.1006	-0.0807	0.0676	-0.1044	-0.2183	-0.5665
PIK3R1	1.1369	0.3054	0.8242	1.2634	0.3423	-0.3556
WT1	-0.0363	0.2594	0.0861	-0.4994	0.7577	0.1674
RB1	-0.9017	1.2577	-0.8870	-0.3662	0.5842	-0.2380
PTEN	0.1627	0.3811	0.1365	-0.5365	-0.4033	-0.0042
SMAD2	0.2553	0.8525	0.2215	0.3469	0.5198	-0.6401

HGNC	ACSL5	ALDOC	BNIP3	BNIP3L	LOXL2	NDRG1
MSH2	-0.2915	0.2616	0.3276	0.2084	-0.1826	-0.5869
NF1	0.2617	-0.4561	-0.2103	-0.7987	-0.3906	-0.6829
FGFR4	-0.3103	0.7378	-0.1192	0.5015	0.4393	0.2466
EPHA2	-1.0094	-0.7031	-0.2507	-0.4357	0.2374	-0.4364
MAP2K5	0.4663	-0.4099	0.7860	-0.8659	-0.1998	0.0643
CCND2	-0.0354	0.5776	0.6686	0.4143	-0.5065	0.7369
CDK6	0.8398	0.7801	0.3581	-0.3585	0.6622	1.5399
CDK4	-0.0267	-1.0939	-0.1980	-0.1965	-0.4601	0.2458
MAP2K3	1.5914	1.9273	0.2716	0.2519	1.0720	2.1795
GLIS2	-0.1741	1.3693	0.2034	0.3847	0.0028	-0.4033
ARNT	-0.4988	-0.0432	0.1750	-0.4076	-0.4501	-0.0123
KDELR2	-0.9309	0.2360	0.2060	0.1629	-0.0200	-0.2586
BECN1	-0.1434	1.2006	0.0343	0.0005	-0.2103	-0.8562
FZR1	-0.1050	-0.0127	-0.0622	0.3145	-0.1489	1.5306
CCNE1	-0.6616	-0.1286	-0.3588	-0.3849	1.1788	-0.5096
TEAD2	-0.7553	-0.4304	-0.7469	-0.0222	0.1454	0.3201
CD44	-1.0420	0.1504	0.2718	-1.0599	0.0084	-0.1576
GRM1	0.1060	-0.6006	-0.0148	-0.5190	-0.8127	-0.4283
KAT2A	0.4252	0.5475	0.0395	-0.0920	-0.5182	0.5833
CDKN2C	0.3554	-0.0244	-0.4375	-0.5639	-0.6480	0.0196
DACH1	0.0854	-0.0393	-0.3339	-0.3839	-0.2810	0.6201
YWHAZ	-0.2384	-0.5512	-0.0514	-0.1039	0.0846	0.0586
HGF	0.0099	-0.1025	0.1696	-0.0705	-0.2051	-0.1371
MAP2K6	0.1489	0.5438	0.9704	0.8389	0.5499	-1.0433
SOX4	-0.1931	0.1518	-0.0769	0.5065	0.4479	0.0575
SOX3	0.7031	0.2996	0.3548	0.6747	1.3216	1.2183
MAPK14	-0.2461	0.3140	0.0121	-0.2642	-0.1417	-0.6667
TERT	0.6798	0.7571	0.0419	1.1114	0.1978	0.4595
SFN	-0.4799	-4.9339	-0.4645	-0.8516	-2.5254	-0.7103
HIF1A	-1.1537	-0.2355	-0.3908	-0.2105	-0.6616	-1.7372
AKT1S1	-0.4863	-0.5087	-0.0053	-0.5362	0.1746	0.4085
ABCB5	-0.1113	0.2140	0.1776	0.4645	0.1075	-0.3759
IGF1R	0.0773	0.2247	0.1753	-0.2403	0.3783	1.1284
MAP3K5	0.0545	0.1118	-0.1311	-0.3071	-0.9349	-0.4443
RELA	0.4420	-0.1258	-0.1484	-0.0321	1.2526	0.3823
MDM4	0.5883	-0.0427	0.1071	0.2880	-0.2917	-0.1565
STK4	-0.0332	-1.7757	0.3584	0.2782	0.0430	0.2654
STK3	0.4003	-0.0116	0.7980	0.9515	0.0292	0.1250
KEAP1	-0.5409	1.3747	0.4001	-0.8790	-1.0300	-0.0230
NFIC	0.1610	-1.0668	-0.0837	-0.1544	0.1968	-1.5950
TNFRSF1A	0.4119	0.8244	-1.0215	0.7740	-0.1712	0.6682
E2F1	-0.2124	1.9955	0.4030	0.4244	1.1188	1.2978
SMAD1	0.5365	1.2468	1.1687	0.8700	0.0047	-0.0005
CDC42	-0.1147	-0.5118	0.0682	-0.2470	-0.0017	0.6047

HGNC - Standard HUGO gene symbol

Supplementary Table 4. Correlations between expression signatures for microRNA and OncoPPi genes, related to Figure 3, 5 and Supplementary Figure 4

The table includes a list of genes from FUSION analysis which have expression signatures that correlate with each node in the OncoPPi network. PPIs are scored as positive in FUSION analysis if the absolute value of the Pearson correlation coefficient (R) is greater than or equal to 0.5.

GENE1	GENE2	R	Positive
ABCB5	APC	-0.0482	0
ABCB5	BAX	0.6617	1
ABCB5	BCL2	0.6511	1
ABCB5	CDC42	-0.7574	1
ABCB5	DVL1	0.8944	1
ABCB5	E2F1	-0.0415	0
ABCB5	GSK3B	0.9273	1
ABCB5	IGF1R	-0.8454	1
ABCB5	JAK1	0.1162	0
ABCB5	JAK2	-0.0898	0
ABCB5	KEAP1	-0.0224	0
ABCB5	MAP2K1	0.3388	0
ABCB5	MAP2K4	0.5667	1
ABCB5	MAP3K5	0.0887	0
ABCB5	MAPK1	-0.2679	0
ABCB5	MAPK3	0.7487	1
ABCB5	MAPK8	-0.4236	0
ABCB5	MAPK9	0.1277	0
ABCB5	MDM4	0.1435	0
ABCB5	MTOR	0.4427	0
ABCB5	NFIC	0.4595	0
ABCB5	NFKB2	-0.6073	1
ABCB5	NOTCH1	0.1653	0
ABCB5	PRKAA2	0.4789	0
ABCB5	RELA	-0.3447	0
ABCB5	SMAD1	0.6440	1
ABCB5	SMAD4	-0.1915	0
ABCB5	STAT1	0.7773	1
ABCB5	STAT2	0.0455	0
ABCB5	STK3	0.5219	1
ABCB5	STK4	-0.1781	0
ABCB5	TNFRSF1A	-0.0754	0
AKT1	ABCB5	-0.5032	1
AKT1	AKT1S1	0.5466	1

GENE1	GENE2	R	Positive
AKT1	APC	-0.2907	0
AKT1	ARAF	0.6854	1
AKT1	ARNT	0.1587	0
AKT1	AURKA	-0.8573	1
AKT1	BAX	0.0553	0
AKT1	BCL2	-0.6402	1
AKT1	BECN1	-0.7458	1
AKT1	BRAF	-0.3695	0
AKT1	CBLB	-0.6181	1
AKT1	CBLC	0.6001	1
AKT1	CCND2	-0.0284	0
AKT1	CCNE1	-0.1858	0
AKT1	CD44	0.0093	0
AKT1	CDC42	0.7197	1
AKT1	CDK4	0.7936	1
AKT1	CDK6	0.2332	0
AKT1	CDKN2A	0.0268	0
AKT1	CDKN2B	-0.0787	0
AKT1	CDKN2C	0.0693	0
AKT1	CTNNB1	0.1298	0
AKT1	DACH1	0.1808	0
AKT1	DVL1	-0.2809	0
AKT1	E2F1	-0.6292	1
AKT1	EGFR	-0.0116	0
AKT1	EPHA2	0.1433	0
AKT1	ERBB2	-0.3537	0
AKT1	FGFR4	-0.8593	1
AKT1	FOXO1	0.4577	0
AKT1	FZR1	0.1717	0
AKT1	GLIS2	-0.8323	1
AKT1	GRM1	0.6312	1
AKT1	GSK3B	-0.3700	0
AKT1	HGF	0.5013	1
AKT1	HIF1A	-0.5252	1

GENE1	GENE2	R	Positive
AKT1	HRAS	-0.1855	0
AKT1	IGF1R	0.2912	0
AKT1	JAK1	0.0520	0
AKT1	JAK2	0.4760	0
AKT1	KAT2A	-0.0963	0
AKT1	KDELR2	-0.4305	0
AKT1	KEAP1	-0.3477	0
AKT1	KRAS	-0.7262	1
AKT1	LATS2	0.8355	1
AKT1	MAP2K1	0.1509	0
AKT1	MAP2K3	-0.1998	0
AKT1	MAP2K4	0.2033	0
AKT1	MAP2K5	0.8065	1
AKT1	MAP2K6	-0.2321	0
AKT1	MAP3K5	-0.1827	0
AKT1	MAPK1	0.4873	0
AKT1	MAPK14	-0.5256	1
AKT1	MAPK3	-0.6708	1
AKT1	MAPK8	0.6635	1
AKT1	MAPK9	-0.7072	1
AKT1	MDM2	0.7524	1
AKT1	MDM4	0.1657	0
AKT1	MET	-0.7239	1
AKT1	MSH2	-0.3841	0
AKT1	MTOR	-0.0898	0
AKT1	NF1	0.4192	0
AKT1	NF2	-0.6941	1
AKT1	NFIC	0.2560	0
AKT1	NFKB2	0.5135	1
AKT1	NOTCH1	-0.1296	0
AKT1	PDGFRA	0.2860	0
AKT1	PIK3CA	-0.6034	1
AKT1	PIK3R1	0.0271	0
AKT1	PRKAA2	-0.0234	0
AKT1	PTEN	-0.0185	0
AKT1	RAF1	-0.0028	0
AKT1	RASSF1	-0.5115	1
AKT1	RB1	-0.8130	1
AKT1	RELA	0.2029	0
AKT1	ROS1	0.1227	0
AKT1	SFN	0.8319	1
AKT1	SMAD1	-0.3537	0
AKT1	SMAD2	-0.6354	1
AKT1	SMAD4	-0.3323	0
AKT1	SMARCA4	-0.0993	0
AKT1	SOX3	0.2730	0
AKT1	SOX4	-0.5279	1
AKT1	STAT1	-0.2188	0
AKT1	STAT2	-0.4265	0

GENE1	GENE2	R	Positive
AKT1	STK11	0.0161	0
AKT1	STK3	0.2951	0
AKT1	STK4	0.8002	1
AKT1	TCF3	0.3793	0
AKT1	TEAD2	-0.1650	0
AKT1	TERT	-0.6336	1
AKT1	TNFRSF1A	-0.6540	1
AKT1	TP53	0.1408	0
AKT1	TSC1	-0.2793	0
AKT1	WHSC1L1	-0.7729	1
AKT1	WT1	0.0324	0
AKT1	YAP1	-0.6134	1
AKT1	YWHAZ	0.6809	1
AKT1S1	ABCB5	-0.6163	1
AKT1S1	APC	-0.0527	0
AKT1S1	BAX	-0.5539	1
AKT1S1	BCL2	-0.1398	0
AKT1S1	CDC42	0.8705	1
AKT1S1	DVL1	-0.4510	0
AKT1S1	E2F1	0.2391	0
AKT1S1	GSK3B	-0.5214	1
AKT1S1	IGF1R	0.8344	1
AKT1S1	JAK1	-0.7235	1
AKT1S1	JAK2	0.1631	0
AKT1S1	KEAP1	-0.1450	0
AKT1S1	MAP2K1	-0.2394	0
AKT1S1	MAP2K4	-0.5121	1
AKT1S1	MAP3K5	-0.6687	1
AKT1S1	MAPK1	0.7741	1
AKT1S1	MAPK3	-0.4139	0
AKT1S1	MAPK8	0.6992	1
AKT1S1	MAPK9	0.0063	0
AKT1S1	MDM4	-0.7004	1
AKT1S1	MTOR	-0.5748	1
AKT1S1	NFIC	-0.3165	0
AKT1S1	NFKB2	0.9964	1
AKT1S1	NOTCH1	0.4074	0
AKT1S1	PRKAA2	-0.5942	1
AKT1S1	RELA	0.4812	0
AKT1S1	SMAD1	-0.6754	1
AKT1S1	SMAD4	0.1887	0
AKT1S1	STAT1	-0.0254	0
AKT1S1	STAT2	-0.0458	0
AKT1S1	STK3	-0.3383	0
AKT1S1	STK4	0.4512	0
AKT1S1	TNFRSF1A	-0.3748	0
ARAF	ABCB5	-0.4693	0
ARAF	AKT1S1	0.7390	1
ARAF	APC	0.3138	0

GENE1	GENE2	R	Positive
ARAF	ARNT	-0.2843	0
ARAF	AURKA	-0.9051	1
ARAF	BAX	0.0456	0
ARAF	BCL2	-0.1880	0
ARAF	BECN1	-0.8451	1
ARAF	BRAF	-0.6477	1
ARAF	CBLB	-0.8928	1
ARAF	CBLC	0.0666	0
ARAF	CCND2	-0.5331	1
ARAF	CCNE1	0.4380	0
ARAF	CD44	-0.0023	0
ARAF	CDC42	0.7131	1
ARAF	CDK4	0.6333	1
ARAF	CDK6	0.2754	0
ARAF	CDKN2A	0.5148	1
ARAF	CDKN2B	-0.1997	0
ARAF	CDKN2C	-0.2480	0
ARAF	CTNNB1	0.4853	0
ARAF	DACH1	0.1551	0
ARAF	DVL1	-0.1277	0
ARAF	E2F1	-0.3027	0
ARAF	EGFR	0.5361	1
ARAF	EPHA2	0.5843	1
ARAF	ERBB2	0.3191	0
ARAF	FGFR4	-0.3753	0
ARAF	FOXO1	0.1023	0
ARAF	FZR1	0.2139	0
ARAF	GLIS2	-0.8683	1
ARAF	GRM1	-0.0567	0
ARAF	GSK3B	-0.2473	0
ARAF	HGF	-0.2329	0
ARAF	HIF1A	-0.5536	1
ARAF	HRAS	-0.0689	0
ARAF	IGF1R	0.4277	0
ARAF	JAK1	-0.2082	0
ARAF	JAK2	0.2458	0
ARAF	KAT2A	-0.5003	1
ARAF	KDELR2	-0.3471	0
ARAF	KEAP1	-0.7243	1
ARAF	KRAS	-0.5112	1
ARAF	LATS2	0.5727	1
ARAF	MAP2K1	-0.1221	0
ARAF	MAP2K3	-0.0596	0
ARAF	MAP2K4	-0.0386	0
ARAF	MAP2K5	0.3020	0
ARAF	MAP2K6	-0.3341	0
ARAF	MAP3K5	-0.7977	1
ARAF	MAPK1	0.7874	1
ARAF	MAPK14	-0.6486	1

GENE1	GENE2	R	Positive
ARAF	MAPK3	-0.5314	1
ARAF	MAPK8	0.6329	1
ARAF	MAPK9	-0.5843	1
ARAF	MDM2	0.3675	0
ARAF	MDM4	-0.2872	0
ARAF	MET	-0.8183	1
ARAF	MSH2	-0.6452	1
ARAF	MTOR	-0.0485	0
ARAF	NF1	0.0943	0
ARAF	NF2	-0.9826	1
ARAF	NFIC	0.2818	0
ARAF	NFKB2	0.6810	1
ARAF	NOTCH1	0.1668	0
ARAF	PIK3CA	-0.4399	0
ARAF	PIK3R1	-0.2315	0
ARAF	PRKAA2	-0.6860	1
ARAF	PTEN	-0.5014	1
ARAF	RASSF1	-0.4634	0
ARAF	RB1	-0.3345	0
ARAF	RELA	0.8014	1
ARAF	ROS1	-0.1802	0
ARAF	SFN	0.5276	1
ARAF	SMAD1	-0.8510	1
ARAF	SMAD2	-0.5126	1
ARAF	SMAD4	-0.3078	0
ARAF	SMARCA4	-0.4543	0
ARAF	SOX3	0.8477	1
ARAF	SOX4	0.0831	0
ARAF	STAT1	-0.0251	0
ARAF	STAT2	0.3008	0
ARAF	STK11	-0.2822	0
ARAF	STK3	-0.1089	0
ARAF	STK4	0.7433	1
ARAF	TCF3	-0.1009	0
ARAF	TEAD2	0.4391	0
ARAF	TERT	-0.5553	1
ARAF	TNFRSF1A	-0.3949	0
ARAF	TP53	0.1984	0
ARAF	TSC1	-0.3908	0
ARAF	WHSC1L1	-0.4598	0
ARAF	WT1	0.4274	0
ARAF	YAP1	-0.6699	1
ARAF	YWHAZ	0.9063	1
ARNT	ABCB5	-0.1411	0
ARNT	AKT1S1	0.3531	0
ARNT	APC	-0.7429	1
ARNT	BAX	-0.6087	1
ARNT	BCL2	-0.1041	0
ARNT	BECN1	0.1444	0

GENE1	GENE2	R	Positive
ARNT	CCNE1	-0.2890	0
ARNT	CD44	0.7335	1
ARNT	CDC42	0.2731	0
ARNT	CDKN2C	0.0013	0
ARNT	DACH1	0.2024	0
ARNT	DVL1	-0.3686	0
ARNT	E2F1	0.3927	0
ARNT	FZR1	0.3019	0
ARNT	GRM1	0.1711	0
ARNT	GSK3B	-0.2796	0
ARNT	HGF	0.4474	0
ARNT	HIF1A	0.0203	0
ARNT	IGF1R	0.3962	0
ARNT	JAK1	-0.5605	1
ARNT	JAK2	0.0511	0
ARNT	KAT2A	0.3908	0
ARNT	KDELR2	0.4959	0
ARNT	KEAP1	0.7536	1
ARNT	MAP2K1	0.0022	0
ARNT	MAP2K4	-0.3145	0
ARNT	MAP2K6	-0.0879	0
ARNT	MAP3K5	0.2878	0
ARNT	MAPK1	0.0604	0
ARNT	MAPK14	0.1888	0
ARNT	MAPK3	0.0909	0
ARNT	MAPK8	0.2019	0
ARNT	MAPK9	0.5202	1
ARNT	MDM4	-0.3459	0
ARNT	MTOR	-0.6771	1
ARNT	NFIC	-0.5605	1
ARNT	NFKB2	0.4202	0
ARNT	NOTCH1	0.1878	0
ARNT	PRKAA2	0.4225	0
ARNT	RELA	-0.5744	1
ARNT	SFN	-0.0763	0
ARNT	SMAD1	0.4059	0
ARNT	SMAD4	0.5254	1
ARNT	SOX3	-0.4061	0
ARNT	SOX4	-0.3758	0
ARNT	STAT1	0.0860	0
ARNT	STAT2	-0.8155	1
ARNT	STK3	0.0182	0
ARNT	STK4	-0.1383	0
ARNT	TEAD2	-0.1581	0
ARNT	TERT	-0.4414	0
ARNT	TNFRSF1A	-0.3553	0
ARNT	YWHAZ	-0.0846	0
AURKA	ABCB5	0.7042	1
AURKA	AKT1S1	-0.6755	1

GENE1	GENE2	R	Positive
AURKA	APC	-0.1239	0
AURKA	ARNT	0.1695	0
AURKA	BAX	0.0586	0
AURKA	BCL2	0.5615	1
AURKA	BECN1	0.8970	1
AURKA	BRAF	0.3879	0
AURKA	CBLB	0.8339	1
AURKA	CBLC	-0.4834	0
AURKA	CCND2	0.3324	0
AURKA	CCNE1	-0.0666	0
AURKA	CD44	0.1024	0
AURKA	CDC42	-0.8096	1
AURKA	CDK4	-0.8092	1
AURKA	CDK6	-0.4573	0
AURKA	CDKN2A	-0.1882	0
AURKA	CDKN2B	0.1656	0
AURKA	CDKN2C	-0.1271	0
AURKA	CTNNB1	-0.4413	0
AURKA	DACH1	-0.3972	0
AURKA	DVL1	0.3884	0
AURKA	E2F1	0.4639	0
AURKA	EGFR	-0.1364	0
AURKA	EPHA2	-0.2291	0
AURKA	ERBB2	-0.0685	0
AURKA	FGFR4	0.6684	1
AURKA	FOXO1	-0.4700	0
AURKA	FZR1	-0.3181	0
AURKA	GLIS2	0.9661	1
AURKA	GRM1	-0.3126	0
AURKA	GSK3B	0.5161	1
AURKA	HGF	-0.0051	0
AURKA	HIF1A	0.7684	1
AURKA	HRAS	0.3342	0
AURKA	IGF1R	-0.5015	1
AURKA	JAK1	0.0041	0
AURKA	JAK2	-0.3545	0
AURKA	KAT2A	0.1469	0
AURKA	KDELR2	0.6155	1
AURKA	KEAP1	0.5759	1
AURKA	KRAS	0.8156	1
AURKA	LATS2	-0.6764	1
AURKA	MAP2K1	0.0661	0
AURKA	MAP2K3	-0.1041	0
AURKA	MAP2K4	0.0562	0
AURKA	MAP2K5	-0.5648	1
AURKA	MAP2K6	0.5131	1
AURKA	MAP3K5	0.4792	0
AURKA	MAPK1	-0.5971	1
AURKA	MAPK14	0.7370	1

GENE1	GENE2	R	Positive
AURKA	MAPK3	0.7786	1
AURKA	MAPK8	-0.6669	1
AURKA	MAPK9	0.6846	1
AURKA	MDM2	-0.5033	1
AURKA	MDM4	0.0209	0
AURKA	MET	0.7433	1
AURKA	MSH2	0.7579	1
AURKA	MTOR	0.0689	0
AURKA	NF1	-0.3263	0
AURKA	NF2	0.8930	1
AURKA	NFIC	-0.1717	0
AURKA	NFKB2	-0.6235	1
AURKA	NOTCH1	0.0590	0
AURKA	PIK3CA	0.6994	1
AURKA	PIK3R1	0.2040	0
AURKA	PRKAA2	0.5096	1
AURKA	PTEN	0.2024	0
AURKA	RASSF1	0.3501	0
AURKA	RB1	0.5726	1
AURKA	RELA	-0.6104	1
AURKA	ROS1	-0.0388	0
AURKA	SFN	-0.6966	1
AURKA	SMAD1	0.7744	1
AURKA	SMAD2	0.6936	1
AURKA	SMAD4	0.3195	0
AURKA	SMARCA4	0.4674	0
AURKA	SOX3	-0.6959	1
AURKA	SOX4	0.3126	0
AURKA	STAT1	0.3732	0
AURKA	STAT2	-0.0532	0
AURKA	STK11	0.2359	0
AURKA	STK3	0.0645	0
AURKA	STK4	-0.7493	1
AURKA	TCF3	-0.1111	0
AURKA	TEAD2	-0.2038	0
AURKA	TERT	0.5300	1
AURKA	TNFRSF1A	0.3577	0
AURKA	TP53	0.0124	0
AURKA	TSC1	0.5769	1
AURKA	WHSC1L1	0.7744	1
AURKA	WT1	-0.2612	0
AURKA	YAP1	0.7574	1
AURKA	YWHAZ	-0.7818	1
BECN1	ABCB5	0.5939	1
BECN1	AKT1S1	-0.6781	1
BECN1	APC	0.0776	0
BECN1	BAX	0.0532	0
BECN1	BCL2	0.5900	1
BECN1	CCNE1	0.0534	0

GENE1	GENE2	R	Positive
BECN1	CD44	0.2407	0
BECN1	CDC42	-0.8885	1
BECN1	CDKN2C	0.0224	0
BECN1	DACH1	-0.4285	0
BECN1	DVL1	0.1906	0
BECN1	E2F1	0.4073	0
BECN1	FZR1	-0.5943	1
BECN1	GRM1	-0.1600	0
BECN1	GSK3B	0.5479	1
BECN1	HGF	0.0984	0
BECN1	HIF1A	0.7483	1
BECN1	IGF1R	-0.5130	1
BECN1	JAK1	0.1844	0
BECN1	JAK2	-0.6682	1
BECN1	KAT2A	0.1401	0
BECN1	KEAP1	0.6539	1
BECN1	MAP2K1	-0.2789	0
BECN1	MAP2K4	0.2439	0
BECN1	MAP2K6	0.5852	1
BECN1	MAP3K5	0.5467	1
BECN1	MAPK1	-0.4127	0
BECN1	MAPK14	0.9341	1
BECN1	MAPK3	0.8605	1
BECN1	MAPK8	-0.8943	1
BECN1	MAPK9	0.5936	1
BECN1	MDM4	0.0574	0
BECN1	MTOR	-0.1377	0
BECN1	NFIC	0.0115	0
BECN1	NFKB2	-0.6428	1
BECN1	NOTCH1	-0.3332	0
BECN1	PRKAA2	0.5618	1
BECN1	RELA	-0.4574	0
BECN1	SFN	-0.7871	1
BECN1	SMAD1	0.7810	1
BECN1	SMAD4	0.5340	1
BECN1	SOX3	-0.7479	1
BECN1	SOX4	0.0641	0
BECN1	STAT1	0.3415	0
BECN1	STAT2	-0.0998	0
BECN1	STK3	-0.1210	0
BECN1	STK4	-0.8622	1
BECN1	TEAD2	-0.4735	0
BECN1	TERT	0.2786	0
BECN1	TNFRSF1A	0.1465	0
BECN1	YWHAZ	-0.8801	1
BRAF	ABCB5	0.0095	0
BRAF	AKT1S1	-0.7859	1
BRAF	APC	0.0944	0
BRAF	ARNT	-0.2513	0

GENE1	GENE2	R	Positive
BRAF	BAX	0.1004	0
BRAF	BCL2	-0.2668	0
BRAF	BECN1	0.4920	0
BRAF	CBLB	0.7160	1
BRAF	CBLC	0.4374	0
BRAF	CCND2	0.0875	0
BRAF	CCNE1	-0.5544	1
BRAF	CD44	-0.4842	0
BRAF	CDC42	-0.5547	1
BRAF	CDK4	-0.2509	0
BRAF	CDK6	0.0287	0
BRAF	CDKN2A	-0.7679	1
BRAF	CDKN2B	0.1878	0
BRAF	CDKN2C	0.7636	1
BRAF	CTNNB1	-0.2119	0
BRAF	DACH1	0.1547	0
BRAF	DVL1	-0.1740	0
BRAF	E2F1	-0.1585	0
BRAF	EGFR	-0.7473	1
BRAF	EPHA2	-0.9297	1
BRAF	FGFR4	-0.1346	0
BRAF	FOXO1	0.3455	0
BRAF	FZR1	-0.2428	0
BRAF	GLIS2	0.3729	0
BRAF	GRM1	0.4119	0
BRAF	GSK3B	-0.0598	0
BRAF	HGF	0.1558	0
BRAF	HIF1A	0.0409	0
BRAF	HRAS	-0.3091	0
BRAF	IGF1R	-0.3683	0
BRAF	JAK1	0.7700	1
BRAF	JAK2	-0.2291	0
BRAF	KAT2A	0.6319	1
BRAF	KDELR2	-0.4749	0
BRAF	KEAP1	0.3236	0
BRAF	KRAS	-0.0734	0
BRAF	LATS2	-0.2236	0
BRAF	MAP2K1	-0.0374	0
BRAF	MAP2K3	0.3653	0
BRAF	MAP2K4	0.1626	0
BRAF	MAP2K5	-0.0361	0
BRAF	MAP2K6	0.0029	0
BRAF	MAP3K5	0.8181	1
BRAF	MAPK1	-0.7733	1
BRAF	MAPK14	0.2915	0
BRAF	MAPK3	0.0265	0
BRAF	MAPK8	-0.6262	1
BRAF	MAPK9	0.0138	0
BRAF	MDM2	-0.1621	0

GENE1	GENE2	R	Positive
BRAF	MDM4	0.7064	1
BRAF	MSH2	0.0784	0
BRAF	MTOR	0.2788	0
BRAF	NF1	0.4690	0
BRAF	NF2	0.5717	1
BRAF	NFIC	-0.0266	0
BRAF	NFKB2	-0.7804	1
BRAF	NOTCH1	-0.6576	1
BRAF	PIK3CA	-0.2489	0
BRAF	PIK3R1	0.3732	0
BRAF	PRKAA2	0.4122	0
BRAF	PTEN	0.5403	1
BRAF	RASSF1	0.4058	0
BRAF	RB1	0.0094	0
BRAF	RELA	-0.3834	0
BRAF	ROS1	0.3721	0
BRAF	SFN	-0.2124	0
BRAF	SMAD1	0.4142	0
BRAF	SMAD2	0.3197	0
BRAF	SMAD4	0.0388	0
BRAF	SMARCA4	0.2688	0
BRAF	SOX3	-0.5292	1
BRAF	SOX4	-0.4321	0
BRAF	STAT1	-0.5477	1
BRAF	STAT2	-0.0148	0
BRAF	STK11	0.1780	0
BRAF	STK3	-0.0507	0
BRAF	STK4	-0.5443	1
BRAF	TCF3	0.4596	0
BRAF	TEAD2	-0.5610	1
BRAF	TERT	0.6100	1
BRAF	TNFRSF1A	0.5351	1
BRAF	TP53	-0.2640	0
BRAF	TSC1	-0.2544	0
BRAF	WHSC1L1	-0.1847	0
BRAF	WT1	-0.3973	0
BRAF	YAP1	0.5022	1
BRAF	YWHAZ	-0.8171	1
CBLB	ABCB5	0.6014	1
CBLB	AKT1S1	-0.8887	1
CBLB	APC	-0.0384	0
CBLB	ARNT	0.0121	0
CBLB	BAX	0.2721	0
CBLB	BCL2	0.3113	0
CBLB	BECN1	0.9179	1
CBLB	CCND2	0.1588	0
CBLB	CCNE1	-0.2454	0
CBLB	CD44	-0.0918	0
CBLB	CDC42	-0.9144	1

GENE1	GENE2	R	Positive
CBLB	CDK4	-0.7213	1
CBLB	CDK6	-0.4303	0
CBLB	CDKN2A	-0.5796	1
CBLB	CDKN2B	0.3916	0
CBLB	CDKN2C	0.1954	0
CBLB	DACH1	-0.4308	0
CBLB	DVL1	0.2603	0
CBLB	E2F1	0.0669	0
CBLB	EPHA2	-0.5391	1
CBLB	FGFR4	0.2067	0
CBLB	FZR1	-0.6138	1
CBLB	GLIS2	0.8456	1
CBLB	GRM1	0.1458	0
CBLB	GSK3B	0.5203	1
CBLB	HGF	0.3079	0
CBLB	HIF1A	0.6985	1
CBLB	IGF1R	-0.6968	1
CBLB	JAK1	0.5004	1
CBLB	JAK2	-0.4391	0
CBLB	KAT2A	0.2456	0
CBLB	KDELR2	0.1994	0
CBLB	KEAP1	0.5346	1
CBLB	LATS2	-0.3999	0
CBLB	MAP2K1	-0.0250	0
CBLB	MAP2K3	-0.1047	0
CBLB	MAP2K4	0.4224	0
CBLB	MAP2K5	-0.1928	0
CBLB	MAP2K6	0.6007	1
CBLB	MAP3K5	0.7568	1
CBLB	MAPK1	-0.6488	1
CBLB	MAPK14	0.8196	1
CBLB	MAPK3	0.6605	1
CBLB	MAPK8	-0.8489	1
CBLB	MAPK9	0.3197	0
CBLB	MDM4	0.4452	0
CBLB	MSH2	0.7291	1
CBLB	MTOR	0.1557	0
CBLB	NF1	0.2314	0
CBLB	NF2	0.8200	1
CBLB	NFIC	0.1434	0
CBLB	NFKB2	-0.8621	1
CBLB	NOTCH1	-0.4659	0
CBLB	PIK3R1	0.5097	1
CBLB	PRKAA2	0.7293	1
CBLB	PTEN	0.4435	0
CBLB	RASSF1	0.1127	0
CBLB	RB1	0.2759	0
CBLB	RELA	-0.5974	1
CBLB	ROS1	0.2739	0

GENE1	GENE2	R	Positive
CBLB	SFN	-0.5195	1
CBLB	SMAD1	0.8589	1
CBLB	SMAD2	0.7794	1
CBLB	SMAD4	0.2400	0
CBLB	SMARCA4	0.7306	1
CBLB	SOX3	-0.8649	1
CBLB	SOX4	-0.0983	0
CBLB	STAT1	0.1550	0
CBLB	STAT2	-0.1839	0
CBLB	STK11	0.5132	1
CBLB	STK3	0.1421	0
CBLB	STK4	-0.7117	1
CBLB	TEAD2	-0.6630	1
CBLB	TERT	0.4648	0
CBLB	TNFRSF1A	0.2038	0
CBLB	TP53	0.1187	0
CBLB	TSC1	0.4606	0
CBLB	WT1	-0.3132	0
CBLB	YWHAZ	-0.9164	1
CBLC	ABCB5	-0.6867	1
CBLC	AKT1S1	0.0553	0
CBLC	APC	-0.3151	0
CBLC	ARNT	0.1786	0
CBLC	BAX	-0.2268	0
CBLC	BCL2	-0.9053	1
CBLC	BECN1	-0.3349	0
CBLC	CBLB	-0.0960	0
CBLC	CCND2	0.2778	0
CBLC	CCNE1	-0.7133	1
CBLC	CD44	-0.2106	0
CBLC	CDC42	0.4039	0
CBLC	CDK4	0.5589	1
CBLC	CDK6	0.5177	1
CBLC	CDKN2A	-0.6545	1
CBLC	CDKN2B	0.0865	0
CBLC	CDKN2C	0.8190	1
CBLC	CTNNB1	0.0765	0
CBLC	DACH1	0.5942	1
CBLC	DVL1	-0.6658	1
CBLC	E2F1	-0.4634	0
CBLC	EGFR	-0.7773	1
CBLC	EPHA2	-0.6626	1
CBLC	FGFR4	-0.8092	1
CBLC	FOXO1	0.9031	1
CBLC	FZR1	0.2532	0
CBLC	GLIS2	-0.4635	0
CBLC	GRM1	0.8572	1
CBLC	GSK3B	-0.6804	1
CBLC	HGF	0.4976	0

GENE1	GENE2	R	Positive
CBLC	HIF1A	-0.6495	1
CBLC	HRAS	-0.5983	1
CBLC	IGF1R	0.2846	0
CBLC	JAK1	0.4427	0
CBLC	JAK2	0.2661	0
CBLC	KAT2A	0.6735	1
CBLC	KDELR2	-0.7428	1
CBLC	KEAP1	0.1532	0
CBLC	KRAS	-0.8548	1
CBLC	LATS2	0.3991	0
CBLC	MAP2K1	0.0390	0
CBLC	MAP2K3	0.3761	0
CBLC	MAP2K4	-0.0205	0
CBLC	MAP2K5	0.7327	1
CBLC	MAP2K6	-0.4921	0
CBLC	MAP3K5	0.5234	1
CBLC	MAPK1	-0.1921	0
CBLC	MAPK14	-0.3499	0
CBLC	MAPK3	-0.7014	1
CBLC	MAPK8	0.2143	0
CBLC	MAPK9	-0.4067	0
CBLC	MDM2	0.4011	0
CBLC	MDM4	0.5400	1
CBLC	MSH2	-0.4401	0
CBLC	MTOR	-0.0917	0
CBLC	NF1	0.6230	1
CBLC	NF2	-0.0748	0
CBLC	NFIC	-0.1453	0
CBLC	NFKB2	0.0506	0
CBLC	NOTCH1	-0.5372	1
CBLC	PIK3CA	-0.7710	1
CBLC	PIK3R1	0.0030	0
CBLC	PRKAA2	0.2239	0
CBLC	PTEN	0.5877	1
CBLC	RASSF1	0.0898	0
CBLC	RB1	-0.6354	1
CBLC	RELA	-0.1924	0
CBLC	ROS1	0.5143	1
CBLC	SFN	0.5183	1
CBLC	SMAD1	-0.0580	0
CBLC	SMAD2	-0.5298	1
CBLC	SMAD4	-0.0778	0
CBLC	SMARCA4	-0.1293	0
CBLC	SOX3	-0.1231	0
CBLC	SOX4	-0.9259	1
CBLC	STAT1	-0.8131	1
CBLC	STAT2	-0.4912	0
CBLC	STK11	0.0872	0
CBLC	STK3	0.0412	0
CBLC	STK4	0.1994	0
CBLC	TCF3	0.5114	1
CBLC	TEAD2	-0.4632	0
CBLC	TERT	-0.1332	0
CBLC	TNFRSF1A	-0.0549	0
CBLC	TP53	-0.4265	0
CBLC	TSC1	-0.5230	1
CBLC	WHSC1L1	-0.8624	1
CBLC	WT1	-0.2207	0
CBLC	YAP1	-0.3620	0
CBLC	YWHAZ	-0.0536	0
CCND2	ABCB5	-0.0567	0
CCND2	AKT1S1	0.0052	0
CCND2	APC	-0.8983	1
CCND2	ARNT	0.7944	1
CCND2	BAX	-0.4587	0
CCND2	BCL2	-0.3623	0
CCND2	BECN1	0.1165	0
CCND2	CCNE1	-0.7084	1
CCND2	CD44	0.2154	0
CCND2	CDC42	0.1769	0
CCND2	CDK4	0.0938	0
CCND2	CDK6	0.0853	0
CCND2	CDKN2C	0.2002	0
CCND2	DACH1	0.3207	0
CCND2	DVL1	-0.1957	0
CCND2	E2F1	0.2306	0
CCND2	FZR1	0.5213	1
CCND2	GLIS2	0.2422	0
CCND2	GRM1	0.2584	0
CCND2	GSK3B	-0.3711	0
CCND2	HGF	0.4113	0
CCND2	HIF1A	-0.0213	0
CCND2	IGF1R	0.2200	0
CCND2	JAK1	-0.3229	0
CCND2	JAK2	0.4092	0
CCND2	KAT2A	0.6439	1
CCND2	KDELR2	0.3513	0
CCND2	KEAP1	0.6539	1
CCND2	MAP2K1	0.4499	0
CCND2	MAP2K3	0.0748	0
CCND2	MAP2K4	-0.3980	0
CCND2	MAP2K6	-0.2042	0
CCND2	MAP3K5	0.5386	1
CCND2	MAPK1	-0.4901	0
CCND2	MAPK14	-0.0301	0
CCND2	MAPK3	-0.1219	0
CCND2	MAPK8	0.2613	0
CCND2	MAPK9	0.4576	0

GENE1	GENE2	R	Positive
CCND2	MDM4	0.0330	0
CCND2	MTOR	-0.1944	0
CCND2	NFIC	-0.6896	1
CCND2	NFKB2	0.0818	0
CCND2	NOTCH1	0.3168	0
CCND2	PRKAA2	0.4981	0
CCND2	RELA	-0.8489	1
CCND2	SFN	0.0790	0
CCND2	SMAD1	0.4899	0
CCND2	SMAD4	0.1432	0
CCND2	SOX3	-0.5021	1
CCND2	SOX4	-0.3052	0
CCND2	STAT1	-0.1929	0
CCND2	STAT2	-0.7190	1
CCND2	STK3	0.2761	0
CCND2	STK4	-0.1157	0
CCND2	TEAD2	-0.0959	0
CCND2	TERT	0.1430	0
CCND2	TNFRSF1A	0.1250	0
CCND2	YWHAZ	-0.2431	0
CCNE1	ABCB5	0.1956	0
CCNE1	AKT1S1	0.3333	0
CCNE1	APC	0.6993	1
CCNE1	BAX	0.0709	0
CCNE1	BCL2	0.7818	1
CCNE1	CD44	0.4036	0
CCNE1	CDC42	-0.1011	0
CCNE1	CDKN2C	-0.6192	1
CCNE1	DACH1	-0.3778	0
CCNE1	DVL1	0.2116	0
CCNE1	E2F1	0.3626	0
CCNE1	GRM1	-0.7185	1
CCNE1	GSK3B	0.4396	0
CCNE1	HGF	-0.5602	1
CCNE1	HIF1A	0.2401	0
CCNE1	IGF1R	0.0465	0
CCNE1	JAK1	-0.3699	0
CCNE1	JAK2	-0.5493	1
CCNE1	KAT2A	-0.7541	1
CCNE1	KEAP1	-0.3051	0
CCNE1	MAP2K1	-0.5712	1
CCNE1	MAP2K4	0.0582	0
CCNE1	MAP2K6	0.2561	0
CCNE1	MAP3K5	-0.7709	1
CCNE1	MAPK1	0.7328	1
CCNE1	MAPK14	0.2282	0
CCNE1	MAPK3	0.4814	0
CCNE1	MAPK8	-0.1824	0
CCNE1	MAPK9	0.1238	0

GENE1	GENE2	R	Positive
CCNE1	MDM4	-0.6760	1
CCNE1	MTOR	-0.2653	0
CCNE1	NFIC	0.3235	0
CCNE1	NFKB2	0.2983	0
CCNE1	NOTCH1	0.1459	0
CCNE1	PRKAA2	-0.5455	1
CCNE1	RELA	0.7393	1
CCNE1	SFN	-0.4357	0
CCNE1	SMAD1	-0.3840	0
CCNE1	SMAD4	0.2836	0
CCNE1	SOX3	0.4917	0
CCNE1	SOX4	0.6032	1
CCNE1	STAT1	0.6176	1
CCNE1	STAT2	0.5832	1
CCNE1	STK3	-0.4458	0
CCNE1	STK4	-0.0711	0
CCNE1	TEAD2	0.4028	0
CCNE1	TERT	-0.3981	0
CCNE1	TNFRSF1A	-0.2635	0
CCNE1	YWHAZ	0.3049	0
CD44	ABCB5	-0.1048	0
CD44	AKT1S1	0.5310	1
CD44	APC	-0.1273	0
CD44	BAX	-0.6035	1
CD44	BCL2	0.4079	0
CD44	CDC42	0.1489	0
CD44	CDKN2C	-0.2556	0
CD44	DACH1	0.0176	0
CD44	DVL1	-0.3605	0
CD44	E2F1	0.6483	1
CD44	GRM1	-0.2711	0
CD44	GSK3B	-0.0298	0
CD44	HGF	0.0323	0
CD44	HIF1A	0.1188	0
CD44	IGF1R	0.4478	0
CD44	JAK1	-0.6895	1
CD44	JAK2	-0.4787	0
CD44	KAT2A	-0.0376	0
CD44	KEAP1	0.5912	1
CD44	MAP2K1	-0.5573	1
CD44	MAP2K4	-0.2630	0
CD44	MAP2K6	0.0379	0
CD44	MAP3K5	-0.1665	0
CD44	MAPK1	0.5564	1
CD44	MAPK14	0.4008	0
CD44	MAPK3	0.4196	0
CD44	MAPK8	-0.0615	0
CD44	MAPK9	0.5952	1
CD44	MDM4	-0.7595	1

GENE1	GENE2	R	Positive
CD44	MTOR	-0.9192	1
CD44	NFIC	-0.3241	0
CD44	NFKB2	0.5676	1
CD44	NOTCH1	0.0922	0
CD44	PRKAA2	0.0274	0
CD44	RELA	0.0026	0
CD44	SFN	-0.4552	0
CD44	SMAD1	0.1158	0
CD44	SMAD4	0.8191	1
CD44	SOX3	-0.0736	0
CD44	SOX4	-0.0797	0
CD44	STAT1	0.3891	0
CD44	STAT2	-0.3672	0
CD44	STK3	-0.4344	0
CD44	STK4	-0.3034	0
CD44	TERT	-0.7230	1
CD44	TNFRSF1A	-0.4923	0
CD44	YWHAZ	0.0124	0
CDC42	APC	-0.2662	0
CDC42	BAX	-0.4622	0
CDC42	BCL2	-0.5827	1
CDC42	DVL1	-0.4887	0
CDC42	GSK3B	-0.7497	1
CDC42	JAK1	-0.4672	0
CDC42	JAK2	0.5356	1
CDC42	MAP2K1	0.0982	0
CDC42	MAP2K4	-0.5081	1
CDC42	MAPK1	0.4690	0
CDC42	MAPK3	-0.7929	1
CDC42	MAPK8	0.8937	1
CDC42	MAPK9	-0.2512	0
CDC42	MTOR	-0.2782	0
CDC42	NFKB2	0.8653	1
CDC42	NOTCH1	0.3668	0
CDC42	PRKAA2	-0.4946	0
CDC42	SMAD4	-0.1491	0
CDC42	STAT1	-0.3780	0
CDC42	STAT2	-0.1534	0
CDK4	ABCB5	-0.5453	1
CDK4	AKT1S1	0.4518	0
CDK4	APC	-0.3172	0
CDK4	ARNT	-0.0722	0
CDK4	BAX	0.0421	0
CDK4	BCL2	-0.8106	1
CDK4	BECN1	-0.9281	1
CDK4	CCNE1	-0.3978	0
CDK4	CD44	-0.4048	0
CDK4	CDC42	0.8044	1
CDK4	CDKN2C	0.1588	0

GENE1	GENE2	R	Positive
CDK4	DACH1	0.4257	0
CDK4	DVL1	-0.1733	0
CDK4	E2F1	-0.6035	1
CDK4	FZR1	0.5726	1
CDK4	GLIS2	-0.9194	1
CDK4	GRM1	0.4607	0
CDK4	GSK3B	-0.5703	1
CDK4	HGF	0.1861	0
CDK4	HIF1A	-0.7082	1
CDK4	IGF1R	0.3523	0
CDK4	JAK1	0.0384	0
CDK4	JAK2	0.8197	1
CDK4	KAT2A	0.0664	0
CDK4	KDELR2	-0.5228	1
CDK4	KEAP1	-0.5447	1
CDK4	MAP2K1	0.4935	0
CDK4	MAP2K3	-0.0577	0
CDK4	MAP2K4	-0.1167	0
CDK4	MAP2K6	-0.5285	1
CDK4	MAP3K5	-0.2179	0
CDK4	MAPK1	0.1295	0
CDK4	MAPK14	-0.9020	1
CDK4	MAPK3	-0.9366	1
CDK4	MAPK8	0.8649	1
CDK4	MAPK9	-0.6782	1
CDK4	MDM4	0.2606	0
CDK4	MTOR	0.2831	0
CDK4	NFIC	-0.0034	0
CDK4	NFKB2	0.4258	0
CDK4	NOTCH1	0.2050	0
CDK4	PRKAA2	-0.2594	0
CDK4	RELA	0.1549	0
CDK4	SFN	0.9283	1
CDK4	SMAD1	-0.5377	1
CDK4	SMAD4	-0.6546	1
CDK4	SOX3	0.4702	0
CDK4	SOX4	-0.2794	0
CDK4	STAT1	-0.4775	0
CDK4	STAT2	-0.1365	0
CDK4	STK3	0.3563	0
CDK4	STK4	0.8666	1
CDK4	TEAD2	0.2133	0
CDK4	TERT	-0.1285	0
CDK4	TNFRSF1A	-0.1159	0
CDK4	YWHAZ	0.7124	1
CDK6	ABCB5	-0.9291	1
CDK6	AKT1S1	0.5804	1
CDK6	APC	0.1421	0
CDK6	ARNT	0.2590	0

GENE1	GENE2	R	Positive
CDK6	BAX	-0.8425	1
CDK6	BCL2	-0.3909	0
CDK6	BECN1	-0.3092	0
CDK6	CCNE1	-0.0476	0
CDK6	CD44	0.3485	0
CDK6	CDC42	0.5796	1
CDK6	CDK4	0.2094	0
CDK6	CDKN2C	0.6131	1
CDK6	DACH1	0.8828	1
CDK6	DVL1	-0.9636	1
CDK6	E2F1	0.3700	0
CDK6	FZR1	0.5246	1
CDK6	GLIS2	-0.3310	0
CDK6	GRM1	0.0443	0
CDK6	GSK3B	-0.8554	1
CDK6	HGF	-0.2896	0
CDK6	HIF1A	-0.8136	1
CDK6	IGF1R	0.8841	1
CDK6	JAK1	-0.2498	0
CDK6	JAK2	-0.2158	0
CDK6	KAT2A	0.5617	1
CDK6	KDELR2	-0.4368	0
CDK6	KEAP1	0.2847	0
CDK6	MAP2K1	-0.5821	1
CDK6	MAP2K3	0.8792	1
CDK6	MAP2K4	-0.6774	1
CDK6	MAP2K6	-0.8429	1
CDK6	MAP3K5	-0.0461	0
CDK6	MAPK1	0.2719	0
CDK6	MAPK14	-0.3457	0
CDK6	MAPK3	-0.4652	0
CDK6	MAPK8	0.1757	0
CDK6	MAPK9	0.2040	0
CDK6	MDM4	-0.3528	0
CDK6	MTOR	-0.6722	1
CDK6	NFIC	-0.6012	1
CDK6	NFKB2	0.5869	1
CDK6	NOTCH1	-0.1887	0
CDK6	PRKAA2	-0.4664	0
CDK6	RELA	0.3131	0
CDK6	SFN	-0.0958	0
CDK6	SMAD1	-0.5252	1
CDK6	SMAD4	0.5236	1
CDK6	SOX3	0.4116	0
CDK6	SOX4	-0.4992	0
CDK6	STAT1	-0.6693	1
CDK6	STAT2	-0.0118	0
CDK6	STK3	-0.7623	1
CDK6	STK4	-0.1565	0

GENE1	GENE2	R	Positive
CDK6	TEAD2	0.2137	0
CDK6	TERT	-0.3703	0
CDK6	TNFRSF1A	0.1400	0
CDK6	YWHAZ	0.0620	0
CDKN2A	ABCB5	0.2595	0
CDKN2A	AKT1S1	0.4019	0
CDKN2A	APC	0.0762	0
CDKN2A	ARNT	-0.2342	0
CDKN2A	BAX	0.2362	0
CDKN2A	BCL2	0.3185	0
CDKN2A	BECN1	-0.4684	0
CDKN2A	CCND2	-0.2199	0
CDKN2A	CCNE1	0.4854	0
CDKN2A	CD44	-0.0713	0
CDKN2A	CDC42	0.3024	0
CDKN2A	CDK4	0.2584	0
CDKN2A	CDK6	-0.3563	0
CDKN2A	CDKN2C	-0.8035	1
CDKN2A	DACH1	-0.2593	0
CDKN2A	DVL1	0.5683	1
CDKN2A	E2F1	0.0423	0
CDKN2A	EPHA2	0.7624	1
CDKN2A	FGFR4	0.3768	0
CDKN2A	FZR1	0.2677	0
CDKN2A	GLIS2	-0.3036	0
CDKN2A	GRM1	-0.6053	1
CDKN2A	GSK3B	0.2298	0
CDKN2A	HGF	-0.4377	0
CDKN2A	HIF1A	0.0771	0
CDKN2A	IGF1R	0.0545	0
CDKN2A	JAK1	-0.5360	1
CDKN2A	JAK2	0.4208	0
CDKN2A	KAT2A	-0.7041	1
CDKN2A	KDELR2	0.4024	0
CDKN2A	KEAP1	-0.6457	1
CDKN2A	MAP2K1	0.3677	0
CDKN2A	MAP2K3	-0.4461	0
CDKN2A	MAP2K4	-0.1399	0
CDKN2A	MAP2K5	-0.5033	1
CDKN2A	MAP2K6	0.0469	0
CDKN2A	MAP3K5	-0.8302	1
CDKN2A	MAPK1	0.3738	0
CDKN2A	MAPK14	-0.4271	0
CDKN2A	MAPK3	-0.0444	0
CDKN2A	MAPK8	0.5576	1
CDKN2A	MAPK9	-0.1032	0
CDKN2A	MDM4	-0.4494	0
CDKN2A	MSH2	-0.0834	0
CDKN2A	MTOR	0.3046	0

GENE1	GENE2	R	Positive
CDKN2A	NF1	-0.6994	1
CDKN2A	NFIC	0.1139	0
CDKN2A	NFKB2	0.3862	0
CDKN2A	NOTCH1	0.8331	1
CDKN2A	PIK3R1	-0.0784	0
CDKN2A	PRKAA2	-0.5306	1
CDKN2A	PTEN	-0.9260	1
CDKN2A	RB1	0.0322	0
CDKN2A	RELA	0.3808	0
CDKN2A	SFN	0.2200	0
CDKN2A	SMAD1	-0.4506	0
CDKN2A	SMAD2	-0.2114	0
CDKN2A	SMAD4	-0.4573	0
CDKN2A	SMARCA4	-0.3199	0
CDKN2A	SOX3	0.5999	1
CDKN2A	SOX4	0.8179	1
CDKN2A	STAT1	0.5007	1
CDKN2A	STAT2	0.4434	0
CDKN2A	STK3	0.2263	0
CDKN2A	STK4	0.5435	1
CDKN2A	TEAD2	0.7685	1
CDKN2A	TERT	0.0030	0
CDKN2A	TNFRSF1A	-0.0420	0
CDKN2A	TP53	0.4113	0
CDKN2A	TSC1	-0.0283	0
CDKN2A	WT1	0.0136	0
CDKN2A	YWHAZ	0.7248	1
CDKN2B	ABCB5	-0.0639	0
CDKN2B	AKT1S1	-0.0355	0
CDKN2B	APC	0.2849	0
CDKN2B	ARNT	0.2735	0
CDKN2B	BAX	-0.2524	0
CDKN2B	BCL2	0.3374	0
CDKN2B	BECN1	0.5597	1
CDKN2B	CCND2	-0.1889	0
CDKN2B	CCNE1	0.3034	0
CDKN2B	CD44	0.6292	1
CDKN2B	CDC42	-0.3373	0
CDKN2B	CDK4	-0.6124	1
CDKN2B	CDK6	0.3025	0
CDKN2B	CDKN2A	-0.6406	1
CDKN2B	CDKN2C	0.2088	0
CDKN2B	DACH1	-0.1134	0
CDKN2B	DVL1	-0.4215	0
CDKN2B	E2F1	0.2940	0
CDKN2B	EPHA2	-0.0167	0
CDKN2B	FGFR4	-0.0749	0
CDKN2B	FZR1	-0.5731	1
CDKN2B	GLIS2	0.4081	0
CDKN2B	GRM1	0.0959	0
CDKN2B	GSK3B	0.1545	0
CDKN2B	HGF	0.1816	0
CDKN2B	HIF1A	0.2040	0
CDKN2B	IGF1R	0.0149	0
CDKN2B	JAK1	0.0978	0
CDKN2B	JAK2	-0.8622	1
CDKN2B	KAT2A	0.0881	0
CDKN2B	KDELR2	0.0285	0
CDKN2B	KEAP1	0.5655	1
CDKN2B	MAP2K1	-0.8239	1
CDKN2B	MAP2K3	0.2533	0
CDKN2B	MAP2K4	0.2218	0
CDKN2B	MAP2K5	0.4627	0
CDKN2B	MAP2K6	0.2524	0
CDKN2B	MAP3K5	0.2512	0
CDKN2B	MAPK1	0.3102	0
CDKN2B	MAPK14	0.7192	1
CDKN2B	MAPK3	0.5152	1
CDKN2B	MAPK8	-0.6572	1
CDKN2B	MAPK9	0.2831	0
CDKN2B	MDM4	-0.1852	0
CDKN2B	MSH2	0.2438	0
CDKN2B	MTOR	-0.6967	1
CDKN2B	NF1	0.5785	1
CDKN2B	NFIC	0.1145	0
CDKN2B	NFKB2	-0.0347	0
CDKN2B	NOTCH1	-0.6851	1
CDKN2B	PIK3R1	-0.1560	0
CDKN2B	PRKAA2	0.2561	0
CDKN2B	PTEN	0.6895	1
CDKN2B	RASSF1	-0.4286	0
CDKN2B	RB1	0.3856	0
CDKN2B	RELA	0.0764	0
CDKN2B	SFN	-0.5611	1
CDKN2B	SMAD1	0.2884	0
CDKN2B	SMAD2	0.5264	1
CDKN2B	SMAD4	0.8062	1
CDKN2B	SMARCA4	0.4082	0
CDKN2B	SOX3	-0.3411	0
CDKN2B	SOX4	-0.4191	0
CDKN2B	STAT1	0.1326	0
CDKN2B	STAT2	-0.2305	0
CDKN2B	STK3	-0.5115	1
CDKN2B	STK4	-0.5840	1
CDKN2B	TEAD2	-0.5454	1
CDKN2B	TERT	-0.5232	1
CDKN2B	TNFRSF1A	-0.3971	0
CDKN2B	TP53	-0.1109	0

GENE1	GENE2	R	Positive
CDKN2B	TSC1	0.5389	1
CDKN2B	WT1	0.6509	1
CDKN2B	YWHAZ	-0.4563	0
CDKN2C	ABCB5	-0.6274	1
CDKN2C	AKT1S1	-0.2087	0
CDKN2C	APC	-0.0015	0
CDKN2C	BAX	-0.3749	0
CDKN2C	BCL2	-0.6560	1
CDKN2C	CDC42	0.0683	0
CDKN2C	DACH1	0.6942	1
CDKN2C	DVL1	-0.7262	1
CDKN2C	E2F1	-0.0976	0
CDKN2C	GSK3B	-0.6528	1
CDKN2C	HGF	0.1125	0
CDKN2C	HIF1A	-0.5628	1
CDKN2C	IGF1R	0.2602	0
CDKN2C	JAK1	0.4993	0
CDKN2C	JAK2	-0.0928	0
CDKN2C	KEAP1	0.3427	0
CDKN2C	MAP2K1	-0.2130	0
CDKN2C	MAP2K4	-0.2299	0
CDKN2C	MAP2K6	-0.5547	1
CDKN2C	MAP3K5	0.6410	1
CDKN2C	MAPK1	-0.4463	0
CDKN2C	MAPK14	-0.1366	0
CDKN2C	MAPK3	-0.4533	0
CDKN2C	MAPK8	-0.1958	0
CDKN2C	MAPK9	-0.0451	0
CDKN2C	MDM4	0.4659	0
CDKN2C	MTOR	-0.1100	0
CDKN2C	NFIC	-0.3470	0
CDKN2C	NFKB2	-0.2034	0
CDKN2C	NOTCH1	-0.6152	1
CDKN2C	PRKAA2	0.1015	0
CDKN2C	RELA	-0.1690	0
CDKN2C	SFN	0.0281	0
CDKN2C	SMAD1	-0.0152	0
CDKN2C	SMAD4	0.1797	0
CDKN2C	SOX3	-0.1617	0
CDKN2C	SOX4	-0.7602	1
CDKN2C	STAT1	-0.9130	1
CDKN2C	STAT2	-0.1570	0
CDKN2C	STK3	-0.3257	0
CDKN2C	STK4	-0.3071	0
CDKN2C	TERT	0.2102	0
CDKN2C	TNFRSF1A	0.4102	0
CDKN2C	YWHAZ	-0.4686	0
CTNNB1	ABCB5	-0.6378	1
CTNNB1	AKT1S1	0.5927	1

GENE1	GENE2	R	Positive
CTNNB1	APC	0.6032	1
CTNNB1	ARNT	-0.0224	0
CTNNB1	BAX	-0.5592	1
CTNNB1	BCL2	0.1316	0
CTNNB1	BECN1	-0.1700	0
CTNNB1	CBLB	-0.4173	0
CTNNB1	CCND2	-0.4515	0
CTNNB1	CCNE1	0.5589	1
CTNNB1	CD44	0.4963	0
CTNNB1	CDC42	0.3347	0
CTNNB1	CDK4	-0.0960	0
CTNNB1	CDK6	0.7766	1
CTNNB1	CDKN2A	-0.1275	0
CTNNB1	CDKN2B	0.5519	1
CTNNB1	CDKN2C	0.2012	0
CTNNB1	DACH1	0.4353	0
CTNNB1	DVL1	-0.6811	1
CTNNB1	E2F1	0.4119	0
CTNNB1	EPHA2	0.2991	0
CTNNB1	FGFR4	0.0056	0
CTNNB1	FZR1	0.0633	0
CTNNB1	GLIS2	-0.2351	0
CTNNB1	GRM1	-0.2760	0
CTNNB1	GSK3B	-0.3801	0
CTNNB1	HGF	-0.4804	0
CTNNB1	HIF1A	-0.4954	0
CTNNB1	IGF1R	0.6591	1
CTNNB1	JAK1	-0.2660	0
CTNNB1	JAK2	-0.5923	1
CTNNB1	KAT2A	-0.0030	0
CTNNB1	KDELR2	-0.2748	0
CTNNB1	KEAP1	0.0439	0
CTNNB1	LATS2	-0.2745	0
CTNNB1	MAP2K1	-0.8918	1
CTNNB1	MAP2K3	0.6381	1
CTNNB1	MAP2K4	-0.3676	0
CTNNB1	MAP2K5	0.2855	0
CTNNB1	MAP2K6	-0.4572	0
CTNNB1	MAP3K5	-0.4252	0
CTNNB1	MAPK1	0.6747	1
CTNNB1	MAPK14	-0.0616	0
CTNNB1	MAPK3	-0.0674	0
CTNNB1	MAPK8	-0.0743	0
CTNNB1	MAPK9	0.1393	0
CTNNB1	MDM4	-0.5804	1
CTNNB1	MSH2	-0.5802	1
CTNNB1	MTOR	-0.7002	1
CTNNB1	NF1	0.2619	0
CTNNB1	NF2	-0.3750	0

GENE1	GENE2	R	Positive
CTNNB1	NFIC	-0.1599	0
CTNNB1	NFKB2	0.5675	1
CTNNB1	NOTCH1	-0.2572	0
CTNNB1	PIK3R1	-0.6916	1
CTNNB1	PRKAA2	-0.6322	1
CTNNB1	PTEN	0.2413	0
CTNNB1	RASSF1	-0.1911	0
CTNNB1	RB1	0.4098	0
CTNNB1	RELA	0.7251	1
CTNNB1	ROS1	0.5608	1
CTNNB1	SFN	-0.3292	0
CTNNB1	SMAD1	-0.6149	1
CTNNB1	SMAD2	-0.0894	0
CTNNB1	SMAD4	0.6066	1
CTNNB1	SMARCA4	-0.4544	0
CTNNB1	SOX3	0.5651	1
CTNNB1	SOX4	-0.1556	0
CTNNB1	STAT1	-0.1954	0
CTNNB1	STAT2	0.3150	0
CTNNB1	STK11	0.2562	0
CTNNB1	STK3	-0.8854	1
CTNNB1	STK4	-0.1904	0
CTNNB1	TEAD2	0.2522	0
CTNNB1	TERT	-0.5844	1
CTNNB1	TNFRSF1A	-0.1200	0
CTNNB1	TP53	-0.4069	0
CTNNB1	TSC1	-0.1086	0
CTNNB1	WT1	0.9226	1
CTNNB1	YWHAZ	0.1724	0
DACH1	ABCB5	-0.8995	1
DACH1	AKT1S1	0.4391	0
DACH1	APC	-0.0828	0
DACH1	BAX	-0.7775	1
DACH1	BCL2	-0.6269	1
DACH1	CDC42	0.6324	1
DACH1	DVL1	-0.8382	1
DACH1	E2F1	0.2526	0
DACH1	GSK3B	-0.9770	1
DACH1	HGF	-0.2849	0
DACH1	HIF1A	-0.8871	1
DACH1	IGF1R	0.8323	1
DACH1	JAK1	-0.1897	0
DACH1	JAK2	0.1595	0
DACH1	KEAP1	0.1990	0
DACH1	MAP2K1	-0.1762	0
DACH1	MAP2K4	-0.7961	1
DACH1	MAP2K6	-0.9727	1
DACH1	MAP3K5	0.0790	0
DACH1	MAPK1	-0.0809	0
DACH1	MAPK14	-0.5935	1
DACH1	MAPK3	-0.6873	1
DACH1	MAPK8	0.3646	0
DACH1	MAPK9	0.1567	0
DACH1	MDM4	-0.1361	0
DACH1	MTOR	-0.3468	0
DACH1	NFIC	-0.7513	1
DACH1	NFKB2	0.4573	0
DACH1	NOTCH1	0.0238	0
DACH1	PRKAA2	-0.4263	0
DACH1	RELA	0.0870	0
DACH1	SFN	0.1046	0
DACH1	SMAD1	-0.4959	0
DACH1	SMAD4	0.2099	0
DACH1	SOX3	0.3824	0
DACH1	SOX4	-0.4378	0
DACH1	STAT1	-0.8518	1
DACH1	STAT2	-0.0114	0
DACH1	STK3	-0.5170	1
DACH1	STK4	-0.0358	0
DACH1	TERT	-0.0040	0
DACH1	TNFRSF1A	0.4346	0
DACH1	YWHAZ	0.0596	0
E2F1	APC	0.1282	0
E2F1	BAX	-0.7188	1
E2F1	BCL2	0.5394	1
E2F1	CDC42	-0.0828	0
E2F1	DVL1	-0.2754	0
E2F1	GSK3B	-0.1194	0
E2F1	JAK1	-0.6886	1
E2F1	JAK2	-0.5131	1
E2F1	MAP2K1	-0.4901	0
E2F1	MAP2K4	-0.6890	1
E2F1	MAPK1	0.0791	0
E2F1	MAPK3	0.4581	0
E2F1	MAPK8	-0.2466	0
E2F1	MAPK9	0.9415	1
E2F1	MTOR	-0.6297	1
E2F1	NFKB2	0.2900	0
E2F1	NOTCH1	0.3375	0
E2F1	PRKAA2	-0.2783	0
E2F1	SMAD1	0.0060	0
E2F1	SMAD4	0.7756	1
E2F1	STAT1	0.1602	0
E2F1	STAT2	0.1716	0
EGFR	ABCB5	0.3935	0
EGFR	AKT1S1	0.3264	0
EGFR	APC	0.4238	0
EGFR	ARNT	-0.3156	0

GENE1	GENE2	R	Positive
EGFR	BAX	0.3605	0
EGFR	BCL2	0.6643	1
EGFR	BECN1	-0.1742	0
EGFR	CBLB	-0.3747	0
EGFR	CCND2	-0.5967	1
EGFR	CCNE1	0.8622	1
EGFR	CD44	0.1812	0
EGFR	CDC42	0.0103	0
EGFR	CDK4	-0.1104	0
EGFR	CDK6	-0.3866	0
EGFR	CDKN2A	0.8039	1
EGFR	CDKN2B	-0.1073	0
EGFR	CDKN2C	-0.8836	1
EGFR	CTNNB1	0.1642	0
EGFR	DACH1	-0.5641	1
EGFR	DVL1	0.5604	1
EGFR	E2F1	0.0733	0
EGFR	EPHA2	0.9144	1
EGFR	FGFR4	0.3343	0
EGFR	FZR1	-0.2581	0
EGFR	GLIS2	-0.1169	0
EGFR	GRM1	-0.6315	1
EGFR	GSK3B	0.5564	1
EGFR	HGF	-0.3864	0
EGFR	HIF1A	0.3269	0
EGFR	IGF1R	-0.1273	0
EGFR	JAK1	-0.3912	0
EGFR	JAK2	-0.0972	0
EGFR	KAT2A	-0.9509	1
EGFR	KDELR2	0.4205	0
EGFR	KEAP1	-0.5737	1
EGFR	LATS2	0.1276	0
EGFR	MAP2K1	-0.0902	0
EGFR	MAP2K3	-0.5047	1
EGFR	MAP2K4	0.2008	0
EGFR	MAP2K5	-0.3188	0
EGFR	MAP2K6	0.3831	0
EGFR	MAP3K5	-0.8627	1
EGFR	MAPK1	0.6670	1
EGFR	MAPK14	0.0128	0
EGFR	MAPK3	0.3372	0
EGFR	MAPK8	0.1427	0
EGFR	MAPK9	-0.1157	0
EGFR	MDM4	-0.5264	1
EGFR	MSH2	0.1133	0
EGFR	MTOR	0.0755	0
EGFR	NF1	-0.3315	0
EGFR	NF2	-0.5430	1
EGFR	NFIC	0.4657	0

GENE1	GENE2	R	Positive
EGFR	NFKB2	0.2896	0
EGFR	NOTCH1	0.4381	0
EGFR	PIK3R1	0.0163	0
EGFR	PRKAA2	-0.4521	0
EGFR	PTEN	-0.7722	1
EGFR	RASSF1	-0.5324	1
EGFR	RB1	0.2336	0
EGFR	RELA	0.6040	1
EGFR	ROS1	-0.4903	0
EGFR	SFN	-0.0633	0
EGFR	SMAD1	-0.3491	0
EGFR	SMAD2	0.2335	0
EGFR	SMAD4	-0.1527	0
EGFR	SMARCA4	0.0313	0
EGFR	SOX3	0.4965	0
EGFR	SOX4	0.7627	1
EGFR	STAT1	0.7634	1
EGFR	STAT2	0.4918	0
EGFR	STK11	-0.0950	0
EGFR	STK3	0.0203	0
EGFR	STK4	0.3228	0
EGFR	TEAD2	0.4774	0
EGFR	TERT	-0.2975	0
EGFR	TNFRSF1A	-0.3568	0
EGFR	TP53	0.6373	1
EGFR	TSC1	0.3143	0
EGFR	WT1	0.4205	0
EGFR	YWHAZ	0.5777	1
EPHA2	ABCB5	0.1813	0
EPHA2	AKT1S1	0.6224	1
EPHA2	APC	0.1997	0
EPHA2	ARNT	0.0515	0
EPHA2	BAX	0.0349	0
EPHA2	BCL2	0.5663	1
EPHA2	BECN1	-0.2648	0
EPHA2	CCND2	-0.3489	0
EPHA2	CCNE1	0.8082	1
EPHA2	CD44	0.4930	0
EPHA2	CDC42	0.2719	0
EPHA2	CDK4	-0.0352	0
EPHA2	CDK6	-0.1444	0
EPHA2	CDKN2C	-0.8568	1
EPHA2	DACH1	-0.3632	0
EPHA2	DVL1	0.3062	0
EPHA2	E2F1	0.2422	0
EPHA2	FZR1	-0.0391	0
EPHA2	GLIS2	-0.1755	0
EPHA2	GRM1	-0.5945	1
EPHA2	GSK3B	0.3209	0

GENE1	GENE2	R	Positive
EPHA2	HGF	-0.3031	0
EPHA2	HIF1A	0.1729	0
EPHA2	IGF1R	0.1830	0
EPHA2	JAK1	-0.6757	1
EPHA2	JAK2	-0.0623	0
EPHA2	KAT2A	-0.8055	1
EPHA2	KDELR2	0.5285	1
EPHA2	KEAP1	-0.3538	0
EPHA2	MAP2K1	-0.1603	0
EPHA2	MAP2K3	-0.3891	0
EPHA2	MAP2K4	-0.0205	0
EPHA2	MAP2K5	-0.1162	0
EPHA2	MAP2K6	0.2035	0
EPHA2	MAP3K5	-0.8734	1
EPHA2	MAPK1	0.8132	1
EPHA2	MAPK14	-0.0478	0
EPHA2	MAPK3	0.2526	0
EPHA2	MAPK8	0.3276	0
EPHA2	MAPK9	0.0451	0
EPHA2	MDM4	-0.7477	1
EPHA2	MTOR	-0.2546	0
EPHA2	NFIC	0.2052	0
EPHA2	NFKB2	0.6053	1
EPHA2	NOTCH1	0.5369	1
EPHA2	PRKAA2	-0.4484	0
EPHA2	RELA	0.5248	1
EPHA2	SFN	-0.0437	0
EPHA2	SMAD1	-0.3714	0
EPHA2	SMAD4	0.0609	0
EPHA2	SOX3	0.5095	1
EPHA2	SOX4	0.6006	1
EPHA2	STAT1	0.7208	1
EPHA2	STAT2	0.2375	0
EPHA2	STK3	-0.0758	0
EPHA2	STK4	0.3450	0
EPHA2	TEAD2	0.5257	1
EPHA2	TERT	-0.5590	1
EPHA2	TNFRSF1A	-0.5038	1
EPHA2	YWHAZ	0.6721	1
ERBB2	ABCB5	0.1765	0
ERBB2	AKT1S1	-0.1374	0
ERBB2	APC	0.7655	1
ERBB2	ARNT	-0.8933	1
ERBB2	BAX	0.3955	0
ERBB2	BCL2	0.3476	0
ERBB2	BECN1	-0.1539	0
ERBB2	BRAF	-0.0260	0
ERBB2	CBLB	-0.1683	0
ERBB2	CBLC	-0.4994	0

GENE1	GENE2	R	Positive
ERBB2	CCND2	-0.7301	1
ERBB2	CCNE1	0.5159	1
ERBB2	CD44	-0.5215	1
ERBB2	CDC42	-0.1841	0
ERBB2	CDK4	-0.0379	0
ERBB2	CDK6	-0.2120	0
ERBB2	CDKN2A	0.5280	1
ERBB2	CDKN2B	-0.3718	0
ERBB2	CDKN2C	-0.2472	0
ERBB2	CTNNB1	0.1176	0
ERBB2	DACH1	-0.1553	0
ERBB2	DVL1	0.4119	0
ERBB2	E2F1	-0.0280	0
ERBB2	EGFR	0.5394	1
ERBB2	EPHA2	0.2349	0
ERBB2	FGFR4	0.3700	0
ERBB2	FOXO1	-0.4759	0
ERBB2	FZR1	-0.0940	0
ERBB2	GLIS2	-0.1314	0
ERBB2	GRM1	-0.5782	1
ERBB2	GSK3B	0.2658	0
ERBB2	HGF	-0.7543	1
ERBB2	HIF1A	0.0080	0
ERBB2	HRAS	-0.0290	0
ERBB2	IGF1R	-0.1927	0
ERBB2	JAK1	0.1488	0
ERBB2	JAK2	-0.0778	0
ERBB2	KAT2A	-0.4890	0
ERBB2	KDELR2	-0.1872	0
ERBB2	KEAP1	-0.7167	1
ERBB2	KRAS	0.1993	0
ERBB2	LATS2	-0.0436	0
ERBB2	MAP2K1	-0.0453	0
ERBB2	MAP2K3	0.0075	0
ERBB2	MAP2K4	0.0050	0
ERBB2	MAP2K5	-0.6684	1
ERBB2	MAP2K6	-0.0039	0
ERBB2	MAP3K5	-0.5566	1
ERBB2	MAPK1	0.0592	0
ERBB2	MAPK14	-0.2316	0
ERBB2	MAPK3	0.0268	0
ERBB2	MAPK8	-0.0968	0
ERBB2	MAPK9	-0.2113	0
ERBB2	MDM2	-0.1706	0
ERBB2	MDM4	-0.0556	0
ERBB2	MET	-0.2265	0
ERBB2	MSH2	-0.2959	0
ERBB2	MTOR	0.5334	1
ERBB2	NF1	-0.2022	0

GENE1	GENE2	R	Positive
ERBB2	NF2	-0.3805	0
ERBB2	NFIC	0.3156	0
ERBB2	NFKB2	-0.1883	0
ERBB2	NOTCH1	0.1858	0
ERBB2	PIK3CA	0.1348	0
ERBB2	PIK3R1	0.1363	0
ERBB2	PRKAA2	-0.6615	1
ERBB2	PTEN	-0.7135	1
ERBB2	RASSF1	0.0808	0
ERBB2	RB1	0.2869	0
ERBB2	RELA	0.6529	1
ERBB2	ROS1	-0.5369	1
ERBB2	SFN	-0.1239	0
ERBB2	SMAD1	-0.5291	1
ERBB2	SMAD2	0.2045	0
ERBB2	SMAD4	-0.3860	0
ERBB2	SMARCA4	-0.2563	0
ERBB2	SOX3	0.5933	1
ERBB2	SOX4	0.6986	1
ERBB2	STAT1	0.0880	0
ERBB2	STAT2	0.9687	1
ERBB2	STK11	-0.4997	0
ERBB2	STK3	-0.1833	0
ERBB2	STK4	0.0691	0
ERBB2	TCF3	-0.3228	0
ERBB2	TEAD2	0.5393	1
ERBB2	TERT	0.4305	0
ERBB2	TNFRSF1A	0.4598	0
ERBB2	TP53	0.2928	0
ERBB2	TSC1	-0.3832	0
ERBB2	WHSC1L1	0.1352	0
ERBB2	WT1	0.0968	0
ERBB2	YAP1	0.0758	0
ERBB2	YWHAZ	0.1949	0
FGFR4	ABCB5	0.4170	0
FGFR4	AKT1S1	-0.0960	0
FGFR4	APC	0.2168	0
FGFR4	ARNT	0.0094	0
FGFR4	BAX	-0.2318	0
FGFR4	BCL2	0.7363	1
FGFR4	BECN1	0.4642	0
FGFR4	CCND2	0.0642	0
FGFR4	CCNE1	0.4262	0
FGFR4	CD44	0.2464	0
FGFR4	CDC42	-0.3708	0
FGFR4	CDK4	-0.6428	1
FGFR4	CDK6	-0.1530	0
FGFR4	CDKN2C	-0.4031	0
FGFR4	DACH1	-0.1279	0
FGFR4	DVL1	0.2932	0
FGFR4	E2F1	0.8082	1
FGFR4	EPHA2	0.2992	0
FGFR4	FZR1	0.0953	0
FGFR4	GLIS2	0.6395	1
FGFR4	GRM1	-0.8987	1
FGFR4	GSK3B	0.2821	0
FGFR4	HGF	-0.6690	1
FGFR4	HIF1A	0.4171	0
FGFR4	IGF1R	0.0112	0
FGFR4	JAK1	-0.5251	1
FGFR4	JAK2	-0.3280	0
FGFR4	KAT2A	-0.1260	0
FGFR4	KDELR2	0.6751	1
FGFR4	KEAP1	0.2280	0
FGFR4	MAP2K1	-0.1274	0
FGFR4	MAP2K3	0.1295	0
FGFR4	MAP2K4	-0.4453	0
FGFR4	MAP2K5	-0.8479	1
FGFR4	MAP2K6	0.0988	0
FGFR4	MAP3K5	-0.2371	0
FGFR4	MAPK1	-0.1389	0
FGFR4	MAPK14	0.3018	0
FGFR4	MAPK3	0.5989	1
FGFR4	MAPK8	-0.2941	0
FGFR4	MAPK9	0.7910	1
FGFR4	MDM4	-0.5861	1
FGFR4	MTOR	-0.0880	0
FGFR4	NFIC	-0.3962	0
FGFR4	NFKB2	-0.0581	0
FGFR4	NOTCH1	0.5439	1
FGFR4	PRKAA2	-0.2586	0
FGFR4	RELA	-0.0237	0
FGFR4	SFN	-0.7541	1
FGFR4	SMAD1	0.0899	0
FGFR4	SMAD4	0.3477	0
FGFR4	SOX3	0.0475	0
FGFR4	SOX4	0.7603	1
FGFR4	STAT1	0.4123	0
FGFR4	STAT2	0.4592	0
FGFR4	STK3	-0.3420	0
FGFR4	STK4	-0.5598	1
FGFR4	TEAD2	0.5549	1
FGFR4	TERT	0.3745	0
FGFR4	TNFRSF1A	0.4960	0
FGFR4	YWHAZ	-0.2722	0
FOXO1	ABCB5	-0.8744	1
FOXO1	AKT1S1	0.2749	0
FOXO1	APC	-0.1449	0

GENE1	GENE2	R	Positive
FOXO1	ARNT	0.2842	0
FOXO1	BAX	-0.5806	1
FOXO1	BCL2	-0.7478	1
FOXO1	BECN1	-0.2832	0
FOXO1	CBLB	-0.1952	0
FOXO1	CCND2	0.2509	0
FOXO1	CCNE1	-0.4979	0
FOXO1	CD44	0.0759	0
FOXO1	CDC42	0.4842	0
FOXO1	CDK4	0.3903	0
FOXO1	CDK6	0.8259	1
FOXO1	CDKN2A	-0.6679	1
FOXO1	CDKN2B	0.2749	0
FOXO1	CDKN2C	0.8569	1
FOXO1	CTNNB1	0.4296	0
FOXO1	DACH1	0.8016	1
FOXO1	DVL1	-0.9209	1
FOXO1	E2F1	-0.0811	0
FOXO1	EGFR	-0.7342	1
FOXO1	EPHA2	-0.5361	1
FOXO1	FGFR4	-0.5802	1
FOXO1	FZR1	0.3773	0
FOXO1	GLIS2	-0.3835	0
FOXO1	GRM1	0.5876	1
FOXO1	GSK3B	-0.8369	1
FOXO1	HGF	0.2099	0
FOXO1	HIF1A	-0.7721	1
FOXO1	IGF1R	0.5940	1
FOXO1	JAK1	0.1782	0
FOXO1	JAK2	0.0039	0
FOXO1	KAT2A	0.7497	1
FOXO1	KDELR2	-0.6670	1
FOXO1	KEAP1	0.3214	0
FOXO1	LATS2	0.0750	0
FOXO1	MAP2K1	-0.2977	0
FOXO1	MAP2K3	0.6910	1
FOXO1	MAP2K4	-0.3317	0
FOXO1	MAP2K5	0.6772	1
FOXO1	MAP2K6	-0.6961	1
FOXO1	MAP3K5	0.3824	0
FOXO1	MAPK1	-0.0220	0
FOXO1	MAPK14	-0.3133	0
FOXO1	MAPK3	-0.6190	1
FOXO1	MAPK8	0.1505	0
FOXO1	MAPK9	-0.1075	0
FOXO1	MDM4	0.1818	0
FOXO1	MSH2	-0.5945	1
FOXO1	MTOR	-0.4300	0
FOXO1	NF1	0.5179	1
FOXO1	NF2	-0.0402	0
FOXO1	NFIC	-0.4033	0
FOXO1	NFKB2	0.2792	0
FOXO1	NOTCH1	-0.4874	0
FOXO1	PIK3CA	-0.8064	1
FOXO1	PIK3R1	-0.3895	0
FOXO1	PRKAA2	-0.0220	0
FOXO1	PTEN	0.7066	1
FOXO1	RASSF1	0.1940	0
FOXO1	RB1	-0.2947	0
FOXO1	RELA	-0.0221	0
FOXO1	ROS1	0.6859	1
FOXO1	SFN	0.2245	0
FOXO1	SMAD1	-0.2216	0
FOXO1	SMAD2	-0.5353	1
FOXO1	SMAD4	0.2742	0
FOXO1	SMARCA4	-0.3687	0
FOXO1	SOX3	0.0460	0
FOXO1	SOX4	-0.8758	1
FOXO1	STAT1	-0.8436	1
FOXO1	STAT2	-0.3631	0
FOXO1	STK11	0.1355	0
FOXO1	STK3	-0.3672	0
FOXO1	STK4	-0.0269	0
FOXO1	TEAD2	-0.2551	0
FOXO1	TERT	-0.2666	0
FOXO1	TNFRSF1A	0.0312	0
FOXO1	TP53	-0.7064	1
FOXO1	TSC1	-0.4818	0
FOXO1	WT1	0.1253	0
FOXO1	YAP1	-0.4459	0
FOXO1	YWHAZ	-0.0866	0
FZR1	ABCB5	-0.6048	1
FZR1	AKT1S1	0.5535	1
FZR1	APC	-0.3834	0
FZR1	BAX	-0.6321	1
FZR1	BCL2	-0.5167	1
FZR1	CCNE1	-0.3517	0
FZR1	CD44	-0.0119	0
FZR1	CDC42	0.7545	1
FZR1	CDKN2C	0.2099	0
FZR1	DACH1	0.7909	1
FZR1	DVL1	-0.4150	0
FZR1	E2F1	0.2572	0
FZR1	GRM1	-0.1107	0
FZR1	GSK3B	-0.8127	1
FZR1	HGF	-0.2983	0
FZR1	HIF1A	-0.6994	1
FZR1	IGF1R	0.7619	1

GENE1	GENE2	R	Positive
FZR1	JAK1	-0.5303	1
FZR1	JAK2	0.5744	1
FZR1	KAT2A	0.4551	0
FZR1	KEAP1	0.0111	0
FZR1	MAP2K1	0.2895	0
FZR1	MAP2K4	-0.8542	1
FZR1	MAP2K6	-0.8459	1
FZR1	MAP3K5	-0.1755	0
FZR1	MAPK1	-0.0829	0
FZR1	MAPK14	-0.7694	1
FZR1	MAPK3	-0.6727	1
FZR1	MAPK8	0.7300	1
FZR1	MAPK9	0.1923	0
FZR1	MDM4	-0.2877	0
FZR1	MTOR	-0.1215	0
FZR1	NFIC	-0.7881	1
FZR1	NFKB2	0.5868	1
FZR1	NOTCH1	0.6025	1
FZR1	PRKAA2	-0.4409	0
FZR1	RELA	-0.0547	0
FZR1	SFN	0.2918	0
FZR1	SMAD1	-0.4697	0
FZR1	SMAD4	-0.0970	0
FZR1	SOX3	0.4520	0
FZR1	SOX4	-0.0243	0
FZR1	STAT1	-0.5160	1
FZR1	STAT2	-0.0343	0
FZR1	STK3	-0.1389	0
FZR1	STK4	0.2694	0
FZR1	TEAD2	0.6476	1
FZR1	TERT	0.0941	0
FZR1	TNFRSF1A	0.3967	0
FZR1	YWHAZ	0.3701	0
GLIS2	ABCB5	0.6306	1
GLIS2	AKT1S1	-0.6026	1
GLIS2	APC	-0.0148	0
GLIS2	ARNT	0.2231	0
GLIS2	BAX	-0.0332	0
GLIS2	BCL2	0.6397	1
GLIS2	BECN1	0.9668	1
GLIS2	CCNE1	0.0603	0
GLIS2	CD44	0.2805	0
GLIS2	CDC42	-0.8237	1
GLIS2	CDKN2C	-0.0910	0
GLIS2	DACH1	-0.3900	0
GLIS2	DVL1	0.2501	0
GLIS2	E2F1	0.5455	1
GLIS2	FZR1	-0.4256	0
GLIS2	GRM1	-0.3174	0

GENE1	GENE2	R	Positive
GLIS2	GSK3B	0.5159	1
GLIS2	HGF	-0.0064	0
GLIS2	HIF1A	0.7587	1
GLIS2	IGF1R	-0.4325	0
GLIS2	JAK1	-0.0135	0
GLIS2	JAK2	-0.5660	1
GLIS2	KAT2A	0.1369	0
GLIS2	KDELR2	0.5993	1
GLIS2	KEAP1	0.6697	1
GLIS2	MAP2K1	-0.1740	0
GLIS2	MAP2K4	0.0762	0
GLIS2	MAP2K6	0.5255	1
GLIS2	MAP3K5	0.4629	0
GLIS2	MAPK1	-0.4428	0
GLIS2	MAPK14	0.8596	1
GLIS2	MAPK3	0.8650	1
GLIS2	MAPK8	-0.7788	1
GLIS2	MAPK9	0.7329	1
GLIS2	MDM4	-0.0799	0
GLIS2	MTOR	-0.1354	0
GLIS2	NFIC	-0.1466	0
GLIS2	NFKB2	-0.5543	1
GLIS2	NOTCH1	-0.0889	0
GLIS2	PRKAA2	0.4925	0
GLIS2	RELA	-0.5113	1
GLIS2	SFN	-0.8153	1
GLIS2	SMAD1	0.7587	1
GLIS2	SMAD4	0.5219	1
GLIS2	SOX3	-0.6932	1
GLIS2	SOX4	0.2206	0
GLIS2	STAT1	0.3976	0
GLIS2	STAT2	-0.0735	0
GLIS2	STK3	-0.1063	0
GLIS2	STK4	-0.8504	1
GLIS2	TEAD2	-0.2806	0
GLIS2	TERT	0.3459	0
GLIS2	TNFRSF1A	0.2382	0
GLIS2	YWHAZ	-0.8212	1
GRM1	ABCB5	-0.2535	0
GRM1	AKT1S1	-0.2013	0
GRM1	APC	-0.4606	0
GRM1	BAX	0.1941	0
GRM1	BCL2	-0.7578	1
GRM1	CDC42	0.1332	0
GRM1	CDKN2C	0.5427	1
GRM1	DACH1	0.1104	0
GRM1	DVL1	-0.2594	0
GRM1	E2F1	-0.7258	1
GRM1	GSK3B	-0.2429	0

GENE1	GENE2	R	Positive
GRM1	HGF	0.8382	1
GRM1	HIF1A	-0.2281	0
GRM1	IGF1R	-0.1671	0
GRM1	JAK1	0.6092	1
GRM1	JAK2	0.3280	0
GRM1	KAT2A	0.4009	0
GRM1	KEAP1	0.0937	0
GRM1	MAP2K1	0.2685	0
GRM1	MAP2K4	0.4390	0
GRM1	MAP2K6	-0.0007	0
GRM1	MAP3K5	0.6230	1
GRM1	MAPK1	-0.2412	0
GRM1	MAPK14	-0.0909	0
GRM1	MAPK3	-0.4549	0
GRM1	MAPK8	0.1077	0
GRM1	MAPK9	-0.5685	1
GRM1	MDM4	0.7545	1
GRM1	MTOR	0.1280	0
GRM1	NFIC	0.2313	0
GRM1	NFKB2	-0.2103	0
GRM1	NOTCH1	-0.5845	1
GRM1	PRKAA2	0.5939	1
GRM1	RELA	-0.3919	0
GRM1	SFN	0.6181	1
GRM1	SMAD1	0.2892	0
GRM1	SMAD4	-0.2669	0
GRM1	SOX3	-0.4329	0
GRM1	SOX4	-0.8534	1
GRM1	STAT1	-0.4553	0
GRM1	STAT2	-0.6709	1
GRM1	STK3	0.4587	0
GRM1	STK4	0.3040	0
GRM1	TERT	-0.1290	0
GRM1	TNFRSF1A	-0.3314	0
GRM1	YWHAZ	-0.0877	0
HGF	ABCB5	0.1599	0
HGF	AKT1S1	-0.2277	0
HGF	APC	-0.6718	1
HGF	BAX	0.2854	0
HGF	BCL2	-0.3963	0
HGF	CDC42	-0.0553	0
HGF	DVL1	0.0525	0
HGF	E2F1	-0.5693	1
HGF	GSK3B	0.1309	0
HGF	HIF1A	0.2253	0
HGF	IGF1R	-0.3655	0
HGF	JAK1	0.3525	0
HGF	JAK2	0.2720	0
HGF	KEAP1	0.2671	0

GENE1	GENE2	R	Positive
HGF	MAP2K1	0.3598	0
HGF	MAP2K4	0.5878	1
HGF	MAP2K6	0.4026	0
HGF	MAP3K5	0.5867	1
HGF	MAPK1	-0.1631	0
HGF	MAPK14	0.2385	0
HGF	MAPK3	-0.0460	0
HGF	MAPK8	0.0408	0
HGF	MAPK9	-0.3388	0
HGF	MDM4	0.5560	1
HGF	MTOR	0.0316	0
HGF	NFIC	0.2976	0
HGF	NFKB2	-0.2152	0
HGF	NOTCH1	-0.3709	0
HGF	PRKAA2	0.8532	1
HGF	RELA	-0.6147	1
HGF	SFN	0.4614	0
HGF	SMAD1	0.6268	1
HGF	SMAD4	-0.1496	0
HGF	SOX3	-0.6890	1
HGF	SOX4	-0.6288	1
HGF	STAT1	0.0492	0
HGF	STAT2	-0.8746	1
HGF	STK3	0.6614	1
HGF	STK4	0.2418	0
HGF	TERT	-0.2489	0
HGF	TNFRSF1A	-0.5942	1
HIF1A	ABCB5	0.9595	1
HIF1A	AKT1S1	-0.5906	1
HIF1A	APC	-0.0424	0
HIF1A	BAX	0.5218	1
HIF1A	BCL2	0.7237	1
HIF1A	CDC42	-0.8128	1
HIF1A	DVL1	0.7426	1
HIF1A	E2F1	0.0888	0
HIF1A	GSK3B	0.9226	1
HIF1A	IGF1R	-0.7886	1
HIF1A	JAK1	0.0791	0
HIF1A	JAK2	-0.3022	0
HIF1A	KEAP1	0.1973	0
HIF1A	MAP2K1	0.1293	0
HIF1A	MAP2K4	0.5656	1
HIF1A	MAP3K5	0.1794	0
HIF1A	MAPK1	-0.1968	0
HIF1A	MAPK3	0.8807	1
HIF1A	MAPK8	-0.5692	1
HIF1A	MAPK9	0.2655	0
HIF1A	MDM4	0.0558	0
HIF1A	MTOR	0.2019	0

GENE1	GENE2	R	Positive
HIF1A	NFIC	0.4103	0
HIF1A	NFKB2	-0.5739	1
HIF1A	NOTCH1	0.0214	0
HIF1A	PRKAA2	0.5678	1
HIF1A	RELA	-0.3796	0
HIF1A	SMAD1	0.7453	1
HIF1A	SMAD4	0.0673	0
HIF1A	STAT1	0.8026	1
HIF1A	STAT2	-0.0758	0
HIF1A	STK3	0.3780	0
HIF1A	STK4	-0.3512	0
HIF1A	TNFRSF1A	-0.1805	0
HRAS	ABCB5	0.6138	1
HRAS	AKT1S1	-0.0918	0
HRAS	APC	0.2507	0
HRAS	ARNT	0.0785	0
HRAS	BAX	0.3106	0
HRAS	BCL2	0.8068	1
HRAS	BECN1	0.5278	1
HRAS	CBLB	0.3255	0
HRAS	CCND2	-0.3642	0
HRAS	CCNE1	0.6714	1
HRAS	CD44	0.5271	1
HRAS	CDC42	-0.5081	1
HRAS	CDK4	-0.6607	1
HRAS	CDK6	-0.4362	0
HRAS	CDKN2A	0.0873	0
HRAS	CDKN2B	0.5932	1
HRAS	CDKN2C	-0.6245	1
HRAS	CTNNB1	0.1115	0
HRAS	DACH1	-0.7942	1
HRAS	DVL1	0.4052	0
HRAS	E2F1	0.1684	0
HRAS	EGFR	0.5944	1
HRAS	EPHA2	0.5567	1
HRAS	FGFR4	0.2303	0
HRAS	FOXO1	-0.5621	1
HRAS	FZR1	-0.7790	1
HRAS	GLIS2	0.4728	0
HRAS	GRM1	-0.2819	0
HRAS	GSK3B	0.8000	1
HRAS	HGF	0.1321	0
HRAS	HIF1A	0.7508	1
HRAS	IGF1R	-0.4323	0
HRAS	JAK1	-0.1161	0
HRAS	JAK2	-0.5990	1
HRAS	KAT2A	-0.6515	1
HRAS	KDELR2	0.5656	1
HRAS	KEAP1	0.1187	0

GENE1	GENE2	R	Positive
HRAS	KRAS	0.6961	1
HRAS	LATS2	-0.1333	0
HRAS	MAP2K1	-0.3841	0
HRAS	MAP2K3	-0.4992	0
HRAS	MAP2K4	0.5453	1
HRAS	MAP2K5	0.0243	0
HRAS	MAP2K6	0.8029	1
HRAS	MAP3K5	-0.1980	0
HRAS	MAPK1	0.4530	0
HRAS	MAPK14	0.7666	1
HRAS	MAPK3	0.8355	1
HRAS	MAPK8	-0.4881	0
HRAS	MAPK9	0.1650	0
HRAS	MDM4	-0.3162	0
HRAS	MSH2	0.6736	1
HRAS	MTOR	-0.2670	0
HRAS	NF1	0.1146	0
HRAS	NF2	0.0555	0
HRAS	NFIC	0.5252	1
HRAS	NFKB2	-0.1013	0
HRAS	NOTCH1	-0.1461	0
HRAS	PIK3CA	0.2638	0
HRAS	PIK3R1	0.2093	0
HRAS	PRKAA2	0.2526	0
HRAS	PTEN	-0.0567	0
HRAS	RASSF1	-0.7111	1
HRAS	RB1	0.3693	0
HRAS	RELA	0.1209	0
HRAS	ROS1	0.1647	0
HRAS	SFN	-0.4487	0
HRAS	SMAD1	0.3696	0
HRAS	SMAD2	0.7511	1
HRAS	SMAD4	0.3679	0
HRAS	SMARCA4	0.6687	1
HRAS	SOX3	-0.2708	0
HRAS	SOX4	0.2948	0
HRAS	STAT1	0.8634	1
HRAS	STAT2	-0.0312	0
HRAS	STK11	0.6982	1
HRAS	STK3	-0.0037	0
HRAS	STK4	-0.2374	0
HRAS	TCF3	0.2522	0
HRAS	TEAD2	-0.2783	0
HRAS	TERT	-0.4271	0
HRAS	TNFRSF1A	-0.5999	1
HRAS	TP53	0.5967	1
HRAS	TSC1	0.8719	1
HRAS	WHSC1L1	0.6496	1
HRAS	WT1	0.4645	0

GENE1	GENE2	R	Positive
HRAS	YAP1	0.6182	1
HRAS	YWHAZ	-0.0981	0
IGF1R	APC	-0.0492	0
IGF1R	BAX	-0.8730	1
IGF1R	BCL2	-0.3168	0
IGF1R	CDC42	0.8009	1
IGF1R	DVL1	-0.7789	1
IGF1R	E2F1	0.4581	0
IGF1R	GSK3B	-0.8413	1
IGF1R	JAK1	-0.6215	1
IGF1R	JAK2	0.0532	0
IGF1R	KEAP1	0.1616	0
IGF1R	MAP2K1	-0.3478	0
IGF1R	MAP2K4	-0.8310	1
IGF1R	MAP3K5	-0.3330	0
IGF1R	MAPK1	0.4083	0
IGF1R	MAPK3	-0.5043	1
IGF1R	MAPK8	0.5167	1
IGF1R	MAPK9	0.2685	0
IGF1R	MDM4	-0.6053	1
IGF1R	MTOR	-0.6523	1
IGF1R	NFIC	-0.7060	1
IGF1R	NFKB2	0.8520	1
IGF1R	NOTCH1	0.2668	0
IGF1R	PRKAA2	-0.5923	1
IGF1R	RELA	0.3083	0
IGF1R	SMAD1	-0.6255	1
IGF1R	SMAD4	0.3926	0
IGF1R	STAT1	-0.4489	0
IGF1R	STAT2	-0.0221	0
IGF1R	STK3	-0.6093	1
IGF1R	STK4	0.0863	0
IGF1R	TNFRSF1A	0.0662	0
KAT2A	ABCB5	-0.5081	1
KAT2A	AKT1S1	-0.1496	0
KAT2A	APC	-0.3744	0
KAT2A	BAX	-0.6044	1
KAT2A	BCL2	-0.5857	1
KAT2A	CDC42	0.1028	0
KAT2A	CDKN2C	0.8595	1
KAT2A	DACH1	0.7326	1
KAT2A	DVL1	-0.6781	1
KAT2A	E2F1	0.1942	0
KAT2A	GSK3B	-0.6941	1
KAT2A	HGF	0.1501	0
KAT2A	HIF1A	-0.4317	0
KAT2A	IGF1R	0.3628	0
KAT2A	JAK1	0.1432	0
KAT2A	JAK2	0.0428	0

GENE1	GENE2	R	Positive
KAT2A	KEAP1	0.6401	1
KAT2A	MAP2K1	-0.0193	0
KAT2A	MAP2K4	-0.4854	0
KAT2A	MAP2K6	-0.5683	1
KAT2A	MAP3K5	0.7231	1
KAT2A	MAPK1	-0.5947	1
KAT2A	MAPK14	-0.0857	0
KAT2A	MAPK3	-0.3380	0
KAT2A	MAPK8	-0.0801	0
KAT2A	MAPK9	0.3303	0
KAT2A	MDM4	0.2651	0
KAT2A	MTOR	-0.2361	0
KAT2A	NFIC	-0.7036	1
KAT2A	NFKB2	-0.1024	0
KAT2A	NOTCH1	-0.2633	0
KAT2A	PRKAA2	0.2447	0
KAT2A	RELA	-0.5194	1
KAT2A	SFN	-0.0816	0
KAT2A	SMAD1	0.2056	0
KAT2A	SMAD4	0.3059	0
KAT2A	SOX3	-0.3354	0
KAT2A	SOX4	-0.6486	1
KAT2A	STAT1	-0.7860	1
KAT2A	STAT2	-0.3839	0
KAT2A	STK3	-0.2371	0
KAT2A	STK4	-0.4108	0
KAT2A	TERT	0.2778	0
KAT2A	TNFRSF1A	0.4800	0
KAT2A	YWHAZ	-0.5364	1
KDELR2	ABCB5	0.6322	1
KDELR2	AKT1S1	0.0429	0
KDELR2	APC	-0.3219	0
KDELR2	BAX	-0.0715	0
KDELR2	BCL2	0.6855	1
KDELR2	BECN1	0.4363	0
KDELR2	CCNE1	0.3133	0
KDELR2	CD44	0.5603	1
KDELR2	CDC42	-0.2596	0
KDELR2	CDKN2C	-0.7294	1
KDELR2	DACH1	-0.4842	0
KDELR2	DVL1	0.4645	0
KDELR2	E2F1	0.5518	1
KDELR2	FZR1	-0.0739	0
KDELR2	GRM1	-0.5433	1
KDELR2	GSK3B	0.4962	0
KDELR2	HGF	-0.0144	0
KDELR2	HIF1A	0.6840	1
KDELR2	IGF1R	-0.1420	0
KDELR2	JAK1	-0.6583	1

GENE1	GENE2	R	Positive
KDELR2	JAK2	-0.1022	0
KDELR2	KAT2A	-0.3253	0
KDELR2	KEAP1	0.3580	0
KDELR2	MAP2K1	0.1270	0
KDELR2	MAP2K4	-0.0770	0
KDELR2	MAP2K6	0.4814	0
KDELR2	MAP3K5	-0.1557	0
KDELR2	MAPK1	0.0720	0
KDELR2	MAPK14	0.4649	0
KDELR2	MAPK3	0.6898	1
KDELR2	MAPK8	-0.0683	0
KDELR2	MAPK9	0.6441	1
KDELR2	MDM4	-0.5499	1
KDELR2	MTOR	-0.2259	0
KDELR2	NFIC	-0.1778	0
KDELR2	NFKB2	0.0941	0
KDELR2	NOTCH1	0.5905	1
KDELR2	PRKAA2	0.2341	0
KDELR2	RELA	-0.3541	0
KDELR2	SFN	-0.4332	0
KDELR2	SMAD1	0.4564	0
KDELR2	SMAD4	0.2970	0
KDELR2	SOX3	-0.2952	0
KDELR2	SOX4	0.5561	1
KDELR2	STAT1	0.7956	1
KDELR2	STAT2	-0.1841	0
KDELR2	STK3	0.1549	0
KDELR2	STK4	-0.2343	0
KDELR2	TEAD2	0.2018	0
KDELR2	TERT	-0.0687	0
KDELR2	TNFRSF1A	-0.1818	0
KDELR2	YWHAZ	-0.0635	0
KEAP1	APC	-0.3981	0
KEAP1	BAX	-0.6007	1
KEAP1	BCL2	0.0874	0
KEAP1	CDC42	-0.2557	0
KEAP1	DVL1	-0.4336	0
KEAP1	E2F1	0.5757	1
KEAP1	GSK3B	-0.1515	0
KEAP1	JAK1	-0.1979	0
KEAP1	JAK2	-0.4147	0
KEAP1	MAP2K1	-0.2930	0
KEAP1	MAP2K4	-0.2702	0
KEAP1	MAPK1	-0.3046	0
KEAP1	MAPK3	0.3964	0
KEAP1	MAPK8	-0.4223	0
KEAP1	MAPK9	0.7532	1
KEAP1	MTOR	-0.6301	1
KEAP1	NFIC	-0.5753	1
KEAP1	NFKB2	-0.0761	0
KEAP1	NOTCH1	-0.2049	0
KEAP1	PRKAA2	0.5229	1
KEAP1	SMAD1	0.6270	1
KEAP1	SMAD4	0.7787	1
KEAP1	STAT1	-0.0701	0
KEAP1	STAT2	-0.5771	1
KEAP1	TNFRSF1A	0.0476	0
KRAS	ABCB5	0.8999	1
KRAS	AKT1S1	-0.4525	0
KRAS	APC	0.1017	0
KRAS	ARNT	-0.0164	0
KRAS	BAX	0.3262	0
KRAS	BCL2	0.8778	1
KRAS	BECN1	0.7248	1
KRAS	CBLB	0.5715	1
KRAS	CCND2	-0.0514	0
KRAS	CCNE1	0.4185	0
KRAS	CD44	0.1965	0
KRAS	CDC42	-0.7511	1
KRAS	CDK4	-0.7889	1
KRAS	CDK6	-0.6870	1
KRAS	CDKN2A	0.2592	0
KRAS	CDKN2B	0.1241	0
KRAS	CDKN2C	-0.6361	1
KRAS	CTNNB1	-0.3406	0
KRAS	DACH1	-0.7507	1
KRAS	DVL1	0.7027	1
KRAS	E2F1	0.3852	0
KRAS	EGFR	0.4510	0
KRAS	EPHA2	0.3135	0
KRAS	FGFR4	0.7216	1
KRAS	FOXO1	-0.8509	1
KRAS	FZR1	-0.5106	1
KRAS	GLIS2	0.8017	1
KRAS	GRM1	-0.5683	1
KRAS	GSK3B	0.8382	1
KRAS	HGF	-0.1290	0
KRAS	HIF1A	0.9264	1
KRAS	IGF1R	-0.5911	1
KRAS	JAK1	-0.1671	0
KRAS	JAK2	-0.3720	0
KRAS	KAT2A	-0.4445	0
KRAS	KDELR2	0.7921	1
KRAS	KEAP1	0.1875	0
KRAS	LATS2	-0.4676	0
KRAS	MAP2K1	0.0255	0
KRAS	MAP2K3	-0.4586	0
KRAS	MAP2K4	0.2658	0

GENE1	GENE2	R	Positive
KRAS	MAP2K5	-0.6232	1
KRAS	MAP2K6	0.7584	1
KRAS	MAP3K5	-0.0275	0
KRAS	MAPK1	-0.1436	0
KRAS	MAPK14	0.7153	1
KRAS	MAPK3	0.9142	1
KRAS	MAPK8	-0.5357	1
KRAS	MAPK9	0.4918	0
KRAS	MDM4	-0.2260	0
KRAS	MSH2	0.8083	1
KRAS	MTOR	0.1191	0
KRAS	NF1	-0.4144	0
KRAS	NF2	0.4846	0
KRAS	NFIC	0.1931	0
KRAS	NFKB2	-0.4293	0
KRAS	NOTCH1	0.2483	0
KRAS	PIK3CA	0.8087	1
KRAS	PIK3R1	0.2713	0
KRAS	PRKAA2	0.2795	0
KRAS	PTEN	-0.2432	0
KRAS	RASSF1	-0.0751	0
KRAS	RB1	0.5857	1
KRAS	RELA	-0.2322	0
KRAS	ROS1	-0.2897	0
KRAS	SFN	-0.6225	1
KRAS	SMAD1	0.5548	1
KRAS	SMAD2	0.7920	1
KRAS	SMAD4	0.1782	0
KRAS	SMARCA4	0.5328	1
KRAS	SOX3	-0.4029	0
KRAS	SOX4	0.6737	1
KRAS	STAT1	0.8109	1
KRAS	STAT2	0.1699	0
KRAS	STK11	0.2278	0
KRAS	STK3	0.1378	0
KRAS	STK4	-0.4590	0
KRAS	TCF3	-0.1778	0
KRAS	TEAD2	-0.0014	0
KRAS	TERT	0.2670	0
KRAS	TNFRSF1A	0.0324	0
KRAS	TP53	0.4500	0
KRAS	TSC1	0.7475	1
KRAS	WHSC1L1	0.9213	1
KRAS	WT1	-0.0146	0
KRAS	YAP1	0.7321	1
KRAS	YWHAZ	-0.3748	0
LATS2	ABCB5	-0.0870	0
LATS2	AKT1S1	0.1411	0
LATS2	APC	-0.2177	0

GENE1	GENE2	R	Positive
LATS2	ARNT	-0.2169	0
LATS2	BAX	0.5670	1
LATS2	BCL2	-0.5342	1
LATS2	BECN1	-0.6530	1
LATS2	CCND2	-0.1931	0
LATS2	CCNE1	-0.2142	0
LATS2	CD44	-0.4200	0
LATS2	CDC42	0.3965	0
LATS2	CDK4	0.7639	1
LATS2	CDK6	-0.2597	0
LATS2	CDKN2A	0.1970	0
LATS2	CDKN2B	-0.3519	0
LATS2	CDKN2C	-0.1075	0
LATS2	DACH1	-0.1877	0
LATS2	DVL1	0.2149	0
LATS2	E2F1	-0.9169	1
LATS2	EPHA2	0.0743	0
LATS2	FGFR4	-0.8086	1
LATS2	FZR1	-0.0681	0
LATS2	GLIS2	-0.7444	1
LATS2	GRM1	0.6206	1
LATS2	GSK3B	0.0102	0
LATS2	HGF	0.5270	1
LATS2	HIF1A	-0.2168	0
LATS2	IGF1R	-0.1998	0
LATS2	JAK1	0.3567	0
LATS2	JAK2	0.6262	1
LATS2	KAT2A	-0.3352	0
LATS2	KDELR2	-0.4193	0
LATS2	KEAP1	-0.6443	1
LATS2	MAP2K1	0.4804	0
LATS2	MAP2K3	-0.5527	1
LATS2	MAP2K4	0.5396	1
LATS2	MAP2K5	0.5077	1
LATS2	MAP2K6	0.1003	0
LATS2	MAP3K5	-0.1476	0
LATS2	MAPK1	0.2287	0
LATS2	MAPK14	-0.4712	0
LATS2	MAPK3	-0.5578	1
LATS2	MAPK8	0.5481	1
LATS2	MAPK9	-0.9300	1
LATS2	MDM4	0.4937	0
LATS2	MSH2	-0.1543	0
LATS2	MTOR	0.4414	0
LATS2	NF1	0.3792	0
LATS2	NFIC	0.6001	1
LATS2	NFKB2	0.0921	0
LATS2	NOTCH1	-0.0954	0
LATS2	PIK3R1	0.5127	1

GENE1	GENE2	R	Positive
LATS2	PRKAA2	0.1030	0
LATS2	PTEN	-0.3524	0
LATS2	RASSF1	-0.5336	1
LATS2	RB1	-0.9236	1
LATS2	RELA	0.1352	0
LATS2	SFN	0.9266	1
LATS2	SMAD1	-0.2039	0
LATS2	SMAD2	-0.3967	0
LATS2	SMAD4	-0.7443	1
LATS2	SMARCA4	0.1638	0
LATS2	SOX3	0.1610	0
LATS2	SOX4	-0.2539	0
LATS2	STAT1	-0.0497	0
LATS2	STAT2	-0.2294	0
LATS2	STK3	0.6566	1
LATS2	STK4	0.8925	1
LATS2	TEAD2	-0.2280	0
LATS2	TERT	-0.2527	0
LATS2	TNFRSF1A	-0.5236	1
LATS2	TP53	0.5440	1
LATS2	TSC1	-0.3066	0
LATS2	WT1	-0.3226	0
LATS2	YWHAZ	0.6227	1
MAP2K3	ABCB5	-0.7362	1
MAP2K3	AKT1S1	0.2054	0
MAP2K3	APC	0.2908	0
MAP2K3	ARNT	0.0536	0
MAP2K3	BAX	-0.7764	1
MAP2K3	BCL2	-0.2460	0
MAP2K3	BECN1	-0.0099	0
MAP2K3	CCNE1	-0.1048	0
MAP2K3	CD44	0.1329	0
MAP2K3	CDC42	0.2260	0
MAP2K3	CDKN2C	0.7277	1
MAP2K3	DACH1	0.8505	1
MAP2K3	DVL1	-0.8361	1
MAP2K3	E2F1	0.5047	1
MAP2K3	FZR1	0.4498	0
MAP2K3	GLIS2	-0.0166	0
MAP2K3	GRM1	-0.1158	0
MAP2K3	GSK3B	-0.7349	1
MAP2K3	HGF	-0.4859	0
MAP2K3	HIF1A	-0.6437	1
MAP2K3	IGF1R	0.6787	1
MAP2K3	JAK1	-0.0798	0
MAP2K3	JAK2	-0.3522	0
MAP2K3	KAT2A	0.6913	1
MAP2K3	KDELR2	-0.4223	0
MAP2K3	KEAP1	0.3662	0

GENE1	GENE2	R	Positive
MAP2K3	MAP2K1	-0.5655	1
MAP2K3	MAP2K4	-0.7175	1
MAP2K3	MAP2K6	-0.7816	1
MAP2K3	MAP3K5	0.1416	0
MAP2K3	MAPK1	-0.0932	0
MAP2K3	MAPK14	-0.1844	0
MAP2K3	MAPK3	-0.2807	0
MAP2K3	MAPK8	-0.1381	0
MAP2K3	MAPK9	0.3930	0
MAP2K3	MDM4	-0.2246	0
MAP2K3	MTOR	-0.4692	0
MAP2K3	NFIC	-0.6704	1
MAP2K3	NFKB2	0.2199	0
MAP2K3	NOTCH1	-0.2258	0
MAP2K3	PRKAA2	-0.4304	0
MAP2K3	RELA	0.1975	0
MAP2K3	SFN	-0.3743	0
MAP2K3	SMAD1	-0.3741	0
MAP2K3	SMAD4	0.5351	1
MAP2K3	SOX3	0.2700	0
MAP2K3	SOX4	-0.3354	0
MAP2K3	STAT1	-0.7262	1
MAP2K3	STAT2	0.2172	0
MAP2K3	STK3	-0.8318	1
MAP2K3	STK4	-0.4857	0
MAP2K3	TEAD2	0.2219	0
MAP2K3	TERT	0.0607	0
MAP2K3	TNFRSF1A	0.5464	1
MAP2K3	YWHAZ	-0.2966	0
MAP2K5	ABCB5	-0.5013	1
MAP2K5	AKT1S1	0.3348	0
MAP2K5	APC	-0.2686	0
MAP2K5	ARNT	0.3944	0
MAP2K5	BAX	-0.1451	0
MAP2K5	BCL2	-0.5265	1
MAP2K5	BECN1	-0.2718	0
MAP2K5	CCND2	0.0765	0
MAP2K5	CCNE1	-0.2465	0
MAP2K5	CD44	0.2936	0
MAP2K5	CDC42	0.4086	0
MAP2K5	CDK4	0.3737	0
MAP2K5	CDK6	0.3918	0
MAP2K5	CDKN2C	0.3532	0
MAP2K5	DACH1	0.1885	0
MAP2K5	DVL1	-0.5486	1
MAP2K5	E2F1	-0.4138	0
MAP2K5	FZR1	-0.1014	0
MAP2K5	GLIS2	-0.4321	0
MAP2K5	GRM1	0.7621	1

GENE1	GENE2	R	Positive
MAP2K5	GSK3B	-0.3334	0
MAP2K5	HGF	0.6713	1
MAP2K5	HIF1A	-0.3658	0
MAP2K5	IGF1R	0.2422	0
MAP2K5	JAK1	0.1872	0
MAP2K5	JAK2	0.0124	0
MAP2K5	KAT2A	0.1942	0
MAP2K5	KDELR2	-0.4028	0
MAP2K5	KEAP1	0.1645	0
MAP2K5	MAP2K1	-0.2210	0
MAP2K5	MAP2K3	0.0270	0
MAP2K5	MAP2K4	0.2689	0
MAP2K5	MAP2K6	-0.1099	0
MAP2K5	MAP3K5	0.2224	0
MAP2K5	MAPK1	0.3775	0
MAP2K5	MAPK14	-0.0472	0
MAP2K5	MAPK3	-0.3577	0
MAP2K5	MAPK8	0.1903	0
MAP2K5	MAPK9	-0.4151	0
MAP2K5	MDM4	0.1962	0
MAP2K5	MTOR	-0.4366	0
MAP2K5	NFIC	0.1686	0
MAP2K5	NFKB2	0.3200	0
MAP2K5	NOTCH1	-0.5514	1
MAP2K5	PRKAA2	0.2919	0
MAP2K5	RELA	-0.0062	0
MAP2K5	SFN	0.4439	0
MAP2K5	SMAD1	0.0028	0
MAP2K5	SMAD4	0.1562	0
MAP2K5	SOX3	-0.1289	0
MAP2K5	SOX4	-0.8492	1
MAP2K5	STAT1	-0.2653	0
MAP2K5	STAT2	-0.6573	1
MAP2K5	STK3	0.0469	0
MAP2K5	STK4	0.3034	0
MAP2K5	TEAD2	-0.5720	1
MAP2K5	TERT	-0.7230	1
MAP2K5	TNFRSF1A	-0.6959	1
MAP2K5	YWHAZ	0.1843	0
MAP2K6	ABCB5	0.8980	1
MAP2K6	AKT1S1	-0.5467	1
MAP2K6	APC	0.0065	0
MAP2K6	BAX	0.7109	1
MAP2K6	BCL2	0.5944	1
MAP2K6	CDC42	-0.7305	1
MAP2K6	DVL1	0.7457	1
MAP2K6	E2F1	-0.2156	0
MAP2K6	GSK3B	0.9572	1
MAP2K6	HIF1A	0.9314	1
MAP2K6	IGF1R	-0.8627	1
MAP2K6	JAK1	0.2627	0
MAP2K6	JAK2	-0.2487	0
MAP2K6	KEAP1	0.0028	0
MAP2K6	MAP2K1	0.1259	0
MAP2K6	MAP2K4	0.8150	1
MAP2K6	MAP3K5	0.1319	0
MAP2K6	MAPK1	-0.0343	0
MAP2K6	MAPK14	0.7336	1
MAP2K6	MAPK3	0.7520	1
MAP2K6	MAPK8	-0.4991	0
MAP2K6	MAPK9	-0.0662	0
MAP2K6	MDM4	0.2116	0
MAP2K6	MTOR	0.2652	0
MAP2K6	NFIC	0.6898	1
MAP2K6	NFKB2	-0.5544	1
MAP2K6	NOTCH1	-0.1624	0
MAP2K6	PRKAA2	0.5949	1
MAP2K6	RELA	-0.2435	0
MAP2K6	SFN	-0.1889	0
MAP2K6	SMAD1	0.6646	1
MAP2K6	SMAD4	-0.0825	0
MAP2K6	SOX3	-0.5735	1
MAP2K6	SOX4	0.2834	0
MAP2K6	STAT1	0.7903	1
MAP2K6	STAT2	-0.1297	0
MAP2K6	STK3	0.4949	0
MAP2K6	STK4	-0.1187	0
MAP2K6	TERT	0.0197	0
MAP2K6	TNFRSF1A	-0.4245	0
MAP3K5	APC	-0.4340	0
MAP3K5	BAX	-0.0215	0
MAP3K5	BCL2	-0.3477	0
MAP3K5	CDC42	-0.4426	0
MAP3K5	DVL1	-0.1861	0
MAP3K5	E2F1	-0.1389	0
MAP3K5	GSK3B	-0.0658	0
MAP3K5	JAK1	0.5409	1
MAP3K5	JAK2	-0.0591	0
MAP3K5	KEAP1	0.6432	1
MAP3K5	MAP2K1	0.1531	0
MAP3K5	MAP2K4	0.1928	0
MAP3K5	MAPK1	-0.7625	1
MAP3K5	MAPK3	0.0855	0
MAP3K5	MAPK8	-0.4574	0
MAP3K5	MAPK9	0.1471	0
MAP3K5	MDM4	0.6526	1
MAP3K5	MTOR	0.0597	0
MAP3K5	NFIC	-0.1601	0

GENE1	GENE2	R	Positive
MAP3K5	NFKB2	-0.6304	1
MAP3K5	NOTCH1	-0.5346	1
MAP3K5	PRKAA2	0.7962	1
MAP3K5	RELA	-0.7903	1
MAP3K5	SMAD1	0.7481	1
MAP3K5	SMAD4	0.1474	0
MAP3K5	STAT1	-0.3793	0
MAP3K5	STAT2	-0.5659	1
MAP3K5	STK3	0.2167	0
MAP3K5	STK4	-0.4645	0
MAP3K5	TNFRSF1A	0.2034	0
MAPK14	ABCB5	0.6108	1
MAPK14	AKT1S1	-0.5333	1
MAPK14	APC	0.1098	0
MAPK14	BAX	0.1459	0
MAPK14	BCL2	0.6575	1
MAPK14	CDC42	-0.8261	1
MAPK14	DVL1	0.2135	0
MAPK14	E2F1	0.2965	0
MAPK14	GSK3B	0.6668	1
MAPK14	HIF1A	0.7996	1
MAPK14	IGF1R	-0.5357	1
MAPK14	JAK1	0.1582	0
MAPK14	JAK2	-0.7317	1
MAPK14	KEAP1	0.5757	1
MAPK14	MAP2K1	-0.3815	0
MAPK14	MAP2K4	0.4423	0
MAPK14	MAP3K5	0.4092	0
MAPK14	MAPK1	-0.1206	0
MAPK14	MAPK3	0.9123	1
MAPK14	MAPK8	-0.8548	1
MAPK14	MAPK9	0.4454	0
MAPK14	MDM4	-0.0112	0
MAPK14	MTOR	-0.2528	0
MAPK14	NFIC	0.2343	0
MAPK14	NFKB2	-0.5121	1
MAPK14	NOTCH1	-0.4152	0
MAPK14	PRKAA2	0.5875	1
MAPK14	RELA	-0.3234	0
MAPK14	SFN	-0.7005	1
MAPK14	SMAD1	0.7527	1
MAPK14	SMAD4	0.5608	1
MAPK14	STAT1	0.5236	1
MAPK14	STAT2	-0.1916	0
MAPK14	STK3	-0.0780	0
MAPK14	STK4	-0.7137	1
MAPK14	TERT	-0.0312	0
MAPK14	TNFRSF1A	-0.2001	0
MDM2	ABCB5	0.0215	0

GENE1	GENE2	R	Positive
MDM2	AKT1S1	0.0308	0
MDM2	APC	-0.4598	0
MDM2	ARNT	-0.0754	0
MDM2	BAX	0.5558	1
MDM2	BCL2	-0.5868	1
MDM2	BECN1	-0.5730	1
MDM2	CBLB	-0.2839	0
MDM2	CCND2	0.0654	0
MDM2	CCNE1	-0.4182	0
MDM2	CD44	-0.4566	0
MDM2	CDC42	0.3482	0
MDM2	CDK4	0.7576	1
MDM2	CDK6	-0.3694	0
MDM2	CDKN2A	0.1853	0
MDM2	CDKN2B	-0.4778	0
MDM2	CDKN2C	-0.1075	0
MDM2	CTNNB1	-0.5149	1
MDM2	DACH1	-0.1950	0
MDM2	DVL1	0.2961	0
MDM2	E2F1	-0.8940	1
MDM2	EGFR	-0.0038	0
MDM2	EPHA2	-0.0385	0
MDM2	FGFR4	-0.7411	1
MDM2	FOXO1	0.0296	0
MDM2	FZR1	0.0244	0
MDM2	GLIS2	-0.6227	1
MDM2	GRM1	0.6647	1
MDM2	GSK3B	0.0112	0
MDM2	HGF	0.6358	1
MDM2	HIF1A	-0.1246	0
MDM2	HRAS	-0.2168	0
MDM2	IGF1R	-0.2643	0
MDM2	JAK1	0.3228	0
MDM2	JAK2	0.7725	1
MDM2	KAT2A	-0.2091	0
MDM2	KDELR2	-0.2861	0
MDM2	KEAP1	-0.5146	1
MDM2	KRAS	-0.3876	0
MDM2	LATS2	0.9552	1
MDM2	MAP2K1	0.6942	1
MDM2	MAP2K3	-0.6187	1
MDM2	MAP2K4	0.4955	0
MDM2	MAP2K5	0.4243	0
MDM2	MAP2K6	0.1330	0
MDM2	MAP3K5	0.0173	0
MDM2	MAPK1	0.0093	0
MDM2	MAPK14	-0.4502	0
MDM2	MAPK3	-0.5397	1
MDM2	MAPK8	0.5814	1

GENE1	GENE2	R	Positive
MDM2	MAPK9	-0.8219	1
MDM2	MDM4	0.5693	1
MDM2	MSH2	-0.0112	0
MDM2	MTOR	0.5169	1
MDM2	NF1	0.2323	0
MDM2	NF2	-0.4721	0
MDM2	NFIC	0.4712	0
MDM2	NFKB2	0.0020	0
MDM2	NOTCH1	0.0239	0
MDM2	PIK3CA	-0.1715	0
MDM2	PIK3R1	0.5658	1
MDM2	PRKAA2	0.2797	0
MDM2	PTEN	-0.3174	0
MDM2	RASSF1	-0.3671	0
MDM2	RB1	-0.9621	1
MDM2	RELA	-0.1391	0
MDM2	ROS1	-0.3446	0
MDM2	SFN	0.9456	1
MDM2	SMAD1	-0.0110	0
MDM2	SMAD2	-0.4258	0
MDM2	SMAD4	-0.7937	1
MDM2	SMARCA4	0.2093	0
MDM2	SOX3	-0.0211	0
MDM2	SOX4	-0.2488	0
MDM2	STAT1	-0.0469	0
MDM2	STAT2	-0.3792	0
MDM2	STK11	-0.2161	0
MDM2	STK3	0.8212	1
MDM2	STK4	0.8588	1
MDM2	TCF3	0.4163	0
MDM2	TEAD2	-0.2579	0
MDM2	TERT	-0.0984	0
MDM2	TNFRSF1A	-0.4367	0
MDM2	TP53	0.5010	1
MDM2	TSC1	-0.2577	0
MDM2	WHSC1L1	-0.6550	1
MDM2	WT1	-0.5547	1
MDM2	YAP1	-0.3040	0
MDM2	YWHAZ	0.5214	1
MDM4	APC	-0.1799	0
MDM4	BAX	0.5891	1
MDM4	BCL2	-0.5321	1
MDM4	CDC42	-0.3126	0
MDM4	DVL1	0.1880	0
MDM4	E2F1	-0.7845	1
MDM4	GSK3B	0.0929	0
MDM4	JAK1	0.9020	1
MDM4	JAK2	0.3195	0
MDM4	KEAP1	-0.1561	0

GENE1	GENE2	R	Positive
MDM4	MAP2K1	0.4690	0
MDM4	MAP2K4	0.5756	1
MDM4	MAPK1	-0.6296	1
MDM4	MAPK3	-0.2671	0
MDM4	MAPK8	-0.1726	0
MDM4	MAPK9	-0.5902	1
MDM4	MTOR	0.6696	1
MDM4	NFIC	0.4150	0
MDM4	NFKB2	-0.7212	1
MDM4	NOTCH1	-0.5248	1
MDM4	PRKAA2	0.5478	1
MDM4	SMAD1	0.3654	0
MDM4	SMAD4	-0.5686	1
MDM4	STAT1	-0.3834	0
MDM4	STAT2	-0.2103	0
MDM4	STK3	0.5775	1
MDM4	STK4	0.1473	0
MDM4	TNFRSF1A	0.1249	0
MET	ABCB5	0.0917	0
MET	AKT1S1	-0.3430	0
MET	APC	-0.2790	0
MET	ARNT	0.3832	0
MET	BAX	-0.5100	1
MET	BCL2	0.1037	0
MET	BECN1	0.5783	1
MET	BRAF	0.4520	0
MET	CBLB	0.5130	1
MET	CBLC	-0.0793	0
MET	CCND2	0.6495	1
MET	CCNE1	-0.3766	0
MET	CD44	0.1332	0
MET	CDC42	-0.3293	0
MET	CDK4	-0.4814	0
MET	CDK6	0.1377	0
MET	CDKN2A	-0.3274	0
MET	CDKN2B	0.0251	0
MET	CDKN2C	0.3220	0
MET	CTNNB1	-0.2000	0
MET	DACH1	0.3103	0
MET	DVL1	-0.1755	0
MET	E2F1	0.6581	1
MET	EGFR	-0.5397	1
MET	EPHA2	-0.4681	0
MET	FGFR4	0.6019	1
MET	FOXO1	0.0709	0
MET	FZR1	0.3013	0
MET	GLIS2	0.6972	1
MET	GRM1	-0.2390	0
MET	GSK3B	-0.1851	0

GENE1	GENE2	R	Positive
MET	HGF	-0.1768	0
MET	HIF1A	0.1564	0
MET	HRAS	-0.2482	0
MET	IGF1R	0.1002	0
MET	JAK1	-0.1945	0
MET	JAK2	-0.1674	0
MET	KAT2A	0.6760	1
MET	KDELR2	0.3424	0
MET	KEAP1	0.7404	1
MET	KRAS	0.3031	0
MET	LATS2	-0.8048	1
MET	MAP2K1	0.0205	0
MET	MAP2K3	0.4575	0
MET	MAP2K4	-0.5407	1
MET	MAP2K5	-0.4501	0
MET	MAP2K6	-0.1766	0
MET	MAP3K5	0.5395	1
MET	MAPK1	-0.6789	1
MET	MAPK14	0.2935	0
MET	MAPK3	0.2830	0
MET	MAPK8	-0.3454	0
MET	MAPK9	0.8284	1
MET	MDM2	-0.6096	1
MET	MDM4	-0.0946	0
MET	MSH2	0.2267	0
MET	MTOR	-0.1717	0
MET	NF1	-0.4294	0
MET	NF2	0.8879	1
MET	NFIC	-0.7537	1
MET	NFKB2	-0.2714	0
MET	NOTCH1	0.1615	0
MET	PIK3CA	0.4392	0
MET	PIK3R1	-0.3005	0
MET	PRKAA2	0.2389	0
MET	PTEN	0.4526	0
MET	RASSF1	0.8144	1
MET	RB1	0.5364	1
MET	RELA	-0.6191	1
MET	ROS1	0.1081	0
MET	SFN	-0.5928	1
MET	SMAD1	0.4565	0
MET	SMAD2	0.1393	0
MET	SMAD4	0.4464	0
MET	SMARCA4	-0.1231	0
MET	SOX3	-0.4488	0
MET	SOX4	0.0240	0
MET	STAT1	-0.2039	0
MET	STAT2	-0.1175	0
MET	STK11	-0.0640	0

GENE1	GENE2	R	Positive
MET	STK3	-0.2416	0
MET	STK4	-0.7437	1
MET	TCF3	-0.3964	0
MET	TEAD2	0.0564	0
MET	TERT	0.5372	1
MET	TNFRSF1A	0.6605	1
MET	TP53	-0.6344	1
MET	TSC1	0.1280	0
MET	WHSC1L1	0.4459	0
MET	WT1	-0.2429	0
MET	YAP1	0.2345	0
MET	YWHAZ	-0.7151	1
MSH2	ABCB5	0.8764	1
MSH2	AKT1S1	-0.5612	1
MSH2	APC	-0.3166	0
MSH2	ARNT	0.2750	0
MSH2	BAX	0.4153	0
MSH2	BCL2	0.5346	1
MSH2	BECN1	0.7419	1
MSH2	CCND2	0.2343	0
MSH2	CCNE1	0.0059	0
MSH2	CD44	0.2035	0
MSH2	CDC42	-0.7199	1
MSH2	CDK4	-0.6206	1
MSH2	CDK6	-0.7485	1
MSH2	CDKN2C	-0.4452	0
MSH2	DACH1	-0.7983	1
MSH2	DVL1	0.6144	1
MSH2	E2F1	0.0410	0
MSH2	EPHA2	0.0468	0
MSH2	FGFR4	0.2610	0
MSH2	FZR1	-0.6176	1
MSH2	GLIS2	0.7479	1
MSH2	GRM1	-0.0013	0
MSH2	GSK3B	0.7933	1
MSH2	HGF	0.4751	0
MSH2	HIF1A	0.9489	1
MSH2	IGF1R	-0.7269	1
MSH2	JAK1	0.0681	0
MSH2	JAK2	-0.1964	0
MSH2	KAT2A	-0.2414	0
MSH2	KDELR2	0.6889	1
MSH2	KEAP1	0.3839	0
MSH2	MAP2K1	0.2148	0
MSH2	MAP2K3	-0.6428	1
MSH2	MAP2K4	0.5524	1
MSH2	MAP2K5	-0.1591	0
MSH2	MAP2K6	0.8887	1
MSH2	MAP3K5	0.3758	0

GENE1	GENE2	R	Positive
MSH2	MAPK1	-0.2676	0
MSH2	MAPK14	0.7996	1
MSH2	MAPK3	0.7932	1
MSH2	MAPK8	-0.4872	0
MSH2	MAPK9	0.2823	0
MSH2	MDM4	0.1352	0
MSH2	MTOR	0.0993	0
MSH2	NF1	-0.0850	0
MSH2	NFIC	0.2995	0
MSH2	NFKB2	-0.5293	1
MSH2	NOTCH1	-0.0231	0
MSH2	PRKAA2	0.7731	1
MSH2	RELA	-0.6057	1
MSH2	SFN	-0.3191	0
MSH2	SMAD1	0.8914	1
MSH2	SMAD4	0.1130	0
MSH2	SOX3	-0.7997	1
MSH2	SOX4	0.1947	0
MSH2	STAT1	0.7110	1
MSH2	STAT2	-0.3821	0
MSH2	STK3	0.4814	0
MSH2	STK4	-0.3230	0
MSH2	TEAD2	-0.4981	0
MSH2	TERT	0.1145	0
MSH2	TNFRSF1A	-0.2828	0
MSH2	YWHAZ	-0.4596	0
MYC	ABCB5	0.5416	1
MYC	AKT1	-0.2965	0
MYC	AKT1S1	-0.6225	1
MYC	APC	-0.5679	1
MYC	ARAF	-0.8552	1
MYC	ARNT	0.4920	0
MYC	AURKA	0.7321	1
MYC	BAX	0.1276	0
MYC	BCL2	0.0990	0
MYC	BECN1	0.7373	1
MYC	BRAF	0.4304	0
MYC	CBLB	0.8230	1
MYC	CBLC	0.0474	0
MYC	CCND2	0.5832	1
MYC	CCNE1	-0.4741	0
MYC	CD44	0.1434	0
MYC	CDC42	-0.5988	1
MYC	CDK4	-0.4748	0
MYC	CDK6	-0.4295	0
MYC	CDKN2A	-0.5109	1
MYC	CDKN2B	0.2888	0
MYC	CDKN2C	0.0721	0
MYC	CTNNB1	-0.5957	1

GENE1	GENE2	R	Positive
MYC	DACH1	-0.3852	0
MYC	DVL1	0.2110	0
MYC	E2F1	0.0160	0
MYC	EGFR	-0.4494	0
MYC	EPHA2	-0.4297	0
MYC	ERBB2	-0.6225	1
MYC	FGFR4	0.0369	0
MYC	FOXO1	-0.1055	0
MYC	FZR1	-0.3775	0
MYC	GLIS2	0.7176	1
MYC	GRM1	0.3565	0
MYC	GSK3B	0.3744	0
MYC	HGF	0.6665	1
MYC	HIF1A	0.6523	1
MYC	HRAS	0.2819	0
MYC	IGF1R	-0.5328	1
MYC	JAK1	0.2189	0
MYC	JAK2	-0.1052	0
MYC	KAT2A	0.3152	0
MYC	KDELR2	0.4168	0
MYC	KEAP1	0.6906	1
MYC	KRAS	0.4567	0
MYC	LATS2	-0.2014	0
MYC	MAP2K1	0.2410	0
MYC	MAP2K3	-0.3011	0
MYC	MAP2K4	0.3580	0
MYC	MAP2K5	0.0758	0
MYC	MAP2K6	0.5674	1
MYC	MAP3K5	0.8004	1
MYC	MAPK1	-0.5690	1
MYC	MAPK14	0.6982	1
MYC	MAPK3	0.5122	1
MYC	MAPK8	-0.4700	0
MYC	MAPK9	0.3355	0
MYC	MDM2	0.0032	0
MYC	MDM4	0.3693	0
MYC	MET	0.4973	0
MYC	MSH2	0.8329	1
MYC	MTOR	-0.0086	0
MYC	NF1	0.0897	0
MYC	NF2	0.8176	1
MYC	NFIC	-0.0216	0
MYC	NFKB2	-0.5704	1
MYC	NOTCH1	-0.2351	0
MYC	PDGFRA	0.1124	0
MYC	PIK3CA	0.3597	0
MYC	PIK3R1	0.4160	0
MYC	PRKAA2	0.9492	1
MYC	PTEN	0.5140	1

GENE1	GENE2	R	Positive
MYC	RAF1	0.6436	1
MYC	RASSF1	0.0516	0
MYC	RB1	-0.0084	0
MYC	RELA	-0.8910	1
MYC	ROS1	0.2404	0
MYC	SFN	-0.2343	0
MYC	SMAD1	0.9907	1
MYC	SMAD2	0.4621	0
MYC	SMAD4	0.2136	0
MYC	SMARCA4	0.7308	1
MYC	SOX3	-0.9888	1
MYC	SOX4	-0.2692	0
MYC	STAT1	0.2274	0
MYC	STAT2	-0.6687	1
MYC	STK11	0.5146	1
MYC	STK3	0.4211	0
MYC	STK4	-0.4453	0
MYC	TCF3	0.3942	0
MYC	TEAD2	-0.7024	1
MYC	TERT	0.2072	0
MYC	TNFRSF1A	-0.1043	0
MYC	TP53	0.0861	0
MYC	TSC1	0.6092	1
MYC	WHSC1L1	0.3258	0
MYC	WT1	-0.4522	0
MYC	YAP1	0.6382	1
MYC	YWHAZ	-0.6970	1
NF1	ABCB5	-0.2548	0
NF1	AKT1S1	-0.2273	0
NF1	APC	0.2532	0
NF1	ARNT	-0.2020	0
NF1	BAX	0.2361	0
NF1	BCL2	-0.3196	0
NF1	BECN1	0.0663	0
NF1	CCND2	-0.3765	0
NF1	CCNE1	-0.1358	0
NF1	CD44	-0.1259	0
NF1	CDC42	-0.1454	0
NF1	CDK4	0.0533	0
NF1	CDK6	0.1824	0
NF1	CDKN2C	0.5289	1
NF1	DACH1	-0.0320	0
NF1	DVL1	-0.3440	0
NF1	E2F1	-0.5418	1
NF1	EPHA2	-0.4062	0
NF1	FGFR4	-0.7546	1
NF1	FZR1	-0.5491	1
NF1	GLIS2	-0.1879	0
NF1	GRM1	0.6998	1
NF1	GSK3B	-0.0093	0
NF1	HGF	0.4686	0
NF1	HIF1A	-0.1446	0
NF1	IGF1R	-0.2119	0
NF1	JAK1	0.7401	1
NF1	JAK2	-0.3293	0
NF1	KAT2A	0.1281	0
NF1	KDELR2	-0.6753	1
NF1	KEAP1	0.0015	0
NF1	MAP2K1	-0.3835	0
NF1	MAP2K3	0.0896	0
NF1	MAP2K4	0.5776	1
NF1	MAP2K5	0.7154	1
NF1	MAP2K6	0.1278	0
NF1	MAP3K5	0.3818	0
NF1	MAPK1	0.1008	0
NF1	MAPK14	0.2194	0
NF1	MAPK3	-0.1217	0
NF1	MAPK8	-0.3656	0
NF1	MAPK9	-0.5367	1
NF1	MDM4	0.5439	1
NF1	MTOR	-0.0701	0
NF1	NFIC	0.5167	1
NF1	NFKB2	-0.2721	0
NF1	NOTCH1	-0.9493	1
NF1	PRKAA2	0.2918	0
NF1	RELA	0.1501	0
NF1	SFN	0.1761	0
NF1	SMAD1	0.0782	0
NF1	SMAD4	0.0661	0
NF1	SOX3	-0.2223	0
NF1	SOX4	-0.7231	1
NF1	STAT1	-0.3199	0
NF1	STAT2	-0.2077	0
NF1	STK3	-0.0574	0
NF1	STK4	-0.0145	0
NF1	TEAD2	-0.7642	1
NF1	TERT	-0.3013	0
NF1	TNFRSF1A	-0.3651	0
NF1	YWHAZ	-0.2123	0
NF2	ABCB5	0.3738	0
NF2	AKT1S1	-0.6073	1
NF2	APC	-0.3355	0
NF2	ARNT	0.4034	0
NF2	BAX	-0.2207	0
NF2	BCL2	0.2045	0
NF2	BECN1	0.8298	1
NF2	CCND2	0.6007	1
NF2	CCNE1	-0.4012	0

GENE1	GENE2	R	Positive
NF2	CD44	0.1458	0
NF2	CDC42	-0.6221	1
NF2	CDK4	-0.6523	1
NF2	CDK6	-0.1425	0
NF2	CDKN2A	-0.5095	1
NF2	CDKN2B	0.2456	0
NF2	CDKN2C	0.2551	0
NF2	DACH1	-0.0472	0
NF2	DVL1	0.0099	0
NF2	E2F1	0.4513	0
NF2	EPHA2	-0.5272	1
NF2	FGFR4	0.4428	0
NF2	FZR1	-0.1076	0
NF2	GLIS2	0.8754	1
NF2	GRM1	-0.0179	0
NF2	GSK3B	0.1448	0
NF2	HGF	0.1675	0
NF2	HIF1A	0.4836	0
NF2	IGF1R	-0.2672	0
NF2	JAK1	0.0552	0
NF2	JAK2	-0.2868	0
NF2	KAT2A	0.5572	1
NF2	KDELR2	0.4055	0
NF2	KEAP1	0.8179	1
NF2	LATS2	-0.6753	1
NF2	MAP2K1	0.0373	0
NF2	MAP2K3	0.1674	0
NF2	MAP2K4	-0.1129	0
NF2	MAP2K5	-0.2881	0
NF2	MAP2K6	0.2294	0
NF2	MAP3K5	0.7523	1
NF2	MAPK1	-0.7186	1
NF2	MAPK14	0.6298	1
NF2	MAPK3	0.5270	1
NF2	MAPK8	-0.5896	1
NF2	MAPK9	0.7107	1
NF2	MDM4	0.1339	0
NF2	MSH2	0.5875	1
NF2	MTOR	-0.1091	0
NF2	NF1	-0.1678	0
NF2	NFIC	-0.4279	0
NF2	NFKB2	-0.5409	1
NF2	NOTCH1	-0.1025	0
NF2	PIK3R1	0.0512	0
NF2	PRKAA2	0.6161	1
NF2	PTEN	0.5614	1
NF2	RASSF1	0.5158	1
NF2	RB1	0.4245	0
NF2	RELA	-0.7846	1
NF2	SFN	-0.5963	1
NF2	SMAD1	0.8002	1
NF2	SMAD2	0.4457	0
NF2	SMAD4	0.4404	0
NF2	SMARCA4	0.3422	0
NF2	SOX3	-0.7946	1
NF2	SOX4	-0.0920	0
NF2	STAT1	0.0079	0
NF2	STAT2	-0.3241	0
NF2	STK11	0.2852	0
NF2	STK3	-0.0129	0
NF2	STK4	-0.7916	1
NF2	TEAD2	-0.3522	0
NF2	TERT	0.4732	0
NF2	TNFRSF1A	0.3977	0
NF2	TP53	-0.3317	0
NF2	TSC1	0.4149	0
NF2	WT1	-0.3204	0
NF2	YWHAZ	-0.8856	1
NFIC	APC	0.3578	0
NFIC	BAX	0.8823	1
NFIC	BCL2	0.2111	0
NFIC	CDC42	-0.3569	0
NFIC	DVL1	0.5598	1
NFIC	E2F1	-0.6858	1
NFIC	GSK3B	0.6930	1
NFIC	JAK1	0.5672	1
NFIC	JAK2	-0.0845	0
NFIC	MAP2K1	0.0464	0
NFIC	MAP2K4	0.9204	1
NFIC	MAPK1	0.2556	0
NFIC	MAPK3	0.2070	0
NFIC	MAPK8	-0.2219	0
NFIC	MAPK9	-0.7042	1
NFIC	MTOR	0.4582	0
NFIC	NFKB2	-0.3816	0
NFIC	NOTCH1	-0.3924	0
NFIC	PRKAA2	0.1812	0
NFIC	SMAD1	0.0725	0
NFIC	SMAD4	-0.4440	0
NFIC	STAT1	0.4298	0
NFIC	STAT2	0.1588	0
NFIC	TNFRSF1A	-0.4971	0
PDGFRA	ABCB5	0.1827	0
PDGFRA	AKT1S1	-0.5686	1
PDGFRA	APC	-0.0353	0
PDGFRA	ARAF	0.0053	0
PDGFRA	ARNT	-0.5526	1
PDGFRA	AURKA	-0.1748	0

GENE1	GENE2	R	Positive
PDGFRA	BAX	0.7701	1
PDGFRA	BCL2	-0.4669	0
PDGFRA	BECN1	-0.1635	0
PDGFRA	BRAF	0.4743	0
PDGFRA	CBLB	0.2165	0
PDGFRA	CBLC	0.3942	0
PDGFRA	CCND2	-0.2086	0
PDGFRA	CCNE1	-0.4643	0
PDGFRA	CD44	-0.8510	1
PDGFRA	CDC42	-0.2044	0
PDGFRA	CDK4	0.4055	0
PDGFRA	CDK6	-0.4603	0
PDGFRA	CDKN2A	-0.1527	0
PDGFRA	CDKN2B	-0.3431	0
PDGFRA	CDKN2C	0.2361	0
PDGFRA	CTNNB1	-0.5487	1
PDGFRA	DACH1	-0.2549	0
PDGFRA	DVL1	0.3611	0
PDGFRA	E2F1	-0.9136	1
PDGFRA	EGFR	-0.2226	0
PDGFRA	EPHA2	-0.4945	0
PDGFRA	ERBB2	0.1822	0
PDGFRA	FGFR4	-0.6070	1
PDGFRA	FOXO1	0.0061	0
PDGFRA	FZR1	-0.3051	0
PDGFRA	GLIS2	-0.2931	0
PDGFRA	GRM1	0.6396	1
PDGFRA	GSK3B	0.1916	0
PDGFRA	HGF	0.4386	0
PDGFRA	HIF1A	0.0325	0
PDGFRA	HRAS	-0.2581	0
PDGFRA	IGF1R	-0.6297	1
PDGFRA	JAK1	0.8592	1
PDGFRA	JAK2	0.4237	0
PDGFRA	KAT2A	-0.0428	0
PDGFRA	KDELR2	-0.5695	1
PDGFRA	KEAP1	-0.4929	0
PDGFRA	KRAS	-0.2273	0
PDGFRA	LATS2	0.6893	1
PDGFRA	MAP2K1	0.5261	1
PDGFRA	MAP2K3	-0.3926	0
PDGFRA	MAP2K4	0.6569	1
PDGFRA	MAP2K5	0.1341	0
PDGFRA	MAP2K6	0.2495	0
PDGFRA	MAP3K5	0.3392	0
PDGFRA	MAPK1	-0.4185	0
PDGFRA	MAPK14	-0.1751	0
PDGFRA	MAPK3	-0.3355	0
PDGFRA	MAPK8	-0.0082	0
PDGFRA	MAPK9	-0.7987	1
PDGFRA	MDM2	0.7104	1
PDGFRA	MDM4	0.9330	1
PDGFRA	MET	-0.3787	0
PDGFRA	MSH2	0.0396	0
PDGFRA	MTOR	0.8100	1
PDGFRA	NF1	0.4863	0
PDGFRA	NF2	-0.1773	0
PDGFRA	NFIC	0.6160	1
PDGFRA	NFKB2	-0.6118	1
PDGFRA	NOTCH1	-0.3898	0
PDGFRA	PIK3CA	-0.2453	0
PDGFRA	PIK3R1	0.8549	1
PDGFRA	PRKAA2	0.3365	0
PDGFRA	PTEN	-0.1865	0
PDGFRA	RASSF1	-0.1508	0
PDGFRA	RB1	-0.7146	1
PDGFRA	RELA	-0.1273	0
PDGFRA	ROS1	-0.2231	0
PDGFRA	SFN	0.6068	1
PDGFRA	SMAD1	0.1361	0
PDGFRA	SMAD2	0.0215	0
PDGFRA	SMAD4	-0.7793	1
PDGFRA	SMARCA4	0.3897	0
PDGFRA	SOX3	-0.2059	0
PDGFRA	SOX4	-0.2284	0
PDGFRA	STAT1	-0.2488	0
PDGFRA	STAT2	-0.0123	0
PDGFRA	STK11	-0.1404	0
PDGFRA	STK3	0.6472	1
PDGFRA	STK4	0.4045	0
PDGFRA	TCF3	0.6245	1
PDGFRA	TEAD2	-0.4718	0
PDGFRA	TERT	0.4060	0
PDGFRA	TNFRSF1A	0.0297	0
PDGFRA	TP53	0.4808	0
PDGFRA	TSC1	-0.4102	0
PDGFRA	WHSC1L1	-0.5784	1
PDGFRA	WT1	-0.6635	1
PDGFRA	YAP1	0.1889	0
PDGFRA	YWHAZ	-0.0224	0
PIK3CA	ABCB5	0.7517	1
PIK3CA	AKT1S1	-0.2437	0
PIK3CA	APC	-0.2818	0
PIK3CA	ARNT	0.1496	0
PIK3CA	BAX	0.1672	0
PIK3CA	BCL2	0.5668	1
PIK3CA	BECN1	0.3714	0
PIK3CA	CBLB	0.2685	0

GENE1	GENE2	R	Positive
PIK3CA	CCND2	0.3283	0
PIK3CA	CCNE1	0.1206	0
PIK3CA	CD44	0.0642	0
PIK3CA	CDC42	-0.3704	0
PIK3CA	CDK4	-0.3926	0
PIK3CA	CDK6	-0.6422	1
PIK3CA	CDKN2A	0.5317	1
PIK3CA	CDKN2B	-0.3848	0
PIK3CA	CDKN2C	-0.6604	1
PIK3CA	CTNNB1	-0.5813	1
PIK3CA	DACH1	-0.4717	0
PIK3CA	DVL1	0.7055	1
PIK3CA	E2F1	0.3789	0
PIK3CA	EGFR	0.3504	0
PIK3CA	EPHA2	0.3019	0
PIK3CA	FGFR4	0.7536	1
PIK3CA	FZR1	0.0314	0
PIK3CA	GLIS2	0.5656	1
PIK3CA	GRM1	-0.5705	1
PIK3CA	GSK3B	0.5166	1
PIK3CA	HGF	-0.1589	0
PIK3CA	HIF1A	0.6741	1
PIK3CA	IGF1R	-0.3440	0
PIK3CA	JAK1	-0.4313	0
PIK3CA	JAK2	0.1570	0
PIK3CA	KAT2A	-0.2808	0
PIK3CA	KDELR2	0.8511	1
PIK3CA	KEAP1	0.1069	0
PIK3CA	LATS2	-0.3597	0
PIK3CA	MAP2K1	0.4611	0
PIK3CA	MAP2K3	-0.4557	0
PIK3CA	MAP2K4	-0.0828	0
PIK3CA	MAP2K5	-0.7453	1
PIK3CA	MAP2K6	0.4339	0
PIK3CA	MAP3K5	-0.0957	0
PIK3CA	MAPK1	-0.2964	0
PIK3CA	MAPK14	0.2618	0
PIK3CA	MAPK3	0.5405	1
PIK3CA	MAPK8	-0.0366	0
PIK3CA	MAPK9	0.5076	1
PIK3CA	MDM4	-0.2609	0
PIK3CA	MSH2	0.6179	1
PIK3CA	MTOR	0.2573	0
PIK3CA	NF1	-0.8165	1
PIK3CA	NF2	0.4405	0
PIK3CA	NFIC	-0.1522	0
PIK3CA	NFKB2	-0.1990	0
PIK3CA	NOTCH1	0.7086	1
PIK3CA	PIK3R1	0.1118	0

GENE1	GENE2	R	Positive
PIK3CA	PRKAA2	0.1736	0
PIK3CA	PTEN	-0.4298	0
PIK3CA	RASSF1	0.2766	0
PIK3CA	RB1	0.3733	0
PIK3CA	RELA	-0.4132	0
PIK3CA	ROS1	-0.6504	1
PIK3CA	SFN	-0.3100	0
PIK3CA	SMAD1	0.4164	0
PIK3CA	SMAD2	0.3041	0
PIK3CA	SMAD4	-0.0927	0
PIK3CA	SMARCA4	0.1689	0
PIK3CA	SOX3	-0.2501	0
PIK3CA	SOX4	0.7467	1
PIK3CA	STAT1	0.6370	1
PIK3CA	STAT2	0.0713	0
PIK3CA	STK11	-0.2684	0
PIK3CA	STK3	0.3460	0
PIK3CA	STK4	-0.1650	0
PIK3CA	TEAD2	0.3302	0
PIK3CA	TERT	0.4239	0
PIK3CA	TNFRSF1A	0.2163	0
PIK3CA	TP53	0.2579	0
PIK3CA	TSC1	0.4795	0
PIK3CA	WT1	-0.3366	0
PIK3CA	YWHAZ	-0.1141	0
PIK3R1	ABCB5	0.6397	1
PIK3R1	AKT1S1	-0.7416	1
PIK3R1	APC	-0.0514	0
PIK3R1	ARNT	-0.4402	0
PIK3R1	BAX	0.9291	1
PIK3R1	BCL2	-0.0176	0
PIK3R1	BECN1	0.2215	0
PIK3R1	CCND2	-0.2117	0
PIK3R1	CCNE1	-0.2363	0
PIK3R1	CD44	-0.6210	1
PIK3R1	CDC42	-0.5631	1
PIK3R1	CDK4	-0.0052	0
PIK3R1	CDK6	-0.8053	1
PIK3R1	CDKN2C	-0.1204	0
PIK3R1	DACH1	-0.6952	1
PIK3R1	DVL1	0.6830	1
PIK3R1	E2F1	-0.7489	1
PIK3R1	EPHA2	-0.2816	0
PIK3R1	FGFR4	-0.3371	0
PIK3R1	FZR1	-0.6416	1
PIK3R1	GLIS2	0.1099	0
PIK3R1	GRM1	0.4403	0
PIK3R1	GSK3B	0.6487	1
PIK3R1	HGF	0.5160	1

GENE1	GENE2	R	Positive
PIK3R1	HIF1A	0.5405	1
PIK3R1	IGF1R	-0.9297	1
PIK3R1	JAK1	0.7535	1
PIK3R1	JAK2	0.1972	0
PIK3R1	KAT2A	-0.3006	0
PIK3R1	KDELR2	-0.1262	0
PIK3R1	KEAP1	-0.3316	0
PIK3R1	MAP2K1	0.4913	0
PIK3R1	MAP2K3	-0.6944	1
PIK3R1	MAP2K4	0.8781	1
PIK3R1	MAP2K5	-0.0216	0
PIK3R1	MAP2K6	0.7092	1
PIK3R1	MAP3K5	0.3464	0
PIK3R1	MAPK1	-0.3890	0
PIK3R1	MAPK14	0.2658	0
PIK3R1	MAPK3	0.1689	0
PIK3R1	MAPK8	-0.2787	0
PIK3R1	MAPK9	-0.5704	1
PIK3R1	MDM4	0.7981	1
PIK3R1	MSH2	0.5238	1
PIK3R1	MTOR	0.7585	1
PIK3R1	NF1	0.3657	0
PIK3R1	NFIC	0.7698	1
PIK3R1	NFKB2	-0.7723	1
PIK3R1	NOTCH1	-0.3300	0
PIK3R1	PRKAA2	0.5763	1
PIK3R1	PTEN	-0.2284	0
PIK3R1	RB1	-0.4722	0
PIK3R1	RELA	-0.2735	0
PIK3R1	SFN	0.3178	0
PIK3R1	SMAD1	0.4845	0
PIK3R1	SMAD2	0.4402	0
PIK3R1	SMAD4	-0.6186	1
PIK3R1	SOX3	-0.4818	0
PIK3R1	SOX4	0.0146	0
PIK3R1	STAT1	0.2338	0
PIK3R1	STAT2	-0.0733	0
PIK3R1	STK3	0.7486	1
PIK3R1	STK4	0.1999	0
PIK3R1	TEAD2	-0.5879	1
PIK3R1	TERT	0.3736	0
PIK3R1	TNFRSF1A	-0.1421	0
PIK3R1	WT1	-0.6058	1
PIK3R1	YWHAZ	-0.1886	0
PTEN	ABCB5	-0.3544	0
PTEN	AKT1S1	-0.1385	0
PTEN	APC	-0.2308	0
PTEN	ARNT	0.5458	1
PTEN	BAX	-0.5327	1
PTEN	BCL2	-0.2603	0
PTEN	BECN1	0.4448	0
PTEN	CCND2	0.4249	0
PTEN	CCNE1	-0.4284	0
PTEN	CD44	0.3885	0
PTEN	CDC42	-0.1246	0
PTEN	CDK4	-0.2884	0
PTEN	CDK6	0.5147	1
PTEN	CDKN2C	0.7094	1
PTEN	DACH1	0.3814	0
PTEN	DVL1	-0.7065	1
PTEN	E2F1	0.2276	0
PTEN	EPHA2	-0.5896	1
PTEN	FGFR4	-0.2374	0
PTEN	FZR1	-0.0687	0
PTEN	GLIS2	0.3406	0
PTEN	GRM1	0.4690	0
PTEN	GSK3B	-0.3614	0
PTEN	HGF	0.3948	0
PTEN	HIF1A	-0.1274	0
PTEN	IGF1R	0.2092	0
PTEN	JAK1	0.1805	0
PTEN	JAK2	-0.4299	0
PTEN	KAT2A	0.7583	1
PTEN	KDELR2	-0.1744	0
PTEN	KEAP1	0.8390	1
PTEN	MAP2K1	-0.4366	0
PTEN	MAP2K3	0.5281	1
PTEN	MAP2K4	-0.1103	0
PTEN	MAP2K5	0.5318	1
PTEN	MAP2K6	-0.1657	0
PTEN	MAP3K5	0.7376	1
PTEN	MAPK1	-0.2349	0
PTEN	MAPK14	0.4147	0
PTEN	MAPK3	0.0760	0
PTEN	MAPK8	-0.4210	0
PTEN	MAPK9	0.3560	0
PTEN	MDM4	0.1401	0
PTEN	MSH2	0.0765	0
PTEN	MTOR	-0.5955	1
PTEN	NF1	0.4909	0
PTEN	NFIC	-0.3784	0
PTEN	NFKB2	-0.1012	0
PTEN	NOTCH1	-0.6274	1
PTEN	PRKAA2	0.4755	0
PTEN	RELA	-0.4306	0
PTEN	SFN	-0.3108	0
PTEN	SMAD1	0.4302	0
PTEN	SMAD2	0.0816	0

GENE1	GENE2	R	Positive
PTEN	SMAD4	0.6784	1
PTEN	SOX3	-0.5572	1
PTEN	SOX4	-0.7986	1
PTEN	STAT1	-0.4291	0
PTEN	STAT2	-0.5768	1
PTEN	STK3	-0.3465	0
PTEN	STK4	-0.5815	1
PTEN	TEAD2	-0.6128	1
PTEN	TERT	-0.1880	0
PTEN	TNFRSF1A	-0.0328	0
PTEN	YWHAZ	-0.6454	1
RAF1	ABCB5	0.6804	1
RAF1	AKT1S1	-0.2126	0
RAF1	APC	-0.7335	1
RAF1	ARAF	-0.3554	0
RAF1	ARNT	0.4379	0
RAF1	AURKA	0.4226	0
RAF1	BAX	0.3552	0
RAF1	BCL2	0.1413	0
RAF1	BECN1	0.2016	0
RAF1	BRAF	-0.2590	0
RAF1	CBLB	0.2604	0
RAF1	CBLC	-0.2898	0
RAF1	CCND2	0.5314	1
RAF1	CCNE1	-0.2502	0
RAF1	CD44	0.0806	0
RAF1	CDC42	-0.1886	0
RAF1	CDK4	-0.0459	0
RAF1	CDK6	-0.7283	1
RAF1	CDKN2A	0.2715	0
RAF1	CDKN2B	-0.2850	0
RAF1	CDKN2C	-0.5600	1
RAF1	CTNNB1	-0.7995	1
RAF1	DACH1	-0.5803	1
RAF1	DVL1	0.6390	1
RAF1	E2F1	-0.1520	0
RAF1	EGFR	0.1228	0
RAF1	EPHA2	0.1643	0
RAF1	ERBB2	-0.3994	0
RAF1	FGFR4	0.0902	0
RAF1	FOXO1	-0.5334	1
RAF1	FZR1	-0.1065	0
RAF1	GLIS2	0.3011	0
RAF1	GRM1	0.1145	0
RAF1	GSK3B	0.4801	0
RAF1	HGF	0.5692	1
RAF1	HIF1A	0.6366	1
RAF1	HRAS	0.2888	0
RAF1	IGF1R	-0.4693	0
RAF1	JAK1	-0.1907	0
RAF1	JAK2	0.4403	0
RAF1	KAT2A	-0.2261	0
RAF1	KDELR2	0.6875	1
RAF1	KEAP1	0.1474	0
RAF1	KRAS	0.5019	1
RAF1	LATS2	0.2047	0
RAF1	MAP2K1	0.6927	1
RAF1	MAP2K3	-0.7873	1
RAF1	MAP2K4	0.3318	0
RAF1	MAP2K5	-0.0984	0
RAF1	MAP2K6	0.5957	1
RAF1	MAP3K5	0.1876	0
RAF1	MAPK1	-0.2325	0
RAF1	MAPK14	0.2442	0
RAF1	MAPK3	0.3078	0
RAF1	MAPK8	0.1739	0
RAF1	MAPK9	0.0812	0
RAF1	MDM2	0.4219	0
RAF1	MDM4	0.1196	0
RAF1	MET	0.1006	0
RAF1	MSH2	0.7641	1
RAF1	MTOR	0.2458	0
RAF1	NF1	-0.3892	0
RAF1	NF2	0.3139	0
RAF1	NFIC	0.1151	0
RAF1	NFKB2	-0.1730	0
RAF1	NOTCH1	0.4263	0
RAF1	PDGFRA	0.1054	0
RAF1	PIK3CA	0.6973	1
RAF1	PIK3R1	0.4236	0
RAF1	PRKAA2	0.6529	1
RAF1	PTEN	-0.1994	0
RAF1	RASSF1	-0.1236	0
RAF1	RB1	-0.2656	0
RAF1	RELA	-0.6862	1
RAF1	ROS1	-0.4339	0
RAF1	SFN	0.2076	0
RAF1	SMAD1	0.6608	1
RAF1	SMAD2	0.1140	0
RAF1	SMAD4	-0.2759	0
RAF1	SMARCA4	0.5199	1
RAF1	SOX3	-0.5587	1
RAF1	SOX4	0.2101	0
RAF1	STAT1	0.5892	1
RAF1	STAT2	-0.5528	1
RAF1	STK11	0.0270	0
RAF1	STK3	0.8036	1
RAF1	STK4	0.2145	0

GENE1	GENE2	R	Positive
RAF1	TCF3	0.0093	0
RAF1	TEAD2	-0.1997	0
RAF1	TERT	0.0792	0
RAF1	TNFRSF1A	-0.3307	0
RAF1	TP53	0.4687	0
RAF1	TSC1	0.5636	1
RAF1	WHSC1L1	0.3198	0
RAF1	WT1	-0.5564	1
RAF1	YAP1	0.2133	0
RAF1	YWHAZ	0.0241	0
RASSF1	ABCB5	-0.2025	0
RASSF1	AKT1S1	-0.1775	0
RASSF1	APC	-0.1956	0
RASSF1	ARNT	0.0697	0
RASSF1	BAX	-0.4673	0
RASSF1	BCL2	-0.2457	0
RASSF1	BECN1	0.0763	0
RASSF1	CCND2	0.5302	1
RASSF1	CCNE1	-0.4852	0
RASSF1	CD44	-0.2664	0
RASSF1	CDC42	0.0283	0
RASSF1	CDK4	0.0047	0
RASSF1	CDK6	0.2686	0
RASSF1	CDKN2A	-0.0840	0
RASSF1	CDKN2C	0.4377	0
RASSF1	DACH1	0.6061	1
RASSF1	DVL1	-0.2188	0
RASSF1	E2F1	0.4227	0
RASSF1	EPHA2	-0.5125	1
RASSF1	FGFR4	0.4498	0
RASSF1	FZR1	0.6611	1
RASSF1	GLIS2	0.2198	0
RASSF1	GRM1	-0.2132	0
RASSF1	GSK3B	-0.4971	0
RASSF1	HGF	-0.3971	0
RASSF1	HIF1A	-0.2746	0
RASSF1	IGF1R	0.2904	0
RASSF1	JAK1	-0.1336	0
RASSF1	JAK2	0.2176	0
RASSF1	KAT2A	0.6908	1
RASSF1	KDELR2	-0.0231	0
RASSF1	KEAP1	0.3021	0
RASSF1	MAP2K1	0.2419	0
RASSF1	MAP2K3	0.5708	1
RASSF1	MAP2K4	-0.7254	1
RASSF1	MAP2K5	-0.5300	1
RASSF1	MAP2K6	-0.5624	1
RASSF1	MAP3K5	0.3119	0
RASSF1	MAPK1	-0.6915	1

GENE1	GENE2	R	Positive
RASSF1	MAPK14	-0.2744	0
RASSF1	MAPK3	-0.2270	0
RASSF1	MAPK8	0.0453	0
RASSF1	MAPK9	0.5008	1
RASSF1	MDM4	0.0192	0
RASSF1	MSH2	-0.2534	0
RASSF1	MTOR	0.1270	0
RASSF1	NF1	-0.5071	1
RASSF1	NFIC	-0.7897	1
RASSF1	NFKB2	-0.1287	0
RASSF1	NOTCH1	0.3324	0
RASSF1	PIK3R1	-0.3391	0
RASSF1	PRKAA2	-0.1406	0
RASSF1	PTEN	0.1432	0
RASSF1	RB1	0.2757	0
RASSF1	RELA	-0.3668	0
RASSF1	SFN	-0.2162	0
RASSF1	SMAD1	-0.0028	0
RASSF1	SMAD2	-0.2834	0
RASSF1	SMAD4	0.0346	0
RASSF1	SMARCA4	-0.5394	1
RASSF1	SOX3	-0.0146	0
RASSF1	SOX4	0.0815	0
RASSF1	STAT1	-0.5385	1
RASSF1	STAT2	0.1528	0
RASSF1	STK3	-0.2012	0
RASSF1	STK4	-0.3678	0
RASSF1	TEAD2	0.3828	0
RASSF1	TERT	0.7015	1
RASSF1	TNFRSF1A	0.8946	1
RASSF1	TP53	-0.7071	1
RASSF1	TSC1	-0.4338	0
RASSF1	WT1	-0.3978	0
RASSF1	YWHAZ	-0.3692	0
RB1	ABCB5	0.1917	0
RB1	AKT1S1	-0.0476	0
RB1	APC	0.4804	0
RB1	ARNT	0.0132	0
RB1	BAX	-0.3972	0
RB1	BCL2	0.7677	1
RB1	BECN1	0.5906	1
RB1	CCND2	-0.1383	0
RB1	CCNE1	0.5652	1
RB1	CD44	0.4514	0
RB1	CDC42	-0.4209	0
RB1	CDK4	-0.8113	1
RB1	CDK6	0.1531	0
RB1	CDKN2C	-0.1537	0
RB1	DACH1	-0.0198	0

GENE1	GENE2	R	Positive
RB1	DVL1	-0.0497	0
RB1	E2F1	0.8896	1
RB1	EPHA2	0.2284	0
RB1	FGFR4	0.8628	1
RB1	FZR1	-0.1058	0
RB1	GLIS2	0.6689	1
RB1	GRM1	-0.8109	1
RB1	GSK3B	0.2000	0
RB1	HGF	-0.6762	1
RB1	HIF1A	0.3059	0
RB1	IGF1R	0.1313	0
RB1	JAK1	-0.3995	0
RB1	JAK2	-0.7363	1
RB1	KAT2A	-0.0256	0
RB1	KDELR2	0.4647	0
RB1	KEAP1	0.3933	0
RB1	MAP2K1	-0.5997	1
RB1	MAP2K3	0.4050	0
RB1	MAP2K4	-0.4029	0
RB1	MAP2K5	-0.5708	1
RB1	MAP2K6	0.0433	0
RB1	MAP3K5	-0.1647	0
RB1	MAPK1	0.0495	0
RB1	MAPK14	0.4937	0
RB1	MAPK3	0.6711	1
RB1	MAPK8	-0.5613	1
RB1	MAPK9	0.8139	1
RB1	MDM4	-0.6375	1
RB1	MSH2	0.1492	0
RB1	MTOR	-0.4108	0
RB1	NF1	-0.3744	0
RB1	NFIC	-0.3481	0
RB1	NFKB2	-0.0219	0
RB1	NOTCH1	0.1323	0
RB1	PRKAA2	-0.2933	0
RB1	PTEN	0.0983	0
RB1	RELA	0.1706	0
RB1	SFN	-0.9543	1
RB1	SMAD1	0.0353	0
RB1	SMAD2	0.5248	1
RB1	SMAD4	0.6962	1
RB1	SOX3	0.0455	0
RB1	SOX4	0.4812	0
RB1	STAT1	0.2865	0
RB1	STAT2	0.4599	0
RB1	STK3	-0.7022	1
RB1	STK4	-0.7872	1
RB1	TEAD2	0.3450	0
RB1	TERT	0.1191	0

GENE1	GENE2	R	Positive
RB1	TNFRSF1A	0.3777	0
RB1	YWHAZ	-0.4293	0
RELA	APC	0.8095	1
RELA	BAX	0.0302	0
RELA	BCL2	0.2007	0
RELA	CDC42	0.3005	0
RELA	DVL1	-0.1280	0
RELA	E2F1	-0.0021	0
RELA	GSK3B	-0.0544	0
RELA	JAK1	-0.0831	0
RELA	JAK2	-0.2695	0
RELA	KEAP1	-0.6355	1
RELA	MAP2K1	-0.5237	1
RELA	MAP2K4	-0.0610	0
RELA	MAPK1	0.7186	1
RELA	MAPK3	-0.1702	0
RELA	MAPK8	0.1194	0
RELA	MAPK9	-0.3296	0
RELA	MDM4	-0.3866	0
RELA	MTOR	-0.0929	0
RELA	NFIC	0.3106	0
RELA	NFKB2	0.4155	0
RELA	NOTCH1	-0.0393	0
RELA	PRKAA2	-0.8364	1
RELA	SMAD1	-0.8436	1
RELA	SMAD4	-0.0228	0
RELA	STAT1	0.0169	0
RELA	STAT2	0.7116	1
RELA	STK3	-0.5130	1
RELA	STK4	0.2563	0
RELA	TNFRSF1A	-0.0999	0
ROS1	ABCB5	-0.4307	0
ROS1	AKT1S1	0.0332	0
ROS1	APC	0.1063	0
ROS1	ARNT	0.3559	0
ROS1	BAX	-0.4529	0
ROS1	BCL2	-0.1161	0
ROS1	BECN1	0.3402	0
ROS1	CCND2	0.0255	0
ROS1	CCNE1	-0.0710	0
ROS1	CD44	0.4829	0
ROS1	CDC42	-0.0752	0
ROS1	CDK4	-0.3011	0
ROS1	CDK6	0.5911	1
ROS1	CDKN2A	-0.8492	1
ROS1	CDKN2B	0.8832	1
ROS1	CDKN2C	0.5994	1
ROS1	DACH1	0.2863	0
ROS1	DVL1	-0.7422	1

GENE1	GENE2	R	Positive
ROS1	E2F1	0.1745	0
ROS1	EPHA2	-0.3358	0
ROS1	FGFR4	-0.3301	0
ROS1	FZR1	-0.2666	0
ROS1	GLIS2	0.1769	0
ROS1	GRM1	0.3865	0
ROS1	GSK3B	-0.2690	0
ROS1	HGF	0.2765	0
ROS1	HIF1A	-0.1789	0
ROS1	IGF1R	0.2483	0
ROS1	JAK1	0.1997	0
ROS1	JAK2	-0.6420	1
ROS1	KAT2A	0.4721	0
ROS1	KDELR2	-0.2902	0
ROS1	KEAP1	0.6245	1
ROS1	LATS2	-0.2476	0
ROS1	MAP2K1	-0.7283	1
ROS1	MAP2K3	0.5151	1
ROS1	MAP2K4	0.0266	0
ROS1	MAP2K5	0.6590	1
ROS1	MAP2K6	-0.1166	0
ROS1	MAP3K5	0.4506	0
ROS1	MAPK1	0.1439	0
ROS1	MAPK14	0.4289	0
ROS1	MAPK3	0.1126	0
ROS1	MAPK8	-0.4625	0
ROS1	MAPK9	0.1813	0
ROS1	MDM4	0.0027	0
ROS1	MSH2	-0.0524	0
ROS1	MTOR	-0.7019	1
ROS1	NF1	0.6893	1
ROS1	NF2	0.2383	0
ROS1	NFIC	-0.1175	0
ROS1	NFKB2	0.0397	0
ROS1	NOTCH1	-0.7708	1
ROS1	PIK3R1	-0.2616	0
ROS1	PRKAA2	0.2507	0
ROS1	PTEN	0.8949	1
ROS1	RASSF1	-0.1927	0
ROS1	RB1	0.1438	0
ROS1	RELA	-0.0283	0
ROS1	SFN	-0.3281	0
ROS1	SMAD1	0.1831	0
ROS1	SMAD2	0.1689	0
ROS1	SMAD4	0.7347	1
ROS1	SMARCA4	0.1741	0
ROS1	SOX3	-0.3099	0
ROS1	SOX4	-0.7564	1
ROS1	STAT1	-0.3173	0

GENE1	GENE2	R	Positive
ROS1	STAT2	-0.3818	0
ROS1	STK11	0.7794	1
ROS1	STK3	-0.5263	1
ROS1	STK4	-0.4922	0
ROS1	TEAD2	-0.5826	1
ROS1	TERT	-0.4657	0
ROS1	TNFRSF1A	-0.2525	0
ROS1	TP53	-0.4287	0
ROS1	TSC1	0.1881	0
ROS1	WT1	0.4879	0
ROS1	YWHAZ	-0.4539	0
SFN	ABCB5	-0.2688	0
SFN	AKT1S1	0.2450	0
SFN	APC	-0.4153	0
SFN	BAX	0.3334	0
SFN	BCL2	-0.7421	1
SFN	CDC42	0.6000	1
SFN	DVL1	0.0693	0
SFN	E2F1	-0.8127	1
SFN	GSK3B	-0.2819	0
SFN	HIF1A	-0.4291	0
SFN	IGF1R	0.0285	0
SFN	JAK1	0.2098	0
SFN	JAK2	0.8378	1
SFN	KEAP1	-0.5589	1
SFN	MAP2K1	0.6276	1
SFN	MAP2K4	0.2290	0
SFN	MAP3K5	-0.0936	0
SFN	MAPK1	0.0768	0
SFN	MAPK3	-0.7750	1
SFN	MAPK8	0.7556	1
SFN	MAPK9	-0.8100	1
SFN	MDM4	0.4564	0
SFN	MTOR	0.4245	0
SFN	NFIC	0.2706	0
SFN	NFKB2	0.2151	0
SFN	NOTCH1	0.0990	0
SFN	PRKAA2	0.0330	0
SFN	RELA	0.0023	0
SFN	SMAD1	-0.2745	0
SFN	SMAD4	-0.7686	1
SFN	STAT1	-0.2704	0
SFN	STAT2	-0.2898	0
SFN	STK3	0.6373	1
SFN	STK4	0.9174	1
SFN	TNFRSF1A	-0.3154	0
SMAD1	APC	-0.4890	0
SMAD1	BAX	0.2248	0
SMAD1	BCL2	0.2026	0

GENE1	GENE2	R	Positive
SMAD1	CDC42	-0.6836	1
SMAD1	DVL1	0.3185	0
SMAD1	GSK3B	0.4944	0
SMAD1	JAK1	0.2433	0
SMAD1	JAK2	-0.1479	0
SMAD1	MAP2K1	0.2353	0
SMAD1	MAP2K4	0.4320	0
SMAD1	MAPK1	-0.5504	1
SMAD1	MAPK3	0.5966	1
SMAD1	MAPK8	-0.5281	1
SMAD1	MAPK9	0.3188	0
SMAD1	MTOR	0.0517	0
SMAD1	NFKB2	-0.6287	1
SMAD1	NOTCH1	-0.2285	0
SMAD1	PRKAA2	0.9363	1
SMAD1	SMAD4	0.1830	0
SMAD1	STAT1	0.3231	0
SMAD1	STAT2	-0.5874	1
SMAD2	ABCB5	0.7311	1
SMAD2	AKT1S1	-0.6600	1
SMAD2	APC	0.4200	0
SMAD2	ARNT	-0.2461	0
SMAD2	BAX	0.4223	0
SMAD2	BCL2	0.7651	1
SMAD2	BECN1	0.8568	1
SMAD2	CCND2	-0.3649	0
SMAD2	CCNE1	0.4020	0
SMAD2	CD44	0.0907	0
SMAD2	CDC42	-0.9367	1
SMAD2	CDK4	-0.8772	1
SMAD2	CDK6	-0.5141	1
SMAD2	CDKN2C	-0.2400	0
SMAD2	DACH1	-0.7192	1
SMAD2	DVL1	0.4525	0
SMAD2	E2F1	0.1690	0
SMAD2	EPHA2	0.0121	0
SMAD2	FGFR4	0.3849	0
SMAD2	FZR1	-0.8647	1
SMAD2	GLIS2	0.7762	1
SMAD2	GRM1	-0.2588	0
SMAD2	GSK3B	0.8280	1
SMAD2	HGF	-0.0108	0
SMAD2	HIF1A	0.8319	1
SMAD2	IGF1R	-0.7098	1
SMAD2	JAK1	0.3180	0
SMAD2	JAK2	-0.7138	1
SMAD2	KAT2A	-0.3212	0
SMAD2	KDELR2	0.3398	0
SMAD2	KEAP1	0.2123	0
SMAD2	MAP2K1	-0.3309	0
SMAD2	MAP2K3	-0.2588	0
SMAD2	MAP2K4	0.5451	1
SMAD2	MAP2K5	-0.3091	0
SMAD2	MAP2K6	0.7906	1
SMAD2	MAP3K5	0.2087	0
SMAD2	MAPK1	-0.1335	0
SMAD2	MAPK14	0.8973	1
SMAD2	MAPK3	0.9022	1
SMAD2	MAPK8	-0.8973	1
SMAD2	MAPK9	0.2571	0
SMAD2	MDM4	0.0731	0
SMAD2	MSH2	0.7053	1
SMAD2	MTOR	0.0696	0
SMAD2	NF1	0.1903	0
SMAD2	NFIC	0.4605	0
SMAD2	NFKB2	-0.6655	1
SMAD2	NOTCH1	-0.3781	0
SMAD2	PRKAA2	0.3642	0
SMAD2	RELA	-0.0686	0
SMAD2	SFN	-0.6776	1
SMAD2	SMAD1	0.5613	1
SMAD2	SMAD4	0.3019	0
SMAD2	SOX3	-0.4996	0
SMAD2	SOX4	0.2908	0
SMAD2	STAT1	0.5620	1
SMAD2	STAT2	0.2038	0
SMAD2	STK3	-0.0589	0
SMAD2	STK4	-0.6276	1
SMAD2	TEAD2	-0.4140	0
SMAD2	TERT	0.1853	0
SMAD2	TNFRSF1A	-0.0289	0
SMAD2	YWHAZ	-0.6208	1
SMARCA4	ABCB5	0.7237	1
SMARCA4	AKT1S1	-0.6447	1
SMARCA4	APC	-0.1155	0
SMARCA4	ARNT	0.0214	0
SMARCA4	BAX	0.6357	1
SMARCA4	BCL2	0.3247	0
SMARCA4	BECN1	0.6297	1
SMARCA4	CCND2	-0.0721	0
SMARCA4	CCNE1	-0.0281	0
SMARCA4	CD44	0.0053	0
SMARCA4	CDC42	-0.7250	1
SMARCA4	CDK4	-0.4647	0
SMARCA4	CDK6	-0.6889	1
SMARCA4	CDKN2C	-0.2145	0
SMARCA4	DACH1	-0.8181	1
SMARCA4	DVL1	0.5032	1

GENE1	GENE2	R	Positive
SMARCA4	E2F1	-0.3486	0
SMARCA4	EPHA2	-0.1144	0
SMARCA4	FGFR4	-0.1737	0
SMARCA4	FZR1	-0.8679	1
SMARCA4	GLIS2	0.5051	1
SMARCA4	GRM1	0.3447	0
SMARCA4	GSK3B	0.7840	1
SMARCA4	HGF	0.6514	1
SMARCA4	HIF1A	0.8051	1
SMARCA4	IGF1R	-0.8459	1
SMARCA4	JAK1	0.4874	0
SMARCA4	JAK2	-0.2649	0
SMARCA4	KAT2A	-0.2723	0
SMARCA4	KDELR2	0.2515	0
SMARCA4	KEAP1	0.1878	0
SMARCA4	MAP2K1	0.0858	0
SMARCA4	MAP2K3	-0.6423	1
SMARCA4	MAP2K4	0.8735	1
SMARCA4	MAP2K5	0.1707	0
SMARCA4	MAP2K6	0.9182	1
SMARCA4	MAP3K5	0.4528	0
SMARCA4	MAPK1	-0.1747	0
SMARCA4	MAPK14	0.7701	1
SMARCA4	MAPK3	0.6222	1
SMARCA4	MAPK8	-0.5754	1
SMARCA4	MAPK9	-0.1403	0
SMARCA4	MDM4	0.4522	0
SMARCA4	MSH2	0.8524	1
SMARCA4	MTOR	0.1933	0
SMARCA4	NF1	0.4199	0
SMARCA4	NFIC	0.6505	1
SMARCA4	NFKB2	-0.6486	1
SMARCA4	NOTCH1	-0.4628	0
SMARCA4	PIK3R1	0.7618	1
SMARCA4	PRKAA2	0.8064	1
SMARCA4	PTEN	0.1683	0
SMARCA4	RB1	-0.1271	0
SMARCA4	RELA	-0.4076	0
SMARCA4	SFN	-0.1082	0
SMARCA4	SMAD1	0.7924	1
SMARCA4	SMAD2	0.7326	1
SMARCA4	SMAD4	-0.0144	0
SMARCA4	SOX3	-0.7717	1
SMARCA4	SOX4	-0.1053	0
SMARCA4	STAT1	0.5250	1
SMARCA4	STAT2	-0.3708	0
SMARCA4	STK3	0.4993	0
SMARCA4	STK4	-0.1848	0
SMARCA4	TEAD2	-0.8007	1

GENE1	GENE2	R	Positive
SMARCA4	TERT	-0.0079	0
SMARCA4	TNFRSF1A	-0.4505	0
SMARCA4	WT1	-0.2190	0
SMARCA4	YWHAZ	-0.4318	0
SOX3	ABCB5	-0.5113	1
SOX3	AKT1S1	0.6866	1
SOX3	APC	0.4929	0
SOX3	BAX	-0.1800	0
SOX3	BCL2	-0.0571	0
SOX3	CDC42	0.6411	1
SOX3	DVL1	-0.1787	0
SOX3	E2F1	0.0636	0
SOX3	GSK3B	-0.3756	0
SOX3	HIF1A	-0.6261	1
SOX3	IGF1R	0.5788	1
SOX3	JAK1	-0.3517	0
SOX3	JAK2	0.1519	0
SOX3	KEAP1	-0.6607	1
SOX3	MAP2K1	-0.1935	0
SOX3	MAP2K4	-0.4316	0
SOX3	MAP3K5	-0.8505	1
SOX3	MAPK1	0.5866	1
SOX3	MAPK14	-0.7138	1
SOX3	MAPK3	-0.4881	0
SOX3	MAPK8	0.5383	1
SOX3	MAPK9	-0.2528	0
SOX3	MDM4	-0.4660	0
SOX3	MTOR	-0.0261	0
SOX3	NFIC	-0.0549	0
SOX3	NFKB2	0.6428	1
SOX3	NOTCH1	0.3683	0
SOX3	PRKAA2	-0.9616	1
SOX3	RELA	0.8509	1
SOX3	SFN	0.2199	0
SOX3	SMAD1	-0.9815	1
SOX3	SMAD4	-0.1966	0
SOX3	STAT1	-0.1604	0
SOX3	STAT2	0.6417	1
SOX3	STK3	-0.4024	0
SOX3	STK4	0.4594	0
SOX3	TERT	-0.2201	0
SOX3	TNFRSF1A	0.0987	0
SOX4	ABCB5	0.5681	1
SOX4	AKT1S1	-0.0189	0
SOX4	APC	0.3280	0
SOX4	BAX	0.2827	0
SOX4	BCL2	0.6999	1
SOX4	CDC42	-0.2319	0
SOX4	DVL1	0.6986	1

GENE1	GENE2	R	Positive
SOX4	E2F1	0.3215	0
SOX4	GSK3B	0.5193	1
SOX4	HIF1A	0.4338	0
SOX4	IGF1R	-0.2195	0
SOX4	JAK1	-0.3945	0
SOX4	JAK2	0.0060	0
SOX4	KEAP1	-0.4108	0
SOX4	MAP2K1	0.1666	0
SOX4	MAP2K4	-0.0923	0
SOX4	MAP3K5	-0.6235	1
SOX4	MAPK1	0.1021	0
SOX4	MAPK14	0.0141	0
SOX4	MAPK3	0.4108	0
SOX4	MAPK8	0.0127	0
SOX4	MAPK9	0.2320	0
SOX4	MDM4	-0.4356	0
SOX4	MTOR	0.3363	0
SOX4	NFIC	0.1070	0
SOX4	NFKB2	-0.0240	0
SOX4	NOTCH1	0.6805	1
SOX4	PRKAA2	-0.4171	0
SOX4	RELA	0.2803	0
SOX4	SFN	-0.2961	0
SOX4	SMAD1	-0.1696	0
SOX4	SMAD4	-0.2110	0
SOX4	SOX3	0.3425	0
SOX4	STAT1	0.6198	1
SOX4	STAT2	0.6568	1
SOX4	STK3	0.0353	0
SOX4	STK4	0.0118	0
SOX4	TERT	0.3183	0
SOX4	TNFRSF1A	0.2537	0
STK11	ABCB5	0.1671	0
STK11	AKT1S1	-0.1815	0
STK11	APC	0.0718	0
STK11	ARNT	0.3289	0
STK11	BAX	-0.0019	0
STK11	BCL2	0.3111	0
STK11	BECN1	0.6043	1
STK11	CCND2	-0.0978	0
STK11	CCNE1	0.1781	0
STK11	CD44	0.5543	1
STK11	CDC42	-0.4341	0
STK11	CDK4	-0.5714	1
STK11	CDK6	0.0124	0
STK11	CDKN2A	-0.6221	1
STK11	CDKN2B	0.9319	1
STK11	CDKN2C	0.0769	0
STK11	DACH1	-0.3639	0

GENE1	GENE2	R	Positive
STK11	DVL1	-0.1878	0
STK11	E2F1	0.0683	0
STK11	EPHA2	-0.0305	0
STK11	FGFR4	-0.2035	0
STK11	FZR1	-0.7197	1
STK11	GLIS2	0.4419	0
STK11	GRM1	0.2784	0
STK11	GSK3B	0.3557	0
STK11	HGF	0.4770	0
STK11	HIF1A	0.4145	0
STK11	IGF1R	-0.2439	0
STK11	JAK1	0.2059	0
STK11	JAK2	-0.7029	1
STK11	KAT2A	-0.0141	0
STK11	KDELR2	0.1283	0
STK11	KEAP1	0.5374	1
STK11	LATS2	-0.1433	0
STK11	MAP2K1	-0.5769	1
STK11	MAP2K3	-0.0708	0
STK11	MAP2K4	0.4907	0
STK11	MAP2K5	0.5315	1
STK11	MAP2K6	0.5125	1
STK11	MAP3K5	0.3677	0
STK11	MAPK1	0.2268	0
STK11	MAPK14	0.8059	1
STK11	MAPK3	0.5734	1
STK11	MAPK8	-0.6309	1
STK11	MAPK9	0.1432	0
STK11	MDM4	0.0098	0
STK11	MSH2	0.5005	1
STK11	MTOR	-0.5301	1
STK11	NF1	0.6165	1
STK11	NFIC	0.3068	0
STK11	NFKB2	-0.1791	0
STK11	NOTCH1	-0.6859	1
STK11	PIK3R1	0.1308	0
STK11	PRKAA2	0.5383	1
STK11	PTEN	0.6337	1
STK11	RASSF1	-0.5770	1
STK11	RB1	0.1671	0
STK11	RELA	-0.1311	0
STK11	SFN	-0.3930	0
STK11	SMAD1	0.5242	1
STK11	SMAD2	0.5928	1
STK11	SMAD4	0.6242	1
STK11	SMARCA4	0.6830	1
STK11	SOX3	-0.5631	1
STK11	SOX4	-0.4414	0
STK11	STAT1	0.2933	0

GENE1	GENE2	R	Positive
STK11	STAT2	-0.4359	0
STK11	STK3	-0.1661	0
STK11	STK4	-0.4461	0
STK11	TEAD2	-0.7469	1
STK11	TERT	-0.5076	1
STK11	TNFRSF1A	-0.5746	1
STK11	TP53	0.1443	0
STK11	TSC1	0.6835	1
STK11	WT1	0.4215	0
STK11	YWHAZ	-0.4480	0
STK3	APC	-0.6047	1
STK3	BAX	0.6847	1
STK3	BCL2	-0.2448	0
STK3	CDC42	-0.0882	0
STK3	DVL1	0.6627	1
STK3	E2F1	-0.6864	1
STK3	GSK3B	0.3812	0
STK3	JAK1	0.2724	0
STK3	JAK2	0.6967	1
STK3	KEAP1	-0.2776	0
STK3	MAP2K1	0.8752	1
STK3	MAP2K4	0.5700	1
STK3	MAPK1	-0.3189	0
STK3	MAPK3	-0.0979	0
STK3	MAPK8	0.3027	0
STK3	MAPK9	-0.4794	0
STK3	MTOR	0.6513	1
STK3	NFIC	0.4247	0
STK3	NFKB2	-0.3375	0
STK3	NOTCH1	0.1905	0
STK3	PRKAA2	0.5826	1
STK3	SMAD1	0.4400	0
STK3	SMAD4	-0.7353	1
STK3	STAT1	0.2724	0
STK3	STAT2	-0.4185	0
STK3	TNFRSF1A	-0.2787	0
STK4	APC	-0.2485	0
STK4	BAX	0.3559	0
STK4	BCL2	-0.4659	0
STK4	CDC42	0.6571	1
STK4	DVL1	0.2128	0
STK4	E2F1	-0.6744	1
STK4	GSK3B	-0.1354	0
STK4	JAK1	-0.0429	0
STK4	JAK2	0.7693	1
STK4	KEAP1	-0.7268	1
STK4	MAP2K1	0.5350	1
STK4	MAP2K4	0.1928	0
STK4	MAPK1	0.3702	0

GENE1	GENE2	R	Positive
STK4	MAPK3	-0.6461	1
STK4	MAPK8	0.8333	1
STK4	MAPK9	-0.7627	1
STK4	MTOR	0.3606	0
STK4	NFIC	0.3463	0
STK4	NFKB2	0.4125	0
STK4	NOTCH1	0.3198	0
STK4	PRKAA2	-0.2062	0
STK4	SMAD1	-0.4541	0
STK4	SMAD4	-0.7430	1
STK4	STAT1	0.0079	0
STK4	STAT2	-0.0885	0
STK4	STK3	0.5524	1
STK4	TNFRSF1A	-0.4244	0
TCF3	ABCB5	0.0821	0
TCF3	AKT1S1	-0.4301	0
TCF3	APC	0.0016	0
TCF3	ARNT	-0.1063	0
TCF3	BAX	0.4733	0
TCF3	BCL2	-0.2508	0
TCF3	BECN1	0.2098	0
TCF3	CBLB	0.4293	0
TCF3	CCND2	-0.2132	0
TCF3	CCNE1	-0.2552	0
TCF3	CD44	-0.1743	0
TCF3	CDC42	-0.3232	0
TCF3	CDK4	-0.0052	0
TCF3	CDK6	-0.1784	0
TCF3	CDKN2A	-0.6404	1
TCF3	CDKN2B	0.4806	0
TCF3	CDKN2C	0.3323	0
TCF3	CTNNB1	-0.1083	0
TCF3	DACH1	-0.3287	0
TCF3	DVL1	-0.0336	0
TCF3	E2F1	-0.6661	1
TCF3	EGFR	-0.3035	0
TCF3	EPHA2	-0.4077	0
TCF3	FGFR4	-0.7398	1
TCF3	FOXO1	0.2768	0
TCF3	FZR1	-0.6862	1
TCF3	GLIS2	-0.0249	0
TCF3	GRM1	0.7846	1
TCF3	GSK3B	0.2549	0
TCF3	HGF	0.7249	1
TCF3	HIF1A	0.1703	0
TCF3	IGF1R	-0.5110	1
TCF3	JAK1	0.7815	1
TCF3	JAK2	-0.1827	0
TCF3	KAT2A	0.0290	0

GENE1	GENE2	R	Positive
TCF3	KDELR2	-0.4415	0
TCF3	KEAP1	0.0410	0
TCF3	LATS2	0.4771	0
TCF3	MAP2K1	-0.0952	0
TCF3	MAP2K3	-0.2578	0
TCF3	MAP2K4	0.8044	1
TCF3	MAP2K5	0.6578	1
TCF3	MAP2K6	0.4391	0
TCF3	MAP3K5	0.5193	1
TCF3	MAPK1	-0.0579	0
TCF3	MAPK14	0.3745	0
TCF3	MAPK3	0.0397	0
TCF3	MAPK8	-0.3841	0
TCF3	MAPK9	-0.5498	1
TCF3	MDM4	0.6917	1
TCF3	MSH2	0.2702	0
TCF3	MTOR	0.1087	0
TCF3	NF1	0.9121	1
TCF3	NF2	-0.0102	0
TCF3	NFIC	0.6452	1
TCF3	NFKB2	-0.4633	0
TCF3	NOTCH1	-0.8591	1
TCF3	PIK3CA	-0.5460	1
TCF3	PIK3R1	0.6444	1
TCF3	PRKAA2	0.6094	1
TCF3	PTEN	0.4153	0
TCF3	RASSF1	-0.5740	1
TCF3	RB1	-0.4941	0
TCF3	RELA	-0.1421	0
TCF3	ROS1	0.5208	1
TCF3	SFN	0.2482	0
TCF3	SMAD1	0.3988	0
TCF3	SMAD2	0.3215	0
TCF3	SMAD4	-0.0836	0
TCF3	SMARCA4	0.7147	1
TCF3	SOX3	-0.5069	1
TCF3	SOX4	-0.6454	1
TCF3	STAT1	-0.0838	0
TCF3	STAT2	-0.4039	0
TCF3	STK11	0.6606	1
TCF3	STK3	0.3021	0
TCF3	STK4	0.0389	0
TCF3	TEAD2	-0.9234	1
TCF3	TERT	-0.2014	0
TCF3	TNFRSF1A	-0.4736	0
TCF3	TP53	0.3751	0
TCF3	TSC1	0.1233	0
TCF3	WT1	-0.1149	0
TCF3	YAP1	0.4389	0

GENE1	GENE2	R	Positive
TCF3	YWHAZ	-0.2746	0
TEAD2	ABCB5	-0.2051	0
TEAD2	AKT1S1	0.5544	1
TEAD2	APC	0.2181	0
TEAD2	BAX	-0.3233	0
TEAD2	BCL2	0.1771	0
TEAD2	CD44	0.0288	0
TEAD2	CDC42	0.4680	0
TEAD2	CDKN2C	-0.3615	0
TEAD2	DACH1	0.3271	0
TEAD2	DVL1	0.0418	0
TEAD2	E2F1	0.4495	0
TEAD2	GRM1	-0.7547	1
TEAD2	GSK3B	-0.2738	0
TEAD2	HGF	-0.8082	1
TEAD2	HIF1A	-0.3381	0
TEAD2	IGF1R	0.5355	1
TEAD2	JAK1	-0.6751	1
TEAD2	JAK2	0.2336	0
TEAD2	KAT2A	-0.2282	0
TEAD2	KEAP1	-0.3791	0
TEAD2	MAP2K1	0.0480	0
TEAD2	MAP2K4	-0.7001	1
TEAD2	MAP2K6	-0.4995	0
TEAD2	MAP3K5	-0.7544	1
TEAD2	MAPK1	0.2722	0
TEAD2	MAPK14	-0.5756	1
TEAD2	MAPK3	-0.2224	0
TEAD2	MAPK8	0.5074	1
TEAD2	MAPK9	0.2372	0
TEAD2	MDM4	-0.6529	1
TEAD2	MTOR	-0.0002	0
TEAD2	NFIC	-0.4178	0
TEAD2	NFKB2	0.5560	1
TEAD2	NOTCH1	0.7959	1
TEAD2	PRKAA2	-0.8359	1
TEAD2	RELA	0.4664	0
TEAD2	SFN	-0.0526	0
TEAD2	SMAD1	-0.6924	1
TEAD2	SMAD4	-0.1040	0
TEAD2	SOX3	0.7873	1
TEAD2	SOX4	0.6665	1
TEAD2	STAT1	0.0372	0
TEAD2	STAT2	0.5962	1
TEAD2	STK3	-0.3175	0
TEAD2	STK4	0.2087	0
TEAD2	TERT	0.0959	0
TEAD2	TNFRSF1A	0.3827	0
TEAD2	YWHAZ	0.5327	1

GENE1	GENE2	R	Positive
TERT	ABCB5	0.3539	0
TERT	AKT1S1	-0.7205	1
TERT	APC	0.0218	0
TERT	BAX	0.2798	0
TERT	BCL2	-0.0334	0
TERT	CDC42	-0.4786	0
TERT	DVL1	0.3876	0
TERT	E2F1	-0.0314	0
TERT	GSK3B	0.1173	0
TERT	HIF1A	0.1977	0
TERT	IGF1R	-0.4314	0
TERT	JAK1	0.3821	0
TERT	JAK2	0.2142	0
TERT	KEAP1	-0.0698	0
TERT	MAP2K1	0.4751	0
TERT	MAP2K4	-0.0751	0
TERT	MAP3K5	0.3705	0
TERT	MAPK1	-0.8679	1
TERT	MAPK3	0.0296	0
TERT	MAPK8	-0.2565	0
TERT	MAPK9	0.1147	0
TERT	MDM4	0.4733	0
TERT	MTOR	0.7123	1
TERT	NFIC	-0.1332	0
TERT	NFKB2	-0.7060	1
TERT	NOTCH1	0.1336	0
TERT	PRKAA2	0.0993	0
TERT	RELA	-0.3557	0
TERT	SFN	-0.1320	0
TERT	SMAD1	0.2355	0
TERT	SMAD4	-0.3961	0
TERT	STAT1	-0.2361	0
TERT	STAT2	0.3619	0
TERT	STK3	0.2200	0
TERT	STK4	-0.2796	0
TERT	TNFRSF1A	0.8443	1
TNFRSF1A	APC	0.2036	0
TNFRSF1A	BAX	-0.1985	0
TNFRSF1A	BCL2	-0.0717	0
TNFRSF1A	CDC42	-0.2043	0
TNFRSF1A	DVL1	-0.0536	0
TNFRSF1A	E2F1	0.3192	0
TNFRSF1A	GSK3B	-0.2767	0
TNFRSF1A	JAK1	0.1198	0
TNFRSF1A	JAK2	0.0345	0
TNFRSF1A	MAP2K1	0.1168	0
TNFRSF1A	MAP2K4	-0.5284	1
TNFRSF1A	MAPK1	-0.6759	1
TNFRSF1A	MAPK3	-0.1184	0

GENE1	GENE2	R	Positive
TNFRSF1A	MAPK8	-0.1852	0
TNFRSF1A	MAPK9	0.3422	0
TNFRSF1A	MTOR	0.3434	0
TNFRSF1A	NFKB2	-0.3554	0
TNFRSF1A	NOTCH1	0.1690	0
TNFRSF1A	PRKAA2	-0.2840	0
TNFRSF1A	SMAD1	-0.1106	0
TNFRSF1A	SMAD4	-0.0732	0
TNFRSF1A	STAT1	-0.4887	0
TNFRSF1A	STAT2	0.5152	1
TP53	ABCB5	0.7070	1
TP53	AKT1S1	-0.2769	0
TP53	APC	0.1290	0
TP53	ARNT	-0.4017	0
TP53	BAX	0.9069	1
TP53	BCL2	0.3694	0
TP53	BECN1	0.0335	0
TP53	CCND2	-0.4817	0
TP53	CCNE1	0.3482	0
TP53	CD44	-0.2447	0
TP53	CDC42	-0.3549	0
TP53	CDK4	-0.0379	0
TP53	CDK6	-0.8363	1
TP53	CDKN2C	-0.6733	1
TP53	DACH1	-0.9048	1
TP53	DVL1	0.8269	1
TP53	E2F1	-0.5576	1
TP53	EPHA2	0.4095	0
TP53	FGFR4	-0.1461	0
TP53	FZR1	-0.6701	1
TP53	GLIS2	-0.0264	0
TP53	GRM1	0.0301	0
TP53	GSK3B	0.8331	1
TP53	HGF	0.2851	0
TP53	HIF1A	0.6168	1
TP53	IGF1R	-0.7549	1
TP53	JAK1	0.2844	0
TP53	JAK2	0.1022	0
TP53	KAT2A	-0.8381	1
TP53	KDELR2	0.2023	0
TP53	KEAP1	-0.5589	1
TP53	MAP2K1	0.3058	0
TP53	MAP2K3	-0.9040	1
TP53	MAP2K4	0.8420	1
TP53	MAP2K5	-0.0630	0
TP53	MAP2K6	0.8123	1
TP53	MAP3K5	-0.2665	0
TP53	MAPK1	0.2140	0
TP53	MAPK14	0.2354	0

GENE1	GENE2	R	Positive
TP53	MAPK3	0.3340	0
TP53	MAPK8	-0.0695	0
TP53	MAPK9	-0.5320	1
TP53	MDM4	0.2539	0
TP53	MSH2	0.5115	1
TP53	MTOR	0.5242	1
TP53	NF1	0.1270	0
TP53	NFIC	0.8988	1
TP53	NFKB2	-0.3212	0
TP53	NOTCH1	-0.0027	0
TP53	PIK3R1	0.7577	1
TP53	PRKAA2	0.2332	0
TP53	PTEN	-0.5933	1
TP53	RB1	-0.2905	0
TP53	RELA	0.1345	0
TP53	SFN	0.2630	0
TP53	SMAD1	0.1914	0
TP53	SMAD2	0.4437	0
TP53	SMAD4	-0.5228	1
TP53	SMARCA4	0.6565	1
TP53	SOX3	-0.1019	0
TP53	SOX4	0.3923	0
TP53	STAT1	0.7002	1
TP53	STAT2	0.1014	0
TP53	STK3	0.6262	1
TP53	STK4	0.4143	0
TP53	TEAD2	-0.2279	0
TP53	TERT	-0.0576	0
TP53	TNFRSF1A	-0.4997	0
TP53	WT1	-0.1581	0
TP53	YWHAZ	0.2724	0
TSC1	ABCB5	0.6366	1
TSC1	AKT1S1	-0.1219	0
TSC1	APC	-0.1732	0
TSC1	ARNT	0.4844	0
TSC1	BAX	0.0708	0
TSC1	BCL2	0.7042	1
TSC1	BECN1	0.6643	1
TSC1	CCND2	0.1338	0
TSC1	CCNE1	0.3572	0
TSC1	CD44	0.6726	1
TSC1	CDC42	-0.4854	0
TSC1	CDK4	-0.7021	1
TSC1	CDK6	-0.4136	0
TSC1	CDKN2C	-0.5496	1
TSC1	DACH1	-0.6681	1
TSC1	DVL1	0.3288	0
TSC1	E2F1	0.3502	0
TSC1	EPHA2	0.3954	0

GENE1	GENE2	R	Positive
TSC1	FGFR4	0.3369	0
TSC1	FZR1	-0.5546	1
TSC1	GLIS2	0.6772	1
TSC1	GRM1	-0.2113	0
TSC1	GSK3B	0.6629	1
TSC1	HGF	0.3069	0
TSC1	HIF1A	0.8029	1
TSC1	IGF1R	-0.3460	0
TSC1	JAK1	-0.2984	0
TSC1	JAK2	-0.4598	0
TSC1	KAT2A	-0.3384	0
TSC1	KDELR2	0.8005	1
TSC1	KEAP1	0.4916	0
TSC1	MAP2K1	-0.1958	0
TSC1	MAP2K3	-0.4559	0
TSC1	MAP2K4	0.3410	0
TSC1	MAP2K5	0.0123	0
TSC1	MAP2K6	0.7414	1
TSC1	MAP3K5	0.0792	0
TSC1	MAPK1	0.1958	0
TSC1	MAPK14	0.8236	1
TSC1	MAPK3	0.8581	1
TSC1	MAPK8	-0.4246	0
TSC1	MAPK9	0.4655	0
TSC1	MDM4	-0.3367	0
TSC1	MSH2	0.8435	1
TSC1	MTOR	-0.3879	0
TSC1	NF1	-0.1015	0
TSC1	NFIC	0.1681	0
TSC1	NFKB2	-0.0899	0
TSC1	NOTCH1	0.0162	0
TSC1	PIK3R1	0.0932	0
TSC1	PRKAA2	0.5136	1
TSC1	PTEN	0.1688	0
TSC1	RB1	0.3815	0
TSC1	RELA	-0.3170	0
TSC1	SFN	-0.4954	0
TSC1	SMAD1	0.6560	1
TSC1	SMAD2	0.6402	1
TSC1	SMAD4	0.4958	0
TSC1	SMARCA4	0.6605	1
TSC1	SOX3	-0.5543	1
TSC1	SOX4	0.1843	0
TSC1	STAT1	0.8144	1
TSC1	STAT2	-0.3705	0
TSC1	STK3	0.1011	0
TSC1	STK4	-0.3711	0
TSC1	TEAD2	-0.3246	0
TSC1	TERT	-0.3373	0

GENE1	GENE2	R	Positive
TSC1	TNFRSF1A	-0.5169	1
TSC1	TP53	0.3547	0
TSC1	WT1	0.2466	0
TSC1	YWHAZ	-0.2761	0
WHSC1L1	ABCB5	0.6636	1
WHSC1L1	AKT1S1	-0.1928	0
WHSC1L1	APC	0.1462	0
WHSC1L1	ARNT	0.1615	0
WHSC1L1	BAX	-0.0467	0
WHSC1L1	BCL2	0.9177	1
WHSC1L1	BECN1	0.6969	1
WHSC1L1	CBLB	0.4223	0
WHSC1L1	CCND2	0.0218	0
WHSC1L1	CCNE1	0.5248	1
WHSC1L1	CD44	0.4651	0
WHSC1L1	CDC42	-0.5790	1
WHSC1L1	CDK4	-0.8457	1
WHSC1L1	CDK6	-0.3656	0
WHSC1L1	CDKN2A	0.2438	0
WHSC1L1	CDKN2B	0.2444	0
WHSC1L1	CDKN2C	-0.5708	1
WHSC1L1	CTNNB1	-0.0493	0
WHSC1L1	DACH1	-0.4919	0
WHSC1L1	DVL1	0.4214	0
WHSC1L1	E2F1	0.7059	1
WHSC1L1	EGFR	0.4299	0
WHSC1L1	EPHA2	0.4060	0
WHSC1L1	FGFR4	0.8694	1
WHSC1L1	FOXO1	-0.6938	1
WHSC1L1	FZR1	-0.3025	0
WHSC1L1	GLIS2	0.8094	1
WHSC1L1	GRM1	-0.7451	1
WHSC1L1	GSK3B	0.6059	1
WHSC1L1	HGF	-0.3297	0
WHSC1L1	HIF1A	0.7442	1
WHSC1L1	IGF1R	-0.2441	0
WHSC1L1	JAK1	-0.4446	0
WHSC1L1	JAK2	-0.5118	1
WHSC1L1	KAT2A	-0.3132	0
WHSC1L1	KDELR2	0.8486	1
WHSC1L1	KEAP1	0.3605	0
WHSC1L1	LATS2	-0.7106	1
WHSC1L1	MAP2K1	-0.2143	0
WHSC1L1	MAP2K3	-0.1668	0
WHSC1L1	MAP2K4	-0.0643	0
WHSC1L1	MAP2K5	-0.6117	1
WHSC1L1	MAP2K6	0.5053	1
WHSC1L1	MAP3K5	-0.1321	0
WHSC1L1	MAPK1	0.0010	0

GENE1	GENE2	R	Positive
WHSC1L1	MAPK14	0.6708	1
WHSC1L1	MAPK3	0.8965	1
WHSC1L1	MAPK8	-0.4905	0
WHSC1L1	MAPK9	0.7473	1
WHSC1L1	MDM4	-0.5402	1
WHSC1L1	MSH2	0.6314	1
WHSC1L1	MTOR	-0.2065	0
WHSC1L1	NF1	-0.5292	1
WHSC1L1	NF2	0.5046	1
WHSC1L1	NFIC	-0.1049	0
WHSC1L1	NFKB2	-0.1568	0
WHSC1L1	NOTCH1	0.3332	0
WHSC1L1	PIK3CA	0.7579	1
WHSC1L1	PIK3R1	-0.1194	0
WHSC1L1	PRKAA2	0.0772	0
WHSC1L1	PTEN	-0.1097	0
WHSC1L1	RASSF1	0.0508	0
WHSC1L1	RB1	0.8117	1
WHSC1L1	RELA	-0.1328	0
WHSC1L1	ROS1	-0.1385	0
WHSC1L1	SFN	-0.8017	1
WHSC1L1	SMAD1	0.3988	0
WHSC1L1	SMAD2	0.6728	1
WHSC1L1	SMAD4	0.4713	0
WHSC1L1	SMARCA4	0.2677	0
WHSC1L1	SOX3	-0.2501	0
WHSC1L1	SOX4	0.6540	1
WHSC1L1	STAT1	0.7275	1
WHSC1L1	STAT2	0.1966	0
WHSC1L1	STK11	0.2309	0
WHSC1L1	STK3	-0.1880	0
WHSC1L1	STK4	-0.5971	1
WHSC1L1	TCF3	-0.4046	0
WHSC1L1	TEAD2	0.1927	0
WHSC1L1	TERT	0.1185	0
WHSC1L1	TNFRSF1A	0.0895	0
WHSC1L1	TP53	0.1444	0
WHSC1L1	TSC1	0.7462	1
WHSC1L1	WT1	0.2496	0
WHSC1L1	YAP1	0.5589	1
WHSC1L1	YWHAZ	-0.3560	0
WT1	ABCB5	-0.3319	0
WT1	AKT1S1	0.5647	1
WT1	APC	0.5880	1
WT1	ARNT	0.0590	0
WT1	BAX	-0.4291	0
WT1	BCL2	0.4475	0
WT1	BECN1	0.0099	0
WT1	CCND2	-0.4890	0

GENE1	GENE2	R	Positive
WT1	CCNE1	0.7754	1
WT1	CD44	0.6840	1
WT1	CDC42	0.1637	0
WT1	CDK4	-0.3173	0
WT1	CDK6	0.5273	1
WT1	CDKN2C	-0.1289	0
WT1	DACH1	0.1053	0
WT1	DVL1	-0.4331	0
WT1	E2F1	0.4953	0
WT1	EPHA2	0.5511	1
WT1	FGFR4	0.1637	0
WT1	FZR1	-0.1542	0
WT1	GLIS2	-0.0375	0
WT1	GRM1	-0.4294	0
WT1	GSK3B	-0.0560	0
WT1	HGF	-0.4251	0
WT1	HIF1A	-0.1634	0
WT1	IGF1R	0.4814	0
WT1	JAK1	-0.3968	0
WT1	JAK2	-0.7037	1
WT1	KAT2A	-0.2706	0
WT1	KDELR2	0.0514	0
WT1	KEAP1	0.0815	0
WT1	MAP2K1	-0.9005	1
WT1	MAP2K3	0.3686	0
WT1	MAP2K4	-0.2016	0
WT1	MAP2K5	0.2087	0
WT1	MAP2K6	-0.1359	0
WT1	MAP3K5	-0.5211	1
WT1	MAPK1	0.7969	1
WT1	MAPK14	0.1892	0
WT1	MAPK3	0.2509	0
WT1	MAPK8	-0.1737	0
WT1	MAPK9	0.2433	0
WT1	MDM4	-0.7251	1
WT1	MSH2	-0.2693	0
WT1	MTOR	-0.7446	1
WT1	NF1	0.1573	0
WT1	NFIC	-0.0128	0
WT1	NFKB2	0.5439	1
WT1	NOTCH1	-0.1602	0
WT1	PRKAA2	-0.5146	1
WT1	PTEN	0.1360	0
WT1	RB1	0.5343	1
WT1	RELA	0.6868	1
WT1	SFN	-0.4637	0
WT1	SMAD1	-0.4380	0
WT1	SMAD2	0.1512	0
WT1	SMAD4	0.6787	1
WT1	SOX3	0.4475	0
WT1	SOX4	0.0459	0
WT1	STAT1	0.1918	0
WT1	STAT2	0.2746	0
WT1	STK3	-0.7875	1
WT1	STK4	-0.2311	0
WT1	TEAD2	0.2206	0
WT1	TERT	-0.6880	1
WT1	TNFRSF1A	-0.3221	0
WT1	YWHAZ	0.1766	0
YAP1	ABCB5	0.7470	1
YAP1	AKT1S1	-0.8185	1
YAP1	APC	0.2431	0
YAP1	ARNT	-0.1957	0
YAP1	BAX	0.4685	0
YAP1	BCL2	0.5898	1
YAP1	BECN1	0.8966	1
YAP1	CBLB	0.9103	1
YAP1	CCND2	-0.1966	0
YAP1	CCNE1	0.1371	0
YAP1	CD44	-0.0412	0
YAP1	CDC42	-0.9826	1
YAP1	CDK4	-0.8131	1
YAP1	CDK6	-0.5674	1
YAP1	CDKN2A	-0.3579	0
YAP1	CDKN2B	0.4634	0
YAP1	CDKN2C	-0.0916	0
YAP1	CTNNB1	-0.2818	0
YAP1	DACH1	-0.6878	1
YAP1	DVL1	0.4513	0
YAP1	E2F1	0.0426	0
YAP1	EGFR	0.0111	0
YAP1	EPHA2	-0.2194	0
YAP1	FGFR4	0.2710	0
YAP1	FZR1	-0.8433	1
YAP1	GLIS2	0.8051	1
YAP1	GRM1	-0.0531	0
YAP1	GSK3B	0.7822	1
YAP1	HGF	0.1706	0
YAP1	HIF1A	0.8366	1
YAP1	IGF1R	-0.8033	1
YAP1	JAK1	0.4686	0
YAP1	JAK2	-0.5890	1
YAP1	KAT2A	-0.1468	0
YAP1	KDELR2	0.2693	0
YAP1	KEAP1	0.2928	0
YAP1	LATS2	-0.3324	0
YAP1	MAP2K1	-0.1663	0
YAP1	MAP2K3	-0.2801	0

GENE1	GENE2	R	Positive
YAP1	MAP2K4	0.6001	1
YAP1	MAP2K5	-0.2561	0
YAP1	MAP2K6	0.7970	1
YAP1	MAP3K5	0.4470	0
YAP1	MAPK1	-0.3578	0
YAP1	MAPK14	0.8946	1
YAP1	MAPK3	0.8258	1
YAP1	MAPK8	-0.9045	1
YAP1	MAPK9	0.2099	0
YAP1	MDM4	0.2986	0
YAP1	MSH2	0.7680	1
YAP1	MTOR	0.1799	0
YAP1	NF1	0.2535	0
YAP1	NF2	0.5812	1
YAP1	NFIC	0.4322	0
YAP1	NFKB2	-0.8159	1
YAP1	NOTCH1	-0.4513	0
YAP1	PIK3CA	0.2940	0
YAP1	PIK3R1	0.5751	1
YAP1	PRKAA2	0.5628	1
YAP1	PTEN	0.1926	0
YAP1	RASSF1	-0.1838	0
YAP1	RB1	0.3727	0
YAP1	RELA	-0.2903	0
YAP1	ROS1	0.1845	0
YAP1	SFN	-0.5765	1
YAP1	SMAD1	0.7209	1
YAP1	SMAD2	0.9615	1
YAP1	SMAD4	0.2108	0
YAP1	SMARCA4	0.8162	1
YAP1	SOX3	-0.6851	1
YAP1	SOX4	0.1406	0
YAP1	STAT1	0.4361	0
YAP1	STAT2	0.0463	0
YAP1	STK11	0.5826	1
YAP1	STK3	0.0988	0
YAP1	STK4	-0.6289	1
YAP1	TEAD2	-0.5748	1
YAP1	TERT	0.3182	0
YAP1	TNFRSF1A	0.0304	0
YAP1	TP53	0.4087	0
YAP1	TSC1	0.5837	1
YAP1	WT1	-0.0826	0
YAP1	YWHAZ	-0.7434	1

GENE1	GENE2	R	Positive
YWHAZ	ABCB5	-0.2872	0
YWHAZ	AKT1S1	0.7582	1
YWHAZ	APC	-0.0250	0
YWHAZ	BAX	0.0801	0
YWHAZ	BCL2	-0.1937	0
YWHAZ	CDC42	0.7713	1
YWHAZ	DVL1	0.0855	0
YWHAZ	E2F1	-0.2933	0
YWHAZ	GSK3B	-0.1890	0
YWHAZ	HGF	-0.1130	0
YWHAZ	HIF1A	-0.4177	0
YWHAZ	IGF1R	0.3784	0
YWHAZ	JAK1	-0.4065	0
YWHAZ	JAK2	0.5341	1
YWHAZ	KEAP1	-0.6939	1
YWHAZ	MAP2K1	0.2196	0
YWHAZ	MAP2K4	-0.0847	0
YWHAZ	MAP2K6	-0.2533	0
YWHAZ	MAP3K5	-0.7959	1
YWHAZ	MAPK1	0.6792	1
YWHAZ	MAPK14	-0.7077	1
YWHAZ	MAPK3	-0.5213	1
YWHAZ	MAPK8	0.8444	1
YWHAZ	MAPK9	-0.5019	1
YWHAZ	MDM4	-0.3189	0
YWHAZ	MTOR	0.0525	0
YWHAZ	NFIC	0.1773	0
YWHAZ	NFKB2	0.7217	1
YWHAZ	NOTCH1	0.4947	0
YWHAZ	PRKAA2	-0.5463	1
YWHAZ	RELA	0.5401	1
YWHAZ	SFN	0.6484	1
YWHAZ	SMAD1	-0.6978	1
YWHAZ	SMAD4	-0.4533	0
YWHAZ	SOX3	0.7413	1
YWHAZ	SOX4	0.2250	0
YWHAZ	STAT1	0.1364	0
YWHAZ	STAT2	0.1252	0
YWHAZ	STK3	0.1803	0
YWHAZ	STK4	0.8799	1
YWHAZ	TERT	-0.4762	0
YWHAZ	TNFRSF1A	-0.4206	0

GENE1 – Standard HUGO gene symbol of protein 1; GENE2 - Standard HUGO gene symbol of protein 2; R - Correlation coefficient; Positive – Indicates whether the PPI is positive (1) or negative (0) in the FUSION analysis

Supplementary Table 5. OncoPPi Drug-Oncogene-tumor suppressor connectivity, related to Figure 5 and Supplementary Figure 5

The table includes a list of known drugs that target oncogenic nodes in the OncoPPi network and their current clinical development status. The table also includes a list of tumor suppressors included in OncoPPI and linkage to druggable targets (green boxes).

Oncogene	Drug	Status	Oncogene	Drug	Status
AKT1	AZD5363	Clinical trials	ERBB2	Pertuzumab	FDA approved
AKT1	MK2206	Clinical trials	ERBB2	Trastuzumab	FDA approved
AURKA	Alisertib	Clinical trials	FGFR4	Nintedanib	FDA approved
BRAF	Dabrafenib	FDA approved	FGFR4	Palifermin	FDA approved
BRAF	Sorafenib	FDA approved	HIF1A	Carvedilol	FDA approved
BRAF	Trametinib	FDA approved	HIF1A	CRLX10	Clinical trials
BRAF	Vemurafenib	FDA approved	HRAS	Tipifarnib	FDA approved
CD44	Hyaluronan	FDA approved	IGF1R	Ceritinib	FDA approved
CDK4	Palbociclib	FDA approved	IGF1R	Mecasermin	FDA approved
CDK4	Abemaciclib	Clinical trials	KRAS	Cetuximab	FDA approved
CDK4	FLX925	FDA approved	KRAS	Panitumumab	FDA approved
CDK4	LEE011	FDA approved	MDM2	DS3032b	Clinical trials
CDK6	Palbociclib	FDA approved	MDM2	RO6839921	Clinical trials
CDK6	Abemaciclib	FDA approved	MET	Crizotinib	FDA approved
CDK6	FLX925	Clinical trials	MET	INC280	Clinical trials
CDK6	LEE011	Clinical trials	MET	PH-02341066	Clinical trials
EGFR	Afatinib	FDA approved	PDGFRA	Axitinib	FDA approved
EGFR	Cetuximab	FDA approved	PDGFRA	Becaplermin	FDA approved
EGFR	Erlotinib	FDA approved	PDGFRA	Gleevec	FDA approved
EGFR	Gefitinib	FDA approved	PDGFRA	Imatinib	FDA approved
EGFR	Lapatinib	FDA approved	PDGFRA	Nintedanib	FDA approved
EGFR	Lidocaine	FDA approved	PDGFRA	Sunitinib	FDA approved
EGFR	Panitumumab	FDA approved	PIK3CA	BAY80-6946	FDA approved
EPHA2	Dasatinib	FDA approved	PIK3CA	BKM120	FDA approved
ERBB2	Everolimus	FDA approved	PIK3CA	BYL719	FDA approved
ERBB2	Herceptin	FDA approved	PIK3CA	LY3023414	FDA approved
ERBB2	Lapatinib	FDA approved	RAF1	Sorafenib	FDA approved

Supplementary Note 1

Description of column headers used in Supplementary Data 1

HGNC: Standard gene symbol; HGNC_ID: HUGO Gene Nomenclature Commettee identification number; ENSEMBL: ENSEMBL GenomeENSMBL identification number; GENEID: NCBI Gene identification number; OGTM: Identification of Oncogenes and Tumor suppressors; UNIPROTKB: Uniprot identification number; UNIPROT_ENTRY_NAME: Uniprot entry name; UNIPROT_PROTEIN_NAMES: Uniprot protein names; INSERT SOURCE: Source of the insert DNA; GST FUSION VECTOR: Fusion vector for GST-tagged genes; VENUS FUSION VECTOR: Fusion vector for Venus-tagged genes; GO Cellular localization: Gene Ontology annotations for protein cellular localization; LUAD LUAD ALT CASES: The number of lung adenocarcinoma (TCGA LUAD provisional set) patient samples containing gene alterations; LUAD ALT PERCENT: Percent of lung adenocarcinoma (TCGA LUAD provisional set) patient samples containing gene alterations; LUAD AMP CASES: The number of lung adenocarcinoma (TCGA LUAD provisional set) patient samples DNA amplifications; LUAD AMP PERCENT: Percent of lung adenocarcinoma (TCGA LUAD provisional set) patient samples DNA amplifications; LUAD DEL CASES: The number of lung adenocarcinoma (TCGA LUAD provisional set) patient samples with gene deletions; LUAD DEL PERCENT: Percent of lung adenocarcinoma (TCGA LUAD provisional set) patient samples with gene deletions; LUAD LUAD MUT CASES: The number of lung adenocarcinoma (TCGA LUAD provisional set) patient samples with gene mutations; LUAD LUAD MUT PERCENT: Percent of lung adenocarcinoma (TCGA LUAD provisional set) patient samples with gene mutations; LUAD OVEREXP CASES: The number of lung adenocarcinoma (TCGA LUAD provisional set) patient samples with gene mRNA overexpression; LUAD OVEREXP PERCENT: Percent of lung adenocarcinoma (TCGA LUAD provisional set) patient samples with gene mRNA overexpression; LUAD UNDREXPR CASES: The number of lung adenocarcinoma (TCGA LUAD provisional set) patient samples with gene mRNA underxpression; LUAD UNDREXPR PERCENT: Percent of lung adenocarcinoma (TCGA LUAD provisional set) patient samples with gene mRNA underexpression; LUSC LUSC ALT CASES: The number of lung squamous cell carcinoma (TCGA LUSC provisional set) patient samples containing gene alterations; LUSC ALT PERCENT: Percent of lung squamous cell carcinoma (TCGA LUSC provisional set) patient samples containing gene alterations; LUSC AMP CASES: The number of lung squamous cell carcinoma (TCGA LUSC provisional set) patient samples DNA amplifications; LUSC AMP PERCENT: Percent of lung squamous cell carcinoma (TCGA LUSC provisional set) patient samples DNA amplifications; LUSC DEL CASES: The number of lung squamous cell carcinoma (TCGA LUSC provisional set) patient samples with gene deletions; LUSC DEL PERCENT: Percent of lung squamous cell carcinoma (TCGA LUSC provisional set) patient samples with gene deletions; LUSC LUSC MUT CASES: The number of lung squamous cell carcinoma (TCGA LUSC provisional set) patient samples with gene mutations; LUSC LUSC MUT PERCENT: Percent of lung squamous cell carcinoma (TCGA LUSC provisional set) patient samples with gene mutations; LUSC OVEREXP CASES: The number of lung squamous cell carcinoma (TCGA LUSC provisional set) patient samples with gene mRNA overexpression; LUSC OVEREXP PERCENT: Percent of lung squamous cell carcinoma (TCGA LUSC provisional set) patient samples with gene mRNA overexpression; LUSC UNDREXPR CASES: The number of lung squamous cell carcinoma (TCGA LUSC provisional set) patient samples with gene mRNA underxpression; LUSC UNDREXPR PERCENT: Percent of lung squamous cell carcinoma (TCGA LUSC provisional set) patient samples with gene mRNA underexpression

Description of column headers used in Supplementary Data 2

HUGO1: The HUGO gene symbol of protein 1; HUGO2: The HUGO gene symbol of protein 2; COMMON1: Common name of protein 1; COMMON2: Common name of protein 2; FOC: Fold Over Control value; PVALUE: p-value of permuation test; QVALUE: q-value (p-values adjusted for the multiple comparisons); SS-PPI: Statistically significant protein-protein interactions; HC-PPI: High-confidence protein-protein interactions; OncoPi: OncoPPi set; BFD: PPIs detected in Both Fusion Directions; STRING: PPIs reported in String database; BIOGRID: PPIs reported in BioGrid databse; INTACT: PPIs reported in IntAct database; GENEMANIA: PPIs reported n GeneMania databse; ME_LUAD: In this column, 1 indicates a mutual exclusivity of genomic alterations in lung adenocarcinoma; ME_LOG(OD)_LUAD: Lung adenocarcinoma log odds ratio. Two genes were considered as mutually exclusive if Log(OD) < 0; ME_LUSC: In this column, 1 indicates a mutual exclusivity of genomic alterations in lung squamous cell carcinoma; ME_LUSC_LOG(OD): Lung squamous cell carcinoma log odds ratio. Two genes were considered as mutually exclusive if Log(OD) < 0; FUSION: 1 - PPI is positive in the Fusion analysis; 0 - PPI is negative in Fusion analysis; NA - the Fusion data is not available; COLOCALIZATION: Predicted co-localization; NUM_INTER_DOMAINS: The number of predicted interacting domains; INTERACTING_DOMAINS: Known domain-domain interactions
